# Synthesis of (1,2,3-triazol-4-yl)methyl Phosphinates and (1,2,3-Triazol-4-yl)methyl Phosphates by Copper-Catalyzed Azide-Alkyne Cycloaddition

**DOI:** 10.3390/molecules24112085

**Published:** 2019-05-31

**Authors:** Anna Tripolszky, Krisztina Németh, Pál Tamás Szabó, Erika Bálint

**Affiliations:** 1 Department of Organic Chemistry and Technology, Budapest University of Technology and Economics, 1521 Budapest, Hungary; tripolszky.anna@mail.bme.hu; 2 MS Metabolomics Laboratory, Instrumentation Center, Research Centre for Natural Sciences, Hungarian Academy of Sciences, Magyar Tudósok Krt. 2., H-1117 Budapest, Hungary; nemeth.krisztina.94@ttk.mta.hu (K.N.); szabo.pal@ttk.mta.hu (P.T.S.)

**Keywords:** 1,2,3-triazol, triazolylmethyl phosphinate, triazolylmethyl phosphate, copper-catalyzed azide-alkyne cycloaddition, click reaction

## Abstract

An efficient and practical method was developed for the synthesis of new (1,2,3-triazol-4-yl)methyl phosphinates and (1,2,3-triazol-4-yl)methyl phosphates by the copper(I)-catalyzed azide-alkyne cycloaddition (CuAAC) of organic azides and prop-2-ynyl phosphinate or prop-2-ynyl phosphate. The synthesis of (1-benzyl-1*H*-1,2,3-triazol-4-yl)methyl diphenylphosphinate was optimized with respect to the reaction parameters, such as the temperature, reaction time, and catalyst loading. The approach was applied to a range of organic azides, which confirmed the wide scope and the substituent tolerance of the process. The method elaborated represents a novel approach for the synthesis of the target compounds.

## 1. Introduction

The family of 1,2,3-triazoles has attracted considerable attention in the last decades due to the wide range of their applications in medicine and biochemistry, as well as in materials science [1,2,3]. Several 1,2,3-triazoles proved to be effective antibacterial, antifungal, anticancer, antiviral, or anti-inflammatory agents [4]. The 1,4-disubstituted 1*H*-1,2,3-triazoles also have important applications as agrochemicals, photostabilizers, dyes, or anticorrosives [5,6,7,8].

Organophosphorus compounds also present significant importance as biologically active agents [9]. The 1,2,3-Triazolyl phosphonates combine the advantages of the triazole and the phosphonate moieties [10]. Additionally, several triazolylphosphonate derivatives were found to be suitable for bioconjugation [11,12] or showed an anti-HIV effect [13].

The most convenient synthetic method for the preparation of 1,4-disubstituted 1*H*-1,2,3-triazoles is the Cu(I)-catalyzed 1,3-dipolar (Huisgen) cycloaddition of azides with alkynes (CuAAC) (the “click reaction”), which was developed by Meldal and Sharpless [14,15]. The 1,2,3-Triazolyl phosphonates may be synthesized by the reaction of azides with phosphorus-containing acetylenes [12,13,16,17,18,19,20] or by the cycloaddition of azides incorporating a phosphonate moiety with alkynes [21,22,23,24]. In this paper, reports of the former method will be presented in detail.

**Scheme 1:**
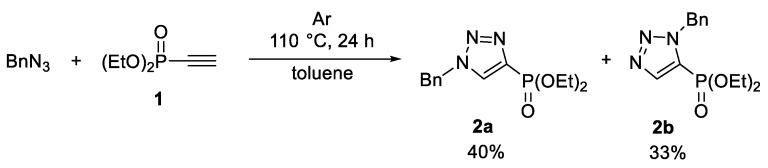
Cycloaddition of benzyl azide and diethyl ethynylphosphonate (**1**).

The 1,3-dipolar cycloaddition of benzyl azide and ethyl ethynylphosphonate (**1**) was carried out in the absence of any catalyst at reflux temperature in toluene (Scheme 1) [16]. The reaction was not selective, two regioisomers, 1,2,3-triazolyl-4-phosphonate (**2a**) and 1,2,3-triazolyl-5-phosphonate (**2b**) were formed.

**Scheme 2:**
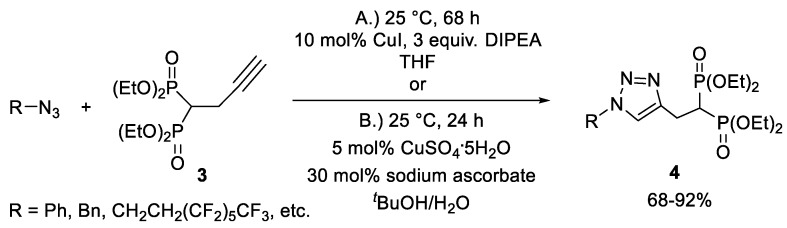
Synthesis of bisphosphonates bearing a triazole ring (**4**) via CuAAc reaction.

Bisphosphonates incorporating a triazole ring (**4**) were prepared by the CuAAC reaction of a propargyl-substituted bisphosphonate (**3**) and organic azides (Scheme 2) [17,18]. The reactions were performed using different methods. According to method A, copper iodide was applied as a catalyst in the presence of *N,N*-diisopropylethylamine (DIPEA) as a base in tetrahydrofuran (THF) [17]. In another case, the Cu(I) catalyst was formed in situ from copper(II) sulfate pentahydrate and sodium ascorbate in the mixture of *tert*-butanol and water [18]. 

**Scheme 3:**
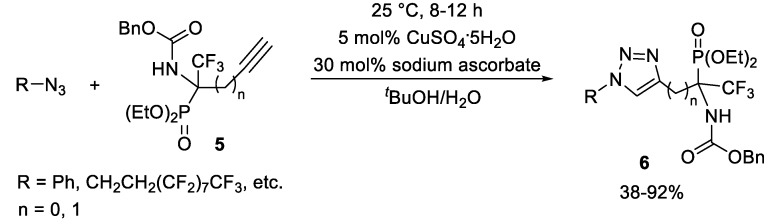
Synthesis of triazole-containing α-CF_3_-α-aminophosphonates (**6**).

Röschenthaler and co-workers performed the synthesis of triazole-containing α-CF_3_-α-aminophosphonates (**6**) by the Cu(I)-catalyzed cycloaddition of azides and ethynyl- or propargyl-substituted phosphonate (**5**) (Scheme 3) [19].

**Scheme 4:**
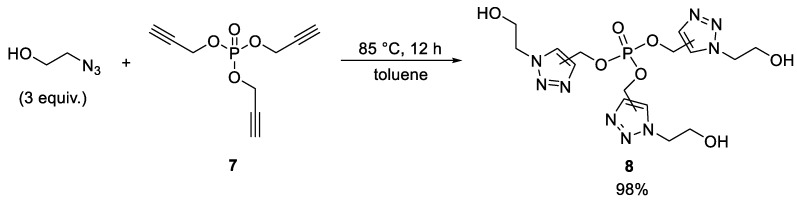
Synthesis of a triazole-functionalized phosphate monomer (**8**).

The click reaction of 2-azidoethanol and triprop-2-ynyl phosphate (**7**) was also reported, in which a triazole-functionalized phosphate flame-retardant monomer (**8**) was synthesized (Scheme 4) [20].

To the best of our knowledge, there is no example for the azide-alkyne cycloaddition of simple azides and prop-2-ynyl phosphinate or diethyl prop-2-ynyl phosphate. The reaction of prop-2-ynyl diphenylphosphinate was reported only with steroidal azides [25]. Hence, we set a sight on building a phosphinate or phosphate side-chain on position 4 of the triazole ring by the click reaction with simple azides.

In this paper, we report on an efficient and fast synthesis of (1,2,3-triazol-4-yl)methyl phosphinates and (1,2,3-triazol-4-yl)methyl phosphates by the copper(I)-catalyzed 1,3-dipolar (Huisgen) cycloaddition of organic azides and prop-2-ynyl phosphinates or diethyl prop-2-ynyl phosphate.

## 2. Results and Discussion

**Scheme 5:**
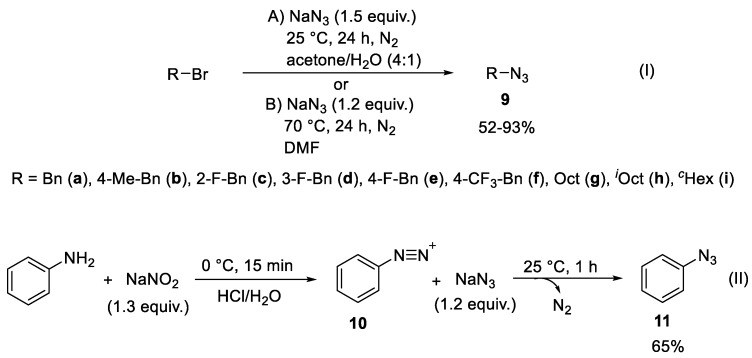
Synthesis of organic azides (**9a–i** and **11**).

At first, the starting materials of the cycloadditions were prepared. The synthesis of azides was carried out based on the literature data [26,27] (Scheme 5). The benzyl or substituted benzyl bromides were reacted with 1.5 equivalents of sodium azide at room temperature for 24 h in a mixture of acetone/water in a ratio of 4:1, and the corresponding azides (**9a–f**) were obtained in yields of 80–93% (Scheme 5/I, Method A). The synthesis of octyl-, *i*-octyl- and cyclohexyl azides (**9g–i**) was performed using 1.2 equivalents of sodium azide at 70 °C in dimethylformamide (DMF) (Scheme 5/I, Method B). For the preparation of phenyl azide (**11**), aniline was reacted with sodium nitrite in HCl/H_2_O solution at 0 °C for 15 min, and the diazonium salt (**10**) formed was further reacted with sodium azide at ambient temperature (Scheme 5/II). After workup, phenyl azide (**11**) was obtained in a yield of 65%.

**Scheme 6:**
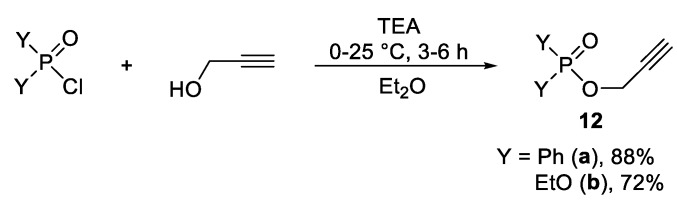
Synthesis of prop-2-ynyl phosphinate (**12a**) and diethyl prop-2-ynyl phosphate (**12d**).

The synthesis of the prop-2-ynyl diphenylphosphinate (**12a**) and the diethyl prop-2-ynyl phosphate (**12b**) was carried out by the reaction of diphenylphosphinic chloride or diethyl chlorophosphate with propargyl alcohol in the presence of triethylamine in diethyl ether (Scheme 6). The phosphorus-containing alkynes (**12a** and **12b**) were isolated in yields of 88% and 72%, respectively.

As the next step of our work, the 1,3-dipolar cycloaddition of benzyl azide (**9a**) and prop-2-ynyl diphenylphosphinate (**12a**) was investigated in the presence of copper(II) sulfate pentahydrate and sodium ascorbate in the mixture of *tert*-butanol/water (4:1) (Table 1). Performing the reaction at room temperature using 5 mol% of CuSO_4_**·**5H_2_O and 30 mol% of sodium ascorbate, it was complete after 1 h and, after column chromatography, the desired (1-benzyl-1*H*-1,2,3-triazol-4-yl)methyl diphenylphosphinate (**13a**) was obtained in a yield of 84% (Table 1, Entry 1). Decreasing the amount of the reducing agent (sodium ascorbate) to 10 mol%, the conversion was only 78% under the conditions applied before (25 °C, 60 min) (Table 1, Entry 2). In this case, complete conversion could be reached after 3 h (Table 1, Entry 3). Carrying out the reaction at 60 °C for 5 min under conventional heating, the triazolylmethyl phosphinate (**13a**) was formed in a conversion of 93% (Table 1, Entry 4). Applying microwave (MW) irradiation, the result obtained was similar (Table 1, Entry 5), thus further experiments were performed in an oil bath. Increasing the reaction time to 10 min, the cycloaddition was complete and product **13a** was isolated in a yield of 89% (Table 1, Entry 6). In the next step, the effect of the catalyst loading was studied (Table 1/Entries 7–9). Using 3% of CuSO_4_**·**5H_2_O and 5 mol% of sodium ascorbate, the reaction was similar to the previous experiment (Table 1, Entries 6 and 7). Upon decreasing the amount of the Cu(II) catalyst to 2%, the conversion was lower (90%) (Table 1, Entry 7). Based on the results obtained, a temperature of 60 °C, a reaction time of 10 min and application of 3 mol% of CuSO_4_**·**5H_2_O, as well as 5 mol% of sodium ascorbate, were found to be the optimum parameters (Table 1, Entry 7).

**Scheme 7:**
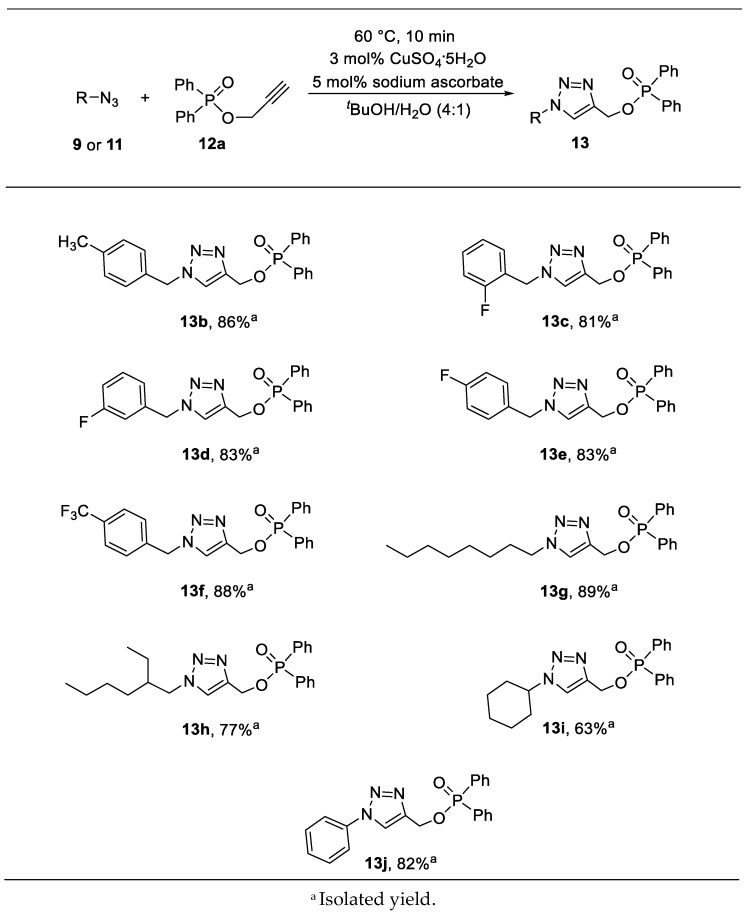
Synthesis of (1*H*-1,2,3-triazol-4-yl)methyl diphenylphosphinates (**13**).

In the next series of experiments, the cycloaddition of prop-2-ynyl diphenylphosphinate (**12a**) with a wide range of organic azides (**9** and **11**) was studied under the optimized conditions (Scheme 7). The reactions were complete in all cases. Using 4-methylbenzyl azide (**9b**), the [1-(4-methylbenzyl)-1*H*-1,2,3-triazol-4-y])methyl diphenylphosphinate (**13b**) was isolated in a yield of 86%. Changing for fluoro-substituted benzyl azides (2-, 3- or 4-fluorobenzyl azide) (**9c–e**), the desired triazolylmethyl phosphinates (**13c–e**) were obtained in yields of 81–83% after column chromatography. Carrying out the reaction starting from 4-(trifluoromethyl)benzyl azide (**9f**), the product **13f** was prepared in a yield of 88%. Applying octyl azide (**9g**), the corresponding (1-octyl-1*H*-1,2,3-triazol-4-yl)methyl diphenylphosphinate (**13g**) was obtained in a yield of 89%, while using *iso*-octyl azide (**9h**), product **13h** was isolated in a yield of 77%. The cyclohexyl azide (**9i**) was also tried out as the azide component; however, the triazolylmethyl phosphinate (**13i**) could be obtained in a slightly lower yield (62%) due to the steric effects of the cyclohexyl group. Finally, the click reaction of phenyl azide (**11**) was performed, and the desired triazolylmethyl phosphinate (**13j**) was synthesized in a yield of 82%.

The cycloaddition of benzyl azide (**9a**) and diethyl prop-2-ynyl phosphate (**12b**) was also investigated (Table 2). Using the optimized conditions (3 mol% of CuSO_4_**·**5H_2_O and 5 mol% of sodium ascorbate, 60 °C, 10 min), the reaction was incomplete, and the (1-benzyl-1*H*-1,2,3-triazol-4-yl)methyl diethyl phosphate (**14a**) was formed in a conversion of only 48% (Table 2, Entry 1). Increasing the reaction time to 20 min, the conversion increased to 59%; however, after 30 min, the reaction was complete, and the triazolylmethyl phosphate (**14a**) was obtained in a yield of 75% (Table 2, Entries 2 and 3). The diethyl prop-2-ynyl phosphate (**12b**) proved to be somewhat less reactive in the click reaction as compared to the prop-2-ynyl diphenylphosphinate (**12a**).

**Scheme 8:**
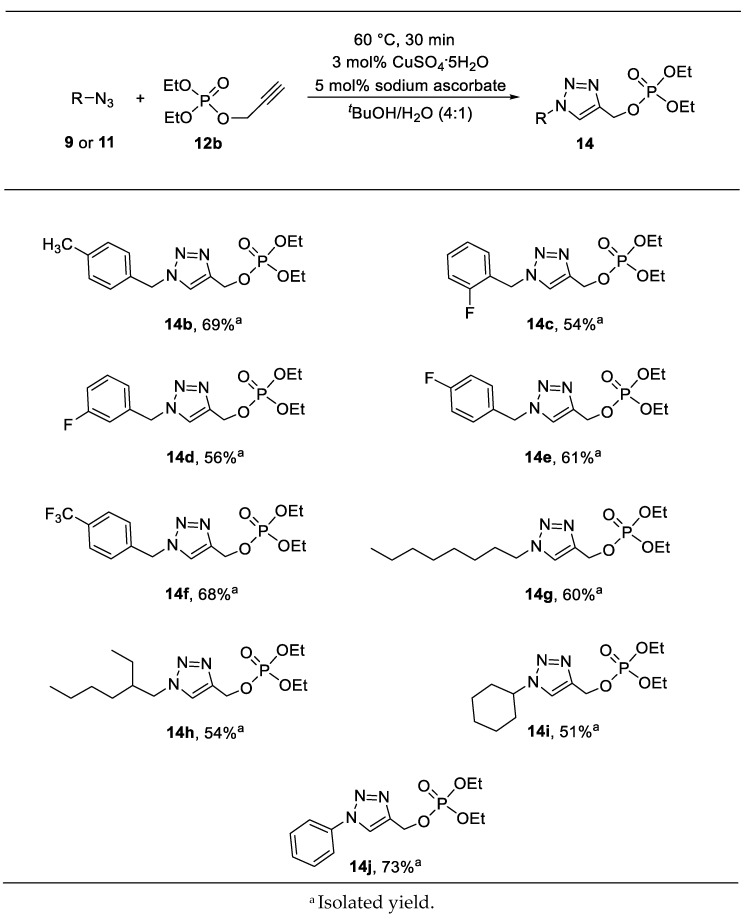
Synthesis of (1*H*-1,2,3-triazol-4-yl)methyl diethyl phosphates (**14**).

In the next round, the reaction of diethyl prop-2-ynyl phosphate (**12b**) was also carried out with a wide range of organic azides **(9** and **11**) in the presence of 3 mol% of CuSO_4_**·**5H_2_O and 5 mol% of sodium ascorbate at 60 °C for 30 min (Scheme 8). Applying substituted benzyl azides (4-methyl-, 2-, 3- or 4-fluorobenzylazide and 4-(trifluoromethyl)benzyl azide) (**11b–f**), the corresponding (1-benzyl-1*H*-1,2,3-triazol-4-yl)methyl diethyl phosphate derivatives (**14b–f**) were prepared in yields of 54–69%. Performing the cycloaddition starting from octyl or *iso*-octyl azide (**11g** or **11h**), the desired products (**14g** or **14h**) were isolated in yields of 60% and 54%, respectively. The reaction of cyclohexyl azide (**11i**) and diethyl prop-2-ynyl phosphate (**12b**) was also carried out, and the product **14i** was obtained in a yield of 51%. Using aromatic azide, such as phenyl azide (**11**), the triazolylmethyl phosphate (**14j**) was synthesized in a yield of 77%.

## 3. Materials and Methods 

### 3.1. General

The reactions under conventional heating were carried out in an oil bath. The microwave-assisted experiments were performed in a 300 W CEM Discover focused microwave reactor (CEM Microwave Technology Ltd., Buckingham, UK) equipped with a pressure controller using 5–10 W irradiation under isothermal conditions.

High-performance liquid chromatography-mass spectrometry (HPLC–MS) measurements were performed with an Agilent 1200 liquid chromatography system coupled with a 6130 quadrupole mass spectrometer equipped with an ESI ion source (Agilent Technologies, Palo Alto, CA, USA). Analysis was performed at 40 °C on a Gemini C18 column (150 mm × 4.6 mm, 3 µm; Phenomenex, Torrance, CA, USA) with a mobile phase flow rate of 0.6 mL/min. The composition of eluent A was 0.1% (NH_4_)(HCOO) in water; eluent B was 0.1% (NH_4_)(HCOO) and 8% water in acetonitrile, 0–3 min 5% B, 3–13 min gradient, 13–20 min 100% B. The injection volume was 5 µL. The chromatographic profile was registered at 254 nm. The MSD operating parameters were as follows: positive ionization mode, scan spectra from m/z 120 to 1200, drying gas temperature 300 °C, nitrogen flow rate 10 L/min, nebulizer pressure 60 psi, capillary voltage 4000 V.

High-resolution mass spectrometric measurements were performed using a Sciex 5600+ Q-TOF (time-of-flight) mass spectrometer in positive electrospray mode.

The ^31^P, ^1^H, ^13^C, NMR spectra were taken in CDCl_3_ solution on a Bruker AV-300 spectrometer (Bruker AXS GmBH, Karlsruhe, Germany) operating at 121.5, 75.5, and 300 MHz, respectively. Chemical shifts are downfield relative to 85% H_3_PO_4_ and TMS (spectra for all compounds synthesized can be found in Supplementary).

### 3.2. General Procedure for the Synthesis of Benzyl Azides (Method A)

To a stirred solution of 10.0 mmol alkyl halides (1.19 mL of benzyl bromide, 1.85 g of 4-methylbenzyl bromide, 1.21 mL of 2-fluorobenzyl bromide, 1.23 mL of 3-fluorobenzyl bromide, 1.25 mL of 4-fluorobenzyl bromide, or 1.55 mL of 4-(trifluoromethyl)benzyl bromide) in 100 mL of acetone/H_2_O 4:1 (*v/v*) was added 15.0 mmol (0.98 g) of sodium azide. The reaction mixture was stirred at room temperature for 24 h. After, the reaction was extracted with Et_2_O (3 × 50 mL), dried over Na_2_SO_4_, and concentrated under reduced pressure to give benzyl azides as pale yellow oils. The following azides were thus prepared (Table 3):

### 3.3. General Procedure for the Synthesis of Alkyl Azides (Method B)

To a stirred solution of 10.0 mmol alkyl halides (1.76 mL of octyl bromide, 1.78 mL of *iso*-octyl bromide, or 1.23 mL of bromocyclohexane) in 20 mL of DMF, 12.0 mmol (0.78 g) of sodium azide was added. The reaction mixture was stirred at 70 °C for 24 h in an oil bath. After, the reaction was extracted with Et_2_O (3 × 50 mL), dried over Na_2_SO_4_, and concentrated under reduced pressure to give alkyl azides as pale yellow oils. The following azides were thus prepared (Table 4):

### 3.4. General Procedure for the Synthesis of Phenyl Azide

To a stirred solution of 0.46 mL aniline (5.0 mmol) in 25 mL 17% HCl solution at 0 °C, 0.51 g (7.5 mmol) of sodium nitrite in water (3 mL) was added. After stirring for 15 min, a solution of 0.32 g sodium azide (7.5 mmol) in water (3 mL) was carefully added. The reaction was left to stir for 1 h, followed by extraction with Et_2_O (3 × 30 mL). The combined organic layers were dried over Na_2_SO_4_, and carefully concentrated under reduced pressure to give phenyl azide as an orange oil.

*Phenyl azide* (**11**) [26]: Yield: 65% (0.77 g) of compound **11** as orange oil; [M+H]^+^_found_ = 120.0567, C_6_H_6_N_3_ requires 120.0562.

### 3.5. General Procedure for the Synthesis of Prop-2-ynyl Diphenylphosphinate and Diethyl Prop-2-ynyl Phosphate 

To a stirred solution of 10 mmol of diphenylphosphinic chloride (1.91 mL) or diethyl chlorophosphate (1.44 mL) in 10 mL of Et_2_O, 1.39 mL (10 mmol) of Et_3_N and 0.50 mL (10.0 mmol) of propargyl alcohol at 0 °C were added under a nitrogen atmosphere. The solution was left stirring at room temperature for 3–6 h and the reaction mixture obtained was passed through a 1 cm silica gel layer using Et_2_O. After evaporating the solvent, the products were obtained as white crystals (**12a**) or colorless oil (**12b**). The following products were thus prepared (Table 5):

### 3.6. General Procedure for the Synthesis of (1H-1,2,3-Triazol-4-yl)methyl Phosphinates or Diethyl Phosphates

The 1.0 mmol organic azide (0.13 g of benzyl azide, 0.15 g of 4-methylbenzyl azide, 0.15 g of 2-fluorobenzyl azide, 0.15 g of 3-fluorobenzyl azide, 0.15 g of 4-fluorobenzyl azide, 0.20 g of 4-(trifluoromethyl)benzyl azide, 0.16 g of octyl azide, 0.16 g of *iso*-octyl azide, 0.13 g of cyclohexyl azide or 0.12 g of phenyl azide) and 1.0 mmol acetylene (0.26 g of prop-2-ynyl diphenylphosphinate or 0.19 g of diethyl prop-2-ynyl phosphate) were suspended in a mixture of *^t^*BuOH/H_2_O (4:1) (2 mL). To this 7.5 mg (0.03 mmol) of CuSO_4_·5H_2_O and 20 mg (0.1 mmol) of sodium ascorbate were added. The mixture was stirred at 60 °C for 10 min. The resulting solution was extracted with ethyl acetate (3 × 30 mL) and the combined organic layers were dried over Na_2_SO_4_. After evaporating the solvent, the crude product was purified by column chromatography using silica gel and dichloromethane/methanol 97:3 as the eluent. The following products were thus prepared:

*(1-Benzyl-1H-1,2,3-triazol-4-yl)methyl diphenylphosphinate* (**13a**): Yield: 91% (0.35 g), white crystals; Mp: 91-92 °C; ^31^P NMR (CDCl_3_) δ 33.5; ^1^H NMR (CDCl_3_) δ 5.18 (d, ^3^*J*_HP_ = 9.1, 2H, CH_2_O), 5.46 (s, 2H, CH_2_Ph), 7.20–7.27 (m, 2H, C_2_H), 7.32–7.37 (m, 3H, C_3_H, C_4_H), 7.38–7.44 (m, 4H, C_3’_H), 7.47–7.54 (m, 2H, C_4’_H), 7.52 (s, 1H, CH), 7.79 (dd, ^3^*J*_HP_ = 11.8, ^3^*J*_HH_ = 7.4, 4H, C_2’_H); ^13^C NMR (CDCl_3_) δ 54.3 (CH_2_Ph), 58.2 (d, ^2^*J*_CP_ = 5.3, CH_2_O), 123.8 (CH=), 128.3 (C_2_), 128.7 (d, ^3^*J*_CP_ = 13.3, C_3’_), 128.8 (C_4_), 129.3 (C_3_), 131.2 (d, ^1^*J*_CP_ = 137.0, C_1’_), 131.8 (d, ^2^*J*_CP_ = 10.3, C_2’_), 132.5 (d, *J*_CP_ = 2.8, C_4’_), 134.5 (C_1_), 144.0 (d, ^3^*J*_CP_ = 6.7, C=); [M+H]^+^_found_ = 390.1363., C_22_H_21_N_3_O_2_P requires 390.1371.

*[1-(4-Methylbenzyl)-1H-1,2,3-triazol-4-yl]methyl diphenylphosphinate* (**13b**): Yield: 86% (0.34 g), white crystals; Mp: 119-121 °C; ^31^P NMR (CDCl_3_) δ 33.3; ^1^H NMR (CDCl_3_) δ 2.35 (s, 3H, CH_3_Ph), 5.18 (d, ^3^*J*_HP_ = 9.0, 2H, CH_2_O), 5.41 (s, 2H, CH_2_Ph), 7.07–7.24 (m, 4H, C_3’_H), 7.32–7.60 (m, 7H, C_2_H, C_3_H, C_4’_H), 7.79 (s, 1H, CH), 7.80 (dd, ^3^*J*_HP_ = 12.4, ^3^*J*_HH_ = 7.4, 4H, C_2’_H); ^13^C NMR (CDCl_3_) δ 21.3 (CH_3_Ph), 54.2 (CH_2_Ph), 58.2 (d, ^2^*J*_CP_ = 5.3, CH_2_O), 123.6 (CH=), 128.3 (C_2_), 128.7 (d, ^3^*J*_CP_ = 13.2, C_3’_), 129.9 (C_3_), 131.3 (d, ^1^*J*_CP_ = 136.6, C_1’_), 131.4 (C_1_), 131.8 (d, ^2^*J*_CP_ = 10.3, C_2’_), 132.4 (d, *J*_CP_ = 2.8, C_4’_), 138.9 (C_4_), 144.0 (d, ^3^*J*_CP_ = 6.7, C=); [M+H]^+^_found_ = 404.1519, C_23_H_23_N_3_O_2_P requires 404.1527.

*[1-(2-Fluorobenzyl)-1H-1,2,3-triazol-4-yl]methyl diphenylphosphinate* (**13c**): Yield: 81% (0.33 g), pale yellow crystals; Mp: 91-93 °C; ^31^P NMR (CDCl_3_) δ 33.5; ^1^H NMR (CDCl_3_) δ 5.18 (d, ^3^*J*_HP_ = 8.9, 2H, CH_2_O), 5.52 (s, 2H, CH_2_Ph), 7.03–7.17 (m, 2H, C_3_H, C_5_H), 7.18–7.26 (m, 2H, C_6_H), 7.28–7.53 (m, 7H, C_4_H, C_3’_H, C_4’_H), 7.60 (s, 1H, CH), 7.79 (dd, ^3^*J*_HP_ = 12.3, ^3^*J*_HH_ = 6.9, 4H, C_2’_H); ^13^C NMR (CDCl_3_) δ 47.8 (d, ^3^*J*_CF_ = 4.4, CH_2_Ph), 58.1 (d, ^2^*J*_CP_ = 5.3, CH_2_O), 115.9 (d, ^2^*J*_CF_ = 21.1, C_3_), 121.8 (d, ^2^*J*_CF_ = 14.6, C_1_), 124.0 (CH=), 124.9 (d, *J*_CF_ = 3.8, C_5_), 128.7 (d, ^3^*J*_CP_ = 13.3, C_3’_), 130.7 (d, ^3^*J*_CF_ = 3.2, C_4_), 131.1 (d, ^3^*J*_CF_ = 8.2, C_6_), 131.1 (d, ^1^*J*_CP_ = 136.8, C_1’_), 131.8 (d, ^2^*J*_CP_ = 10.4, C_2’_), 132.4 (d, *J*_CP_ = 2.9, C_4’_), 144.0 (d, ^3^*J*_CP_ = 6.6, C=), 160.0 (d, ^1^*J*_CF_ = 248.1, C_2_); [M+H]^+^_found_ = 408.1268, C_22_H_20_N_3_O_2_FP requires 408.1277.

*[1-(3-Fluorobenzyl)-1H-1,2,3-triazol-4-yl]methyl diphenylphosphinate* (**13d**): Yield: 83% (0.34 g), pale yellow crystals; Mp: 93-94 °C; ^31^P NMR (CDCl_3_) δ 33.7; ^1^H NMR (CDCl_3_) δ 5.20 (d, ^3^*J*_HP_ = 9.3, 2H, CH_2_O), 5.46 (s, 2H, CH_2_Ph), 6.87–6.96 (m, 1H, C_4_H), 6.89–7.11 (m, 2H, C_2_H, C_6_H), 7.26–7.36 (m, 1H, C_5_H), 7.37–7.55 (m, 6H, C_3’_H, C_4’_H), 7.59 (s, 1H, CH), 7.79 (dd, ^3^*J*_HP_ = 12.4, ^3^*J*_HH_ = 6.8, 4H, C_2’_H); ^13^C NMR (CDCl_3_) δ 53.6 (d, *J*_CF_ = 2.0, CH_2_Ph), 58.2 (d, ^2^*J*_CP_ = 5.3, CH_2_O), 115.2 (d, ^2^*J*_CF_ = 22.2, C_4_), 116.0 (d, ^2^*J*_CF_ = 21.0, C_2_), 123.8 (d, *J*_CF_ = 3.1, C_6_), 123.9 (CH=), 128.7 (d, ^3^*J*_CP_ = 13.3, C_3’_), 130.9 (d, ^3^*J*_CF_ = 8.3, C_5_), 131.1 (d, ^1^*J*_CP_ = 136.7, C_1’_), 131.8 (d, ^2^*J*_CP_ = 10.3, C_2’_), 132.5 (d, *J*_CP_ = 2.8, C_4’_), 136.8 (d, ^3^*J*_CF_ = 7.4, C_1_), 144.2 (d, ^3^*J*_CP_ = 6.5, C=), 163.1 (d, ^1^*J*_CF_ = 248.0, C_3_); [M+H]^+^_found_ = 408.1269, C_22_H_20_N_3_O_2_FP requires 408.1277.

*[1-(4-Fluorobenzyl)-1H-1,2,3-triazol-4-yl]methyl diphenylphosphinate* (**13e**): Yield: 83% (0.34 g), pale yellow crystals; Mp: 95-97 °C; ^31^P NMR (CDCl_3_) δ 33.5; ^1^H NMR (CDCl_3_) δ 5.18 (d, ^3^*J*_HP_ = 9.2, 2H, CH_2_O), 5.43 (s, 2H, CH_2_Ph), 7.04 (t, *J*_HF_ = ^3^*J*_HH_ = 8.6, 2H, C_2_H), 7.23 (dd, ^3^*J*_HF_ = 8.4, ^3^*J*_HH_ = 5.1, 2H, C_3_H), 7.33–7.54 (m, 6H, C_3’_H, C_4’_H), 7.55 (s, 1H, CH), 7.79 (dd, ^3^*J*_HP_ = 12.4, ^3^*J*_HH_ = 7.4, 4H, C_2’_H); ^13^C NMR (CDCl_3_) δ 53.5 (CH_2_Ph), 58.2 (d, ^2^*J*_CP_ = 5.3, CH_2_O), 116.2 (d, ^2^*J*_CF_ = 21.9, C_3_), 123.8 (CH=), 128.7 (d, ^3^*J*_CP_ = 13.3, C_3’_), 130.1 (d, ^3^*J*_CF_ = 8.4, C_2_), 130.4 (d, *J*_CF_ = 3.3, C_1_), 131.2 (d, ^1^*J*_CP_ = 136.7, C_1’_), 131.8 (d, ^2^*J*_CP_ = 10.3, C_2’_), 132.5 (d, *J*_CP_ = 2.9, C_4’_), 144.2 (d, ^3^*J*_CP_ = 6.2, C=), 163.0 (d, ^1^*J*_CF_ = 248.3, C_4_); [M+H]^+^_found_ = 408.1268, C_22_H_20_N_3_O_2_FP requires 408.1277.

*[1-(4-Trifluoromethyl)benzyl)-1H-1,2,3-triazol-4-yl]methyl diphenylphosphinate* (**13f**): Yield: 88% (0.40 g), white crystals; Mp: 124-125 °C; ^31^P NMR (CDCl_3_) δ 33.6; ^1^H NMR (CDCl_3_) δ 5.20 (d, ^3^*J*_HP_ = 9.5, 2H, CH_2_O), 5.43 (s, 2H, CH_2_Ph), 7.33 (d, ^3^*J*_HH_ = 8.0, 2H, C_2_H), 7.39–7.46 (m, 4H, C_3’_H), 7.48–7.53 (m, 2H, C_4’_H), 7.61 (d, ^3^*J*_HH_ = 8.0, 2H, C_3_H), 7.63 (s, 1H, CH), 7.79 (dd, ^3^*J*_HP_ = 12.4, ^3^*J*_HH_ = 6.9, 4H, C_2’_H); ^13^C NMR (CDCl_3_) δ 53.6 (CH_2_Ph), 58.2 (d, ^2^*J*_CP_ = 5.4, CH_2_O), 122.8 (CH=), 123.9 (q, ^1^*J*_CF_ = 272.1, CF_3_), 126.2 (q, ^3^*J*_CF_ = 3.7, C_3_), 128.4 (C_2_), 128.7 (d, ^3^*J*_CP_ = 13.3, C_3’_), 131.1 (q, ^2^*J*_CF_ =32.9, C_4_), 131.2 (d, ^1^*J*_CP_ = 136.7, C_1’_), 131.7 (d, ^2^*J*_CP_ = 10.3, C_2’_), 132.5 (d, *J*_CP_ = 2.9, C_4’_), 138.5 (C_1_), 144.4 (d, ^3^*J*_CP_ = 6.0, C=); [M+H]^+^_found_ = 458.1242, C_23_H_20_N_3_O_2_F_3_P requires 458.1245.

*(1-Octyl-1H-1,2,3-triazol-4-yl)methyl diphenylphosphinate* (**13g**): Yield: 89% (0.37 g), yellow oil; ^31^P NMR (CDCl_3_) δ 33.2; ^1^H NMR (CDCl_3_) δ 0.87 (t, ^3^*J*_HH_ = 6.5, 3H, CH_3_), 1.10–1.41 (m, 10H, CH_2_CH_2_(C*H*_2_)_5_CH_3_), 1.72–1.97 (m, 2H, CH_2_C*H*_2_(CH_2_)_5_CH_3_), 4.28 (t, 2H, ^3^*J*_HH_ = 7.3, C*H*_2_CH_2_(CH_2_)_5_CH_3_), 5.21 (d, ^3^*J*_HP_ = 9.1, 2H, CH_2_O), 7.34–7.57 (m, 6H, C_3_H, C_4_H), 7.60 (s, 1H, CH), 7.82 (dd, ^3^*J*_HP_ = 12.4, ^3^*J*_HH_ = 7.3, 4H, C_2_H); ^13^C NMR (CDCl_3_) δ 14.2 (CH_3_), 22.7 (*C*H_2_CH_3_), 26.6 (*C*H_2_CH_2_CH_3_), 29.0 (*C*H_2_(CH_2_)_2_CH_3_), 29.1 (*C*H_2_(CH_2_)_3_CH_3_), 30.3 (*C*H_2_(CH_2_)_4_CH_3_), 31.8 (*C*H_2_(CH_2_)_5_CH_3_), 50.5 (*C*H_2_(CH_2_)_6_CH_3_), 58.3 (d, ^2^*J*_CP_ = 5.3, CH_2_O), 123.7 (CH=), 128.7 (d, ^3^*J*_CP_ = 13.2, C_3_), 131.4 (d, ^1^*J*_CP_ = 136.6, C_1_), 131.8 (d, ^2^*J*_CP_ = 10.3, C_2_), 132.4 (d, *J*_CP_ = 2.8, C_4_), 143.6 (d, ^3^*J*_CP_ = 6.9, C=); [M+H]^+^_found_ = 412.2154, C_23_H_31_N_3_O_2_P requires 412.2153.

*(1-Iso-octyl-1H-1,2,3-triazol-4-yl)methyl diphenylphosphinate* (**13h**): Yield: 77% (0.49 g), yellow oil; ^31^P NMR (CDCl_3_) δ 33.3; ^1^H NMR (CDCl_3_) δ 0.89 (t, ^3^*J*_HH_ = 6.7, 6H, CH_3_), 1.02–1.41 (m, 8H, CH(C*H*_2_)_3_CH_3_, CHC*H*_2_CH_3_), 1.74–1.97 (m, 1H, NCH_2_C*H*), 4.21 (d, 2H, ^3^*J*_HH_ = 6.9, NC*H*_2_CH), 5.22 (d, ^3^*J*_HP_ = 9.0, 2H, CH_2_O), 7.34–7.58 (m, 6H, C_3_H, C_4_H), 7.59 (s, 1H, CH), 7.82 (dd, ^3^*J*_HP_ = 12.5, ^3^*J*_HH_ = 7.4, 4H, C_2_H); ^13^C NMR (CDCl_3_) δ 10.5 (NCH_2_CHCH_2_*C*H_3_), 14.0 (CH_3_), 22.9 (*C*H_2_CH_3_), 23.7 (NCH_2_CH*C*H_2_CH_3_), 28.5 (*C*H_2_CH_2_CH_3_), 30.4 (*C*H_2_(CH_2_)_2_CH_3_), 40.3 (NCH_2_*C*HCH_2_CH_3_), 53.6 (N*C*H_2_CHCH_2_CH_3_), 58.2 (d, ^2^*J*_CP_ = 5.3, CH_2_O), 124.2 (CH=), 128.6 (d, ^3^*J*_CP_ = 13.3, C_3_), 131.3 (d, ^1^*J*_CP_ = 136.7, C_1_), 131.7 (d, ^2^*J*_CP_ = 10.3, C_2_), 132.4 (d, *J*_CP_ = 2.9, C_4_), 143.4 (d, ^3^*J*_CP_ = 6.2, C=); [M+H]^+^_found_ = 412.2154, C_23_H_31_N_3_O_2_P requires 412.2153.

*(1-Cyclohexyl-1H-1,2,3-triazol-4-yl)methyl diphenylphosphinate* (**13i**): Yield: 63% (0.24 g), white crystals; Mp: 122-124 °C; ^31^P NMR (CDCl_3_) δ 33.2; ^1^H NMR (CDCl_3_) δ 1.16–1.52 (m, 4H, C_3_H_ax_, C_4_H_ax_, C_4_H_eq_), 1.58–1.80 (m, 2H, C_3_H_eq_), 1.81–1.95 (m, 2H, C_2_H_ax_), 2.01–2.22 (m, 2H, C_2_H_eq_), 4.29–4.45 (m, 1H, C_1_H), 5.21 (d, ^3^*J*_HP_ = 9.0, 2H, CH_2_O), 7.32–7.56 (m, 6H, C_3’_H, C_4’_H), 7.59 (s, 1H, CH), 7.82 (dd, ^3^*J*_HP_ = 12.4, ^3^*J*_HH_ = 7.3, 4H, C_2’_H); ^13^C NMR (CDCl_3_) δ 25.2 (C_4_), 25.3 (C_3_), 33.6 (C_2_), 58.4 (d, ^2^*J*_CP_ = 5.3, CH_2_O), 60.3 (C_1_) 121.6 (CH=), 128.7 (d, ^3^*J*_CP_ = 13.2, C_3’_), 131.4 (d, ^1^*J*_CP_ = 136.9, C_1’_), 131.8 (d, ^2^*J*_CP_ = 10.3, C_2’_), 132.4 (d, *J*_CP_ = 2.8, C_4’_), 143.1 (d, ^3^*J*_CP_ = 6.5, C=); [M+H]^+^_found_ = 382.1677, C_21_H_25_N_3_O_2_P requires 382.1684.

*(1-Phenyl-1H-1,2,3-triazol-4-yl)methyl diphenylphosphinate* (**13j**): Yield: 82% (0.31 g), white crystals; Mp: 121-122 °C; ^31^P NMR (CDCl_3_) δ 33.5; ^1^H NMR (CDCl_3_) δ 5.30 (d, ^3^*J*_HP_ = 9.1, 2H, CH_2_O), 7.34–7.57 (m, 9H, C_3_H, C_4_H, C_3’_H, C_4’_H), 7.68 (d, ^3^*J*_HH_ = 7.7, 2H, C_2_H), 7.85 (dd, ^3^*J*_HP_ = 12.4, ^3^*J*_HH_ = 7.4, 4H, C_2’_H), 8.05 (s, 1H, CH); ^13^C NMR (CDCl_3_) δ 58.2 (d, ^2^*J*_CP_ = 5.3, CH_2_O), 120.7 (C_2_), 122.2 (CH=), 128.8 (d, ^3^*J*_CP_ = 13.2, C_3’_), 129.0 (C_4_), 129.9 (C_3_), 131.2 (d, ^1^*J*_CP_ = 136.6, C_1’_), 131.8 (d, ^2^*J*_CP_ = 10.4, C_2’_), 132.5 (d, *J*_CP_ = 2.9, C_4’_), 137.0 (C_1_), 144.4 (d, ^3^*J*_CP_ = 6.0, C=); [M+H]^+^_found_ = 376.1210, C_21_H_19_N_3_O_2_P requires 376.1214.

*(1-Benzyl-1H-1,2,3-triazol-4-yl)methyl diethyl phosphate* (**14a**): Yield: 75% (0.24 g), pale yellow oil; ^31^P NMR (CDCl_3_) δ –1.0; ^1^H NMR (CDCl_3_) δ 1.27 (t, ^3^*J*_HH_ = 6.9, 6H, OCH_2_C*H*_3_), 4.01–4.09 (m, 4H, OC*H*_2_CH_3_), 5.16 (d, ^3^*J*_HP_ = 9.4, 2H, CH_2_O), 5.53 (s, 2H, CH_2_Ph), 7.25–7.30 (m, 2H, C_2_H), 7.32–7.40 (m, 3H, C_3_H, C_4_H), 7.62 (s, 1H, CH); ^13^C NMR (CDCl_3_) δ 16.1 (d, ^3^*J*_CP_ = 6.3, OCH_2_*C*H_3_), 54.4 (CH_2_Ph), 60.6 (d, ^2^*J*_CP_ = 6.2, CH_2_O), 64.1 (d, ^2^*J*_CP_ = 6.4, O*C*H_2_CH_3_), 123.5 (CH=), 128.2 (C_2_), 129.0 (C_4_), 129.2 (C_3_), 134.5 (C_1_), 143.8 (d, ^3^*J*_CP_ = 8.2, C=); [M+H]^+^_found_ = 326.1259, C_14_H_21_N_3_O_4_P requires 326.1269.

*[1-(4-Methylbenzyl)-1H-1,2,3-triazol-4-yl]methyl diethyl phosphate* (**14b**): Yield: 69% (0.23 g), pale yellow oil; ^31^P NMR (CDCl_3_) δ –1.1; ^1^H NMR (CDCl_3_) δ 1.17 (t, ^3^*J*_HH_ = 6.9, 6H, OCH_2_C*H*_3_), 2.27 (s, 3H, CH_3_Ph), 3.87–4.05 (m, 4H, OC*H*_2_CH_3_), 5.07 (d, ^3^*J*_HP_ = 9.2, 2H, CH_2_O), 5.40 (s, 2H, CH_2_Ph), 7.10 (s, 4H, C_2_H, C_3_H), 7.52 (s, 1H, CH); ^13^C NMR (CDCl_3_) δ 16.1 (d, ^3^*J*_CP_ = 6.8, OCH_2_*C*H_3_), 21.2 (CH_3_Ph), 54.1 (CH_2_Ph), 60.6 (d, ^2^*J*_CP_ = 5.2, CH_2_O), 64.0 (d, ^2^*J*_CP_ = 5.9, O*C*H_2_CH_3_), 123.3 (CH=), 128.3 (C_2_), 129.9 (C_3_), 131.5 (C_1_), 138.9 (C_4_), 143.7 (d, ^3^*J*_CP_ = 6.9, C=); [M+H]^+^_found_ = 340.1414, C_15_H_23_N_3_O_4_P requires 340.1426.

*[1-(2-Fluorobenzyl)-1H-1,2,3-triazol-4-yl]methyl diethyl phosphate* (**14c**): Yield: 54% (0.19 g), pale yellow oil; ^31^P NMR (CDCl_3_) δ –1.0; ^1^H NMR (CDCl_3_) δ 1.28 (t, ^3^*J*_HH_ = 7.1, 6H, OCH_2_C*H*_3_), 3.97–4.17 (m, 4H, OC*H*_2_CH_3_), 5.16 (d, ^3^*J*_HP_ = 9.3, 2H, CH_2_O), 5.59 (s, 2H, CH_2_Ph), 7.07–7.20 (m, 2H, C_3_H, C_5_H), 7.24–7.31 (m, 1H, C_6_H), 7.32–7.41 (m, 1H, C_4_H), 7.70 (s, 1H, CH); ^13^C NMR (CDCl_3_) δ 16.2 (d, ^3^*J*_CP_ = 6.8, OCH_2_*C*H_3_), 47.9 (d, ^3^*J*_CF_ = 4.3, CH_2_Ph), 60.6 (d, ^2^*J*_CP_ = 5.3, CH_2_O), 64.1 (d, ^2^*J*_CP_ = 5.9, O*C*H_2_CH_3_), 116.0 (d, ^2^*J*_CF_ = 21.1, C_3_), 121.8 (d, ^2^*J*_CF_ = 14.6, C_1_), 123.6 (CH=), 125.0 (d, *J*_CF_ = 3.7, C_5_), 130.8 (d, ^3^*J*_CF_ = 3.2, C_4_), 131.2 (d, ^3^*J*_CF_ = 8.3, C_6_), 143.9 (d, ^3^*J*_CP_ = 7.0, C=), 160.7 (d, ^1^*J*_CF_ = 248.0, C_2_); [M+H]^+^_found_ = 344.1164, C_14_H_20_N_3_O_4_FP requires 344.1175.

*[1-(3-Fluorobenzyl)-1H-1,2,3-triazol-4-yl]methyl diethyl phosphate* (**14d**): Yield: 56% (0.20 g), pale yellow oil; ^31^P NMR (CDCl_3_) δ –0.9; ^1^H NMR (CDCl_3_) δ 1.28 (t, ^3^*J*_HH_ = 7.1, 6H, OCH_2_C*H*_3_), 3.95–4.15 (m, 4H, OC*H*_2_CH_3_), 5.17 (d, ^3^*J*_HP_ = 9.5, 2H, CH_2_O), 5.53 (s, 2H, CH_2_Ph), 6.89–7.00 (m, 1H, C_4_H), 7.00–7.12 (m, 2H, C_2_H, C_6_H), 7.26–7.42 (m, 1H, C_5_H), 7.66 (s, 1H, CH); ^13^C NMR (CDCl_3_) δ 16.2 (d, ^3^*J*_CP_ = 6.8, OCH_2_*C*H_3_), 53.7 (d, *J*_CF_ = 1.9, CH_2_Ph), 60.6 (d, ^2^*J*_CP_ = 5.2, CH_2_O), 64.1 (d, ^2^*J*_CP_ = 6.0, O*C*H_2_CH_3_), 115.2 (d, ^2^*J*_CF_ = 22.3, C_4_), 116.1 (d, ^2^*J*_CF_ = 21.0, C_2_), 123.6 (CH=), 123.7 (d, *J*_CF_ = 3.1, C_6_), 131.0 (d, ^3^*J*_CF_ = 8.3, C_5_), 136.9 (d, ^3^*J*_CF_ = 7.4, C_1_), 144.2 (d, ^3^*J*_CP_ = 6.8, C=), 163.2 (d, ^1^*J*_CF_ = 248.1, C_3_); [M+H]^+^_found_ = 344.1164, C_14_H_20_N_3_O_4_FP requires 344.1175.

*[1-(4-Fluorobenzyl)-1H-1,2,3-triazol-4-yl])methyl diethyl phosphate* (**14e**): Yield: 61% (0.22 g), pale yellow oil; ^31^P NMR (CDCl_3_) δ –1.0; ^1^H NMR (CDCl_3_) δ 1.28 (t, ^3^*J*_HH_ = 7.1, 6H, OCH_2_C*H*_3_), 3.88–4.15 (m, 4H, OC*H*_2_CH_3_), 5.16 (d, ^3^*J*_HP_ = 9.5, 2H, CH_2_O), 5.50 (s, 2H, CH_2_Ph), 7.06 (t, *J*_HF_ = ^3^*J*_HH_ = 8.6, 2H, C_2_H), 7.28 (dd, ^3^*J*_HF_ = 8.0, ^3^*J*_HH_ = 5.8, 2H, C_3_H), 7.63 (s, 1H, CH); ^13^C NMR (CDCl_3_) δ 16.2 (d, ^3^*J*_CP_ = 6.8, OCH_2_*C*H_3_), 53.6 (CH_2_Ph), 60.6 (d, ^2^*J*_CP_ = 5.2, CH_2_O), 64.1 (d, ^2^*J*_CP_ = 6.0, O*C*H_2_CH_3_), 116.3 (d, ^2^*J*_CF_ = 21.8, C_3_), 123.4 (CH=), 130.2 (d, ^3^*J*_CF_ = 8.4, C_2_), 130.4 (d, *J*_CF_ = 3.3, C_1_), 144.0 (d, ^3^*J*_CP_ = 6.9, C=), 163.1 (d, ^1^*J*_CF_ = 248.3, C_4_); [M+H]^+^_found_ = 344.1163, C_14_H_20_N_3_O_4_FP requires 344.1175.

*[1-(4-Trifluoromethyl)benzyl)-1H-1,2,3-triazol-4-yl]methyl diethyl phosphate* (**14f**): Yield: 68% (0.27 g), pale yellow oil; ^31^P NMR (CDCl_3_) δ –0.9; ^1^H NMR (CDCl_3_) δ 1.23 (t, ^3^*J*_HH_ = 7.1, 6H, OCH_2_C*H*_3_), 3.92–4.12 (m, 4H, OC*H*_2_CH_3_), 5.13 (d, ^3^*J*_HP_ = 9.4, 2H, CH_2_O), 5.57 (s, 2H, CH_2_Ph), 7.36 (d, ^3^*J*_HH_ = 8.0, 2H, C_2_H), 7.59 (d, ^3^*J*_HH_ = 8.0, 2H, C_3_H), 7.68 (s, 1H, CH); ^13^C NMR (CDCl_3_) δ 16.1 (d, ^3^*J*_CP_ = 6.7, OCH_2_*C*H_3_), 53.6 (CH_2_Ph), 60.5 (d, ^2^*J*_CP_ = 5.2, CH_2_O), 64.0 (d, ^2^*J*_CP_ = 5.9, O*C*H_2_CH_3_), 123.7 (CH=), 123.8 (q, ^1^*J*_CF_ = 272.4, CF_3_), 126.1 (q, ^3^*J*_CF_ = 3.8, C_3_), 128.4 (C_2_), 131.1 (q, ^2^*J*_CF_ =32.8, C_4_), 138.6 (C_1_), 144.1 (d, ^3^*J*_CP_ = 6.8, C=); [M+H]^+^_found_ = 394.1157, C_15_H_20_N_3_O_4_F_3_P requires 394.1143.

*(1-Octyl-1H-1,2,3-triazol-4-yl)methyl diethyl phosphate* (**14g**): Yield: 60% (0.21 g), pale yellow oil; ^31^P NMR (CDCl_3_) δ –1.3; ^1^H NMR (CDCl_3_) δ 0.88 (t, ^3^*J*_HH_ = 6.7, 3H, CH_3_), 1.16–1.43 (m, 16H, CH_2_CH_2_(C*H*_2_)_5_CH_3_, OCH_2_C*H*_3_), 1.81–2.01 (m, 2H, CH_2_C*H*_2_(CH_2_)_5_CH_3_), 4.01–4.22 (m, 4H, OC*H*_2_CH_3_), 4.35 (t, 2H, ^3^*J*_HH_ = 7.3, C*H*_2_CH_2_(CH_2_)_5_CH_3_), 5.19 (d, ^3^*J*_HP_ = 9.4, 2H, CH_2_O), 7.68 (s, 1H, CH); ^13^C NMR (CDCl_3_) δ 14.2 (CH_3_), 16.2 (d, ^3^*J*_CP_ = 6.8, OCH_2_*C*H_3_), 22.7 (*C*H_2_CH_3_), 26.6 (*C*H_2_CH_2_CH_3_), 29.1 (*C*H_2_(CH_2_)_2_CH_3_), 29.2 (*C*H_2_(CH_2_)_3_CH_3_), 30.4 (*C*H_2_(CH_2_)_4_CH_3_), 31.8 (*C*H_2_(CH_2_)_5_CH_3_), 50.6 (*C*H_2_(CH_2_)_6_CH_3_), 60.7 (d, ^2^*J*_CP_ = 5.3, CH_2_O), 64.1 (d, ^2^*J*_CP_ = 6.0, O*C*H_2_CH_3_), 123.4 (CH=), 143.4 (d, ^3^*J*_CP_ = 6.7, C=); [M+H]^+^_found_ = 348.2043, C_15_H_31_N_3_O_4_P requires 348.2052.

*(1-Iso-octyl-1H-1,2,3-triazol-4-yl)methyl diethyl phosphate* (**14h**): Yield: 54% (0.19 g), pale yellow oil; ^31^P NMR (CDCl_3_) δ –1.2; ^1^H NMR (CDCl_3_) δ 0.91 (t, ^3^*J*_HH_ = 7.4, 6H, CH_3_), 1.12–1.42 (m, 14H, CH(C*H*_2_)_3_CH_3_, CHC*H*_2_CH_3_, OCH_2_C*H*_3_), 1.80–1.95 (m, 1H, NCH_2_C*H*), 3.98–4.15 (m, 4H, OC*H*_2_CH_3_), 4.27 (d, 2H, ^3^*J*_HH_ = 6.8, NC*H*_2_CH), 5.19 (d, ^3^*J*_HP_ = 9.4, 2H, CH_2_O), 7.66 (s, 1H, CH); ^13^C NMR (CDCl_3_) δ 10.6 (NCH_2_CHCH_2_*C*H_3_), 14.1 (CH_3_), 16.2 (d, ^3^*J*_CP_ = 6.7, OCH_2_*C*H_3_), 23.0 (*C*H_2_CH_3_), 23.8 (NCH_2_CH*C*H_2_CH_3_), 28.6 (*C*H_2_CH_2_CH_3_), 30.5 (*C*H_2_(CH_2_)_2_CH_3_), 40.5 (NCH_2_*C*HCH_2_CH_3_), 53.7 (N*C*H_2_CHCH_2_CH_3_), 60.7 (d, ^2^*J*_CP_ = 5.3, CH_2_O), 64.1 (d, ^2^*J*_CP_ = 6.0, O*C*H_2_CH_3_), 124.0 (CH=), 143.3 (d, ^3^*J*_CP_ = 6.6, C=); [M+H]^+^_found_ = 348.2043, C_15_H_31_N_3_O_4_P requires 348.2052.

*(1-Cyclohexyl-1H-1,2,3-triazol-4-yl)methyl diethyl phosphate* (**14i**): Yield: 51% (0.16 g), pale yellow oil; ^31^P NMR (CDCl_3_) δ –1.3; ^1^H NMR (CDCl_3_) δ 1.31 (t, ^3^*J*_HH_ = 6.9, 6H, OCH_2_C*H*_3_), 1.17–1.55 (m, 4H, C_3_H_ax_, C_4_H_ax_, C_4_H_eq_), 1.67–1.82 (m, 2H, C_3_H_eq_), 1.88–1.98 (m, 2H, C_2_H_ax_), 2.15–2.26 (m, 2H, C_2_H_eq_), 4.02–4.16 (m, 4H, OC*H*_2_CH_3_), 4.38–4.52 (m, 1H, C_1_H), 5.18 (d, ^3^*J*_HP_ = 9.2, 2H, CH_2_O), 7.70 (s, 1H, CH); ^13^C NMR (CDCl_3_) δ 16.2 (d, ^3^*J*_CP_ = 6.8, OCH_2_*C*H_3_), 25.2 (C_4_), 25.3 (C_3_), 33.7 (C_2_), 60.3 (C_1_), 60.8 (d, ^2^*J*_CP_ = 5.2, CH_2_O), 64.0 (d, ^2^*J*_CP_ = 5.9, O*C*H_2_CH_3_), 121.3 (CH=), 142.9 (d, ^3^*J*_CP_ = 6.8, C=); [M+H]^+^_found_ = 318.1575, C_13_H_25_N_3_O_4_P requires 318.1583.

*(1-Phenyl-1H-1,2,3-triazol-4-yl)methyl diethyl phosphate* (**14j**): Yield: 73% (0.23 g), pale yellow oil; ^31^P NMR (CDCl_3_) δ –1.0; ^1^H NMR (CDCl_3_) δ 1.33 (t, ^3^*J*_HH_ = 7.1, 6H, OCH_2_C*H*_3_), 3.99–4.20 (m, 4H, OC*H*_2_CH_3_), 5.28 (d, ^3^*J*_HP_ = 9.2, 2H, CH_2_O), 7.37–7.61 (m, 9H, C_3_H, C_4_H), 7.74 (d, ^3^*J*_HH_ = 7.8, 2H, C_2_H), 8.16 (s, 1H, CH); ^13^C NMR (CDCl_3_) δ 16.2 (d, ^3^*J*_CP_ = 6.8, OCH_2_*C*H_3_), 60.5 (d, ^2^*J*_CP_ = 5.1, CH_2_O), 64.2 (d, ^2^*J*_CP_ = 6.0, O*C*H_2_CH_3_), 120.7 (C_2_), 121.8 (CH=), 129.1 (C_4_), 129.9 (C_3_), 137.0 (C_1_), 144.1 (d, ^3^*J*_CP_ = 7.0, C=); [M+H]^+^_found_ = 312.1104, C_13_H_19_N_3_O_4_P requires 312.1113.

## 4. Conclusions

In summary, we have developed a facile, efficient method for the synthesis of new (1-alkyl/aryl-1*H*-1,2,3-triazol-4-yl)methyl phosphinates or (1-alkyl/aryl-1*H*-1,2,3-triazol-4-yl)methyl diethyl phosphates by the copper(I)-catalyzed azide-alkyne cycloaddition of organic azides and prop-2-ynyl phosphinate or diethyl prop-2-ynyl phosphate. This method, which has the advantages of simple operation and mild reaction conditions, is a novel approach for the synthesis of the target products. Altogether, 20 new derivatives were synthesized and fully characterized.

## Figures and Tables

**Table 1:**
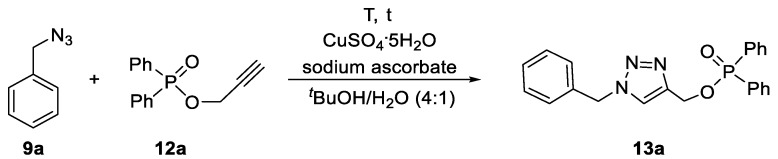
Optimization of the reaction of benzyl azide (**9a**) and propynyl diphenylphosphinate (**11a**).

Entry	Mode of Heating	T [°C]	t [min]	Catalyst [mol%]		Conversion [%] ^a^	Yield [%] ^b^
				CuSO_4_·5H_2_O	Sodium Ascorbate		
1	–	25	60	5	30	100	84
2	–	25	60	5	10	78	–
3	–	25	180	5	10	100	89
4	Δ	60	5	5	10	93	–
5	MW	60	5	5	10	92	–
6	Δ	60	10	5	10	100	89
7	Δ	60	10	3	5	100	91
8	Δ	60	10	2	5	90	–

^a^ Based on ^31^P NMR (phosphorus-31 nuclear magnetic resonance); ^b^ Isolated yield; MW (microwave).

**Table 2:**
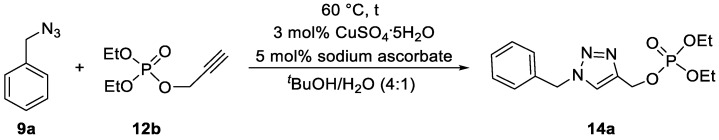
Reaction of benzyl azide (**9a**) and diethyl prop-2-ynyl phosphate (**12b**).

Entry	t (min)	Conversion (%) ^a^	Yield (%) ^b^
1	10	40	–
2	20	59	–
3	30	98 ^c^	75

^a^ Based on ^31^P NMR. ^b^ Isolated yield. ^c^ No change for longer reaction time.

**Table 3:** MS Data for benzyl azides (**9a–f**).

Compound	Yield	[M+H]^+^_found_	[M+H]^+^_requires_
Benzyl azide (**9a**) [26]	93% (1.24 g)	134.0725	134.0718
4-Methylbenzyl azide (**9b**) [28]	80% (1.18 g)	148.0880	148.0874
2-Fluorobenzyl azide (**9c**) [29]	68% (1.02 g)	152.0632	152.0624
3-Fluorobenzyl azide (**9d**) [30]	76% (1.14 g)	152.0633	152.0624
4-Fluorobenzyl azide (**9e**) [28]	83% (1.25 g)	152.0632	152.0624
4-(Trifluoromethyl)benzyl azide (**9f**) [31]	86% (1.72 g)	202.0601	202.0592

**Table 4:** MS Data for alkyl azides (**9g–i**).

Compound	Yield	[M+H]^+^_found_	[M+H]^+^_requires_
Octyl azide (**9g**) [32]	67% (1.04 g)	156.1514	156.1501
Iso-octyl azide (**9h**) [33]	55% (0.85 g)	156.1514	156.1501
Cyclohexyl azide (**9i**) [27]	52% (0.65 g)	126.1038	126.1031

**Table 5:** ^31^P NMR and MS Data for prop-2-ynyl diphenylphosphinate (**12a**) and diethyl prop-2-ynyl phosphate (**12b**).

Compound	Yield	δ_P_ in CDCl_3_	δ_P_ [lit.] in CDCl_3_	[M+H]^+^_found_	[M+H]^+^_requires_
**12a**	88% (2.25 g)	34.3	34.2 [25]	257.0735	257.0731
**12b**	72% (1.38 g)	−0.4	−0.7 [34]	193.0639	193.0629

## Supplementary Materials

Supplementary data associated with this article are available online. Copies of ^31^P, ^1^H, and ^13^C NMR spectra for all compounds synthesized are presented.
