# Supplementary material for: Synthesis of (1,2,3-triazol-4-yl)methyl Phosphinates and (1,2,3-Triazol-4-yl)methyl Phosphates by Copper-Catalyzed Azide-Alkyne Cycloaddition
Source: Molecules. 2019 May 31;24(11):2085. doi: 10.3390/molecules24112085 (PMC6600419; doi:10.3390/molecules24112085)
Supplement: Supplementary file 1 [file molecules-24-02085-s001.pdf]

Supplementary Material

for

Synthesis of (1,2,3-triazol-4-yl)methyl phosphinates and (1,2,3-triazol-4-yl)methyl phosphates by copper-catalyzed azide-alkyne cycloaddition

Anna Tripolszky [1], Krisztina Németh [2], Pál Tamás Szabó [2] and Erika Bálint [1]*

Address:

[1] Department of Organic Chemistry and Technology, Budapest University of Technology and Economics, 1521 Budapest, Hungary
[2] MS Metabolomics Laboratory, Instrumentation Center, Research Centre for Natural Sciences, Hungarian Academy of Sciences, Magyar tudósok krt. 2., H-1117 Budapest, Hungary

E-mail:

Erika Bálint* - ebalint@mail.bme.hu

* Corresponding author

Table of contents

^{31}P NMR, ^{1}H NMR and ^{13}C NMR spectra S2–S31

^{31}P NMR, ^{1}H NMR and ^{13}C NMR spectra of compounds

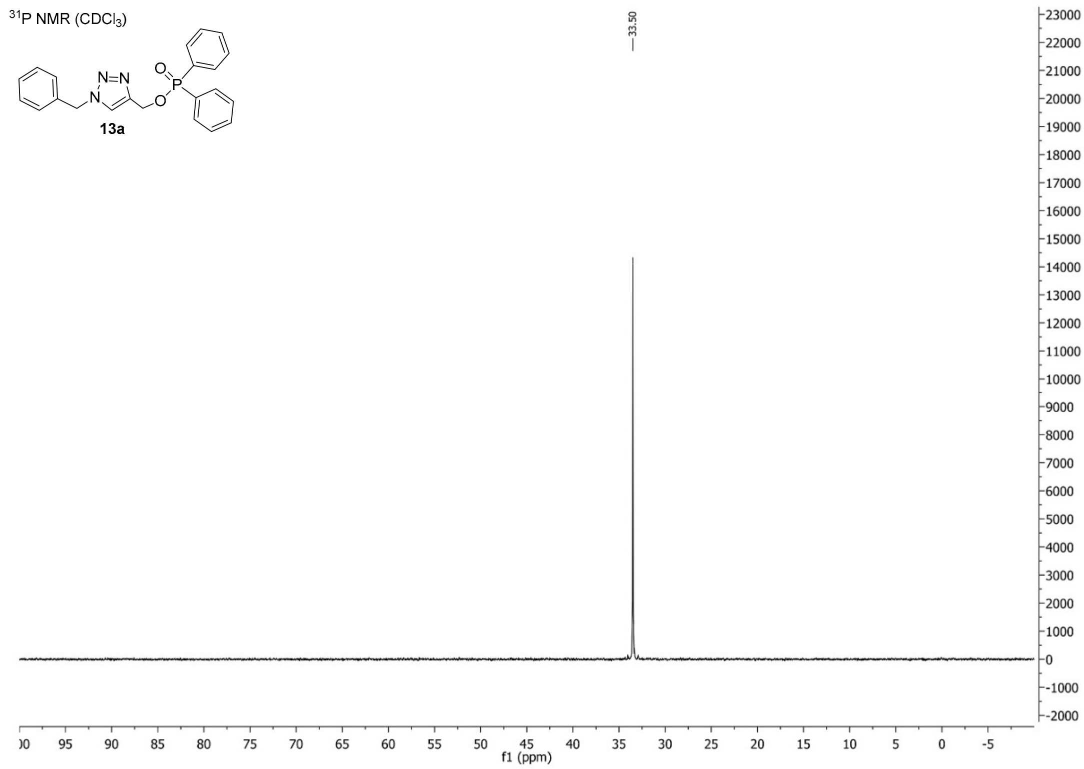

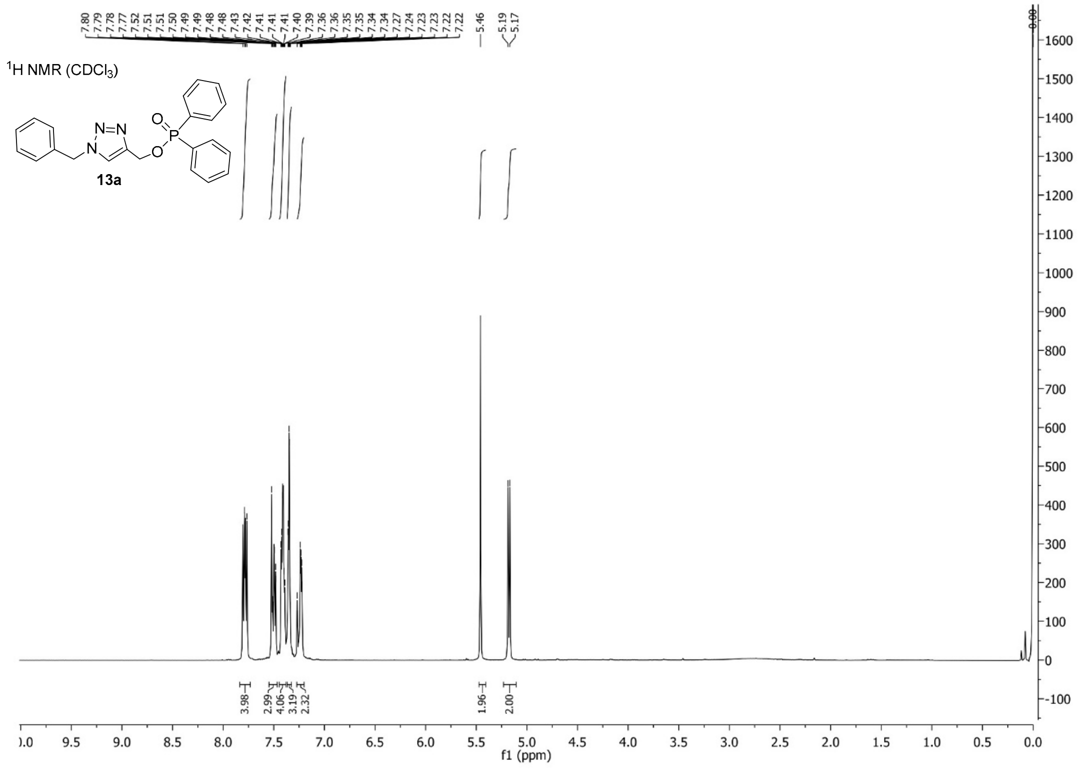

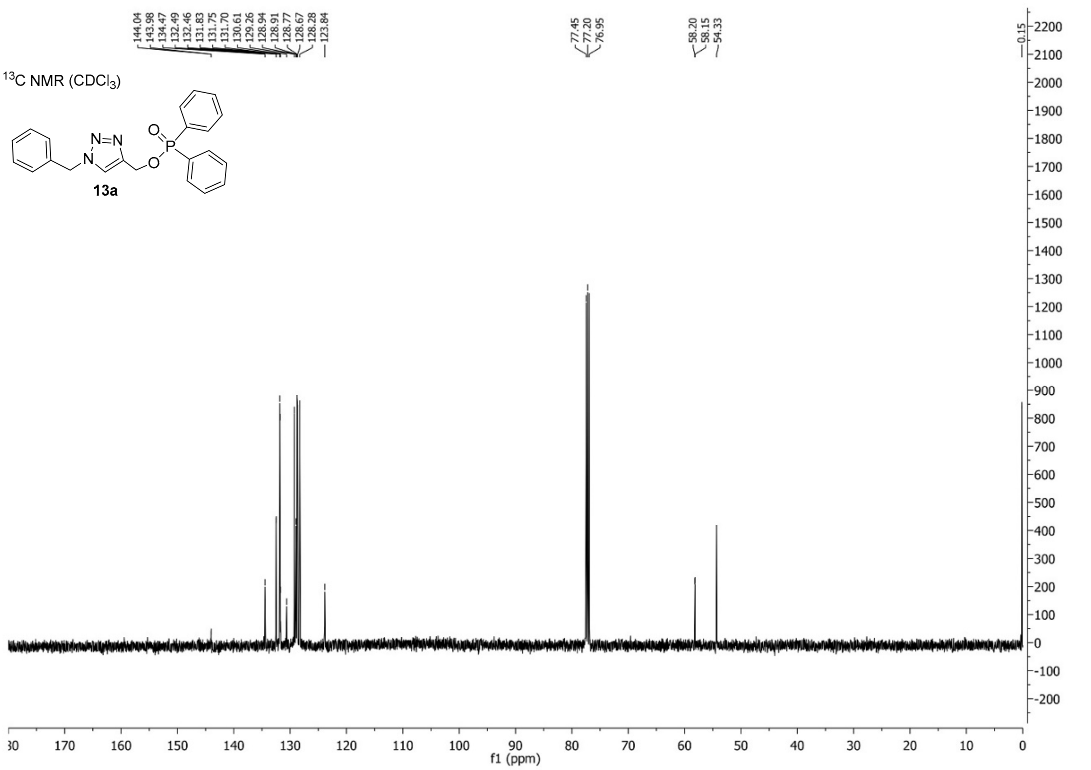

13C NMR (CDCl3)
13a
144.04
143.98
134.47
132.49
132.46
131.83
131.75
131.70
130.61
129.26
128.94
128.91
128.77
128.67
128.28
123.84
77.45
77.20
76.95
58.20
58.15
54.33
0.15
f1 (ppm)

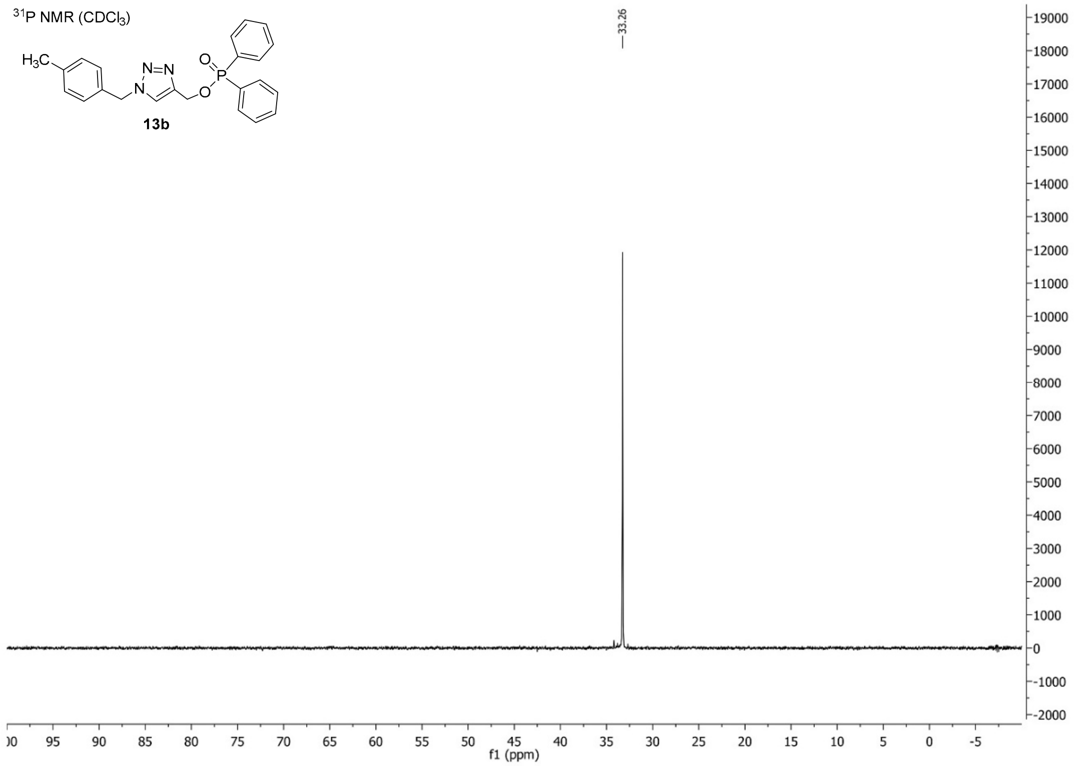

31P NMR (CDCl3)
13b
33.26
f1 (ppm)

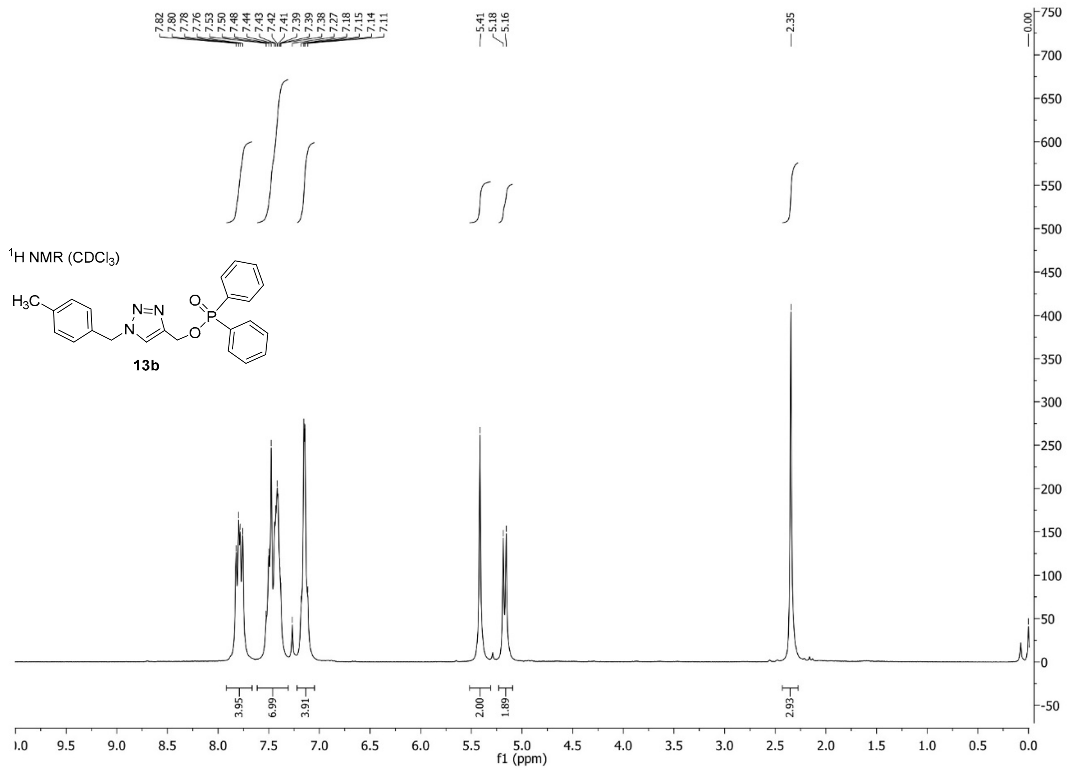
7.82 7.80 7.78 7.76 7.53 7.50 7.48 7.44 7.43 7.42 7.41 7.39 7.39 7.38 7.27 7.18 7.15 7.14 7.11
5.41 5.18 5.16
2.35
0.00
1H NMR (CDCl3)
H3C
N=N
N
O
O
P
13b
3.95
6.99
3.91
2.00
1.89
2.93
f1 (ppm)

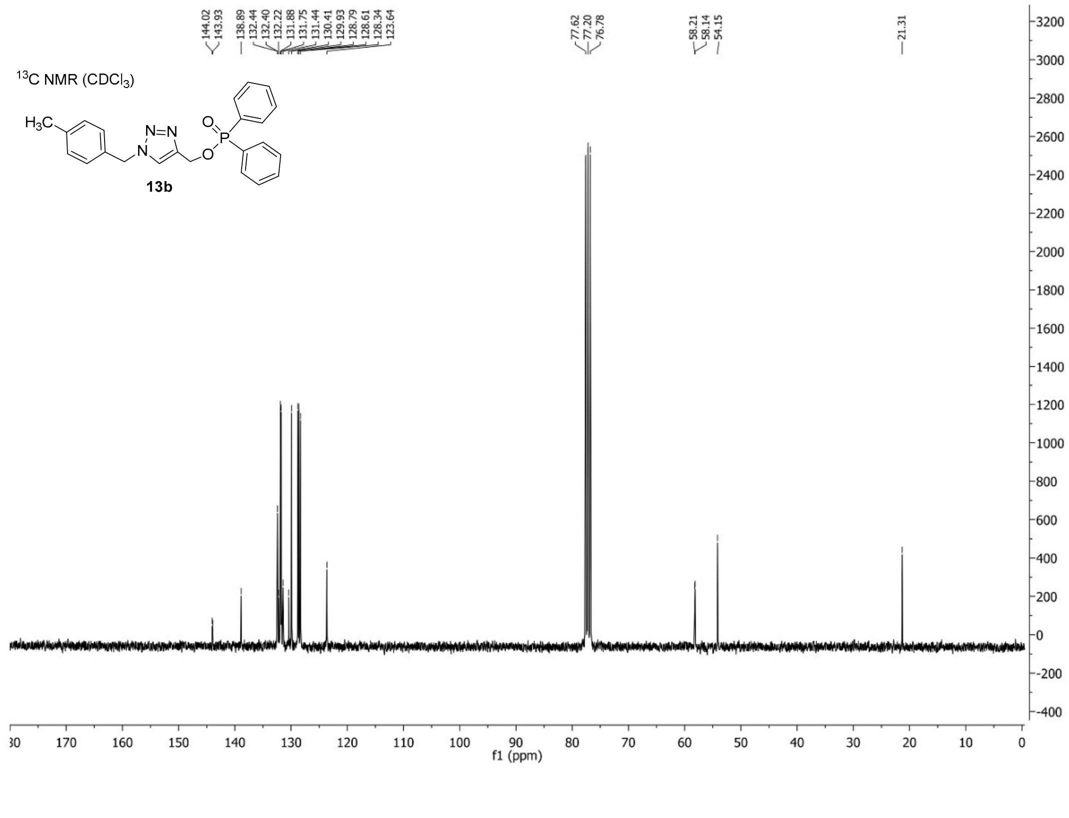
144.02 143.93 138.89 132.44 132.40 132.22 131.88 131.75 131.44 130.41 129.93 128.79 128.61 128.34 123.64
77.62 77.20 76.78
58.21 58.14 54.15
21.31
13C NMR (CDCl3)
H3C
N=N
N
O
O
P
13b
f1 (ppm)

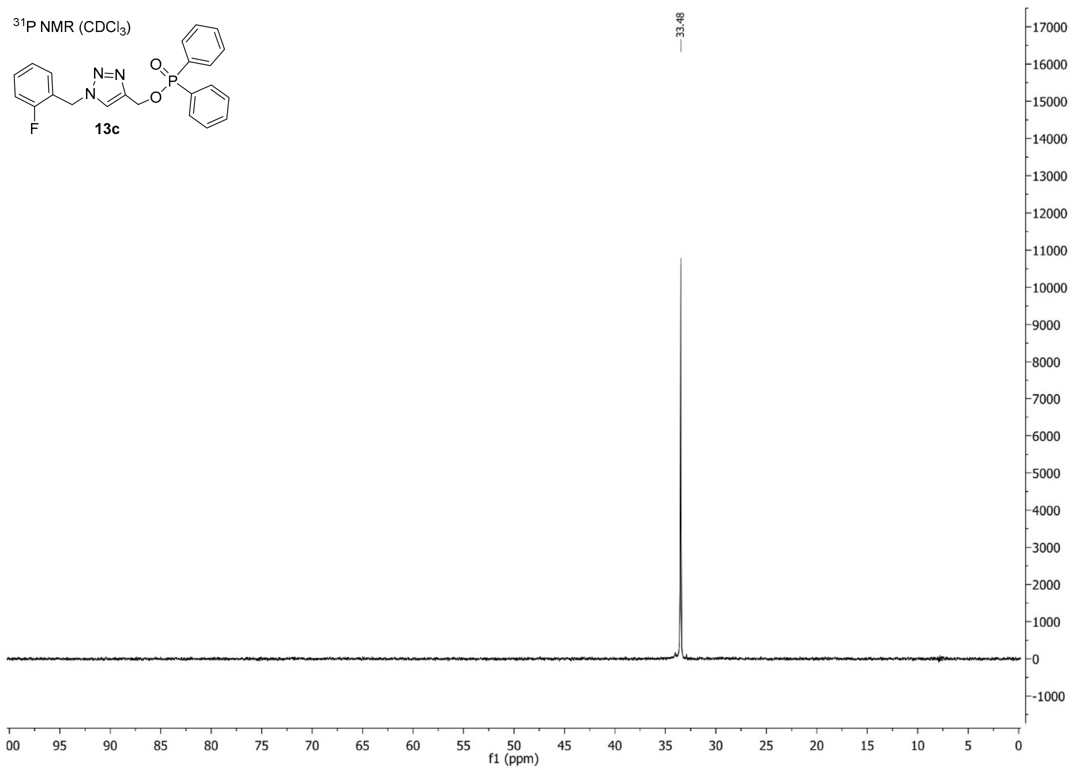
31P NMR (CDCl3)
13c
33.48
f1 (ppm)

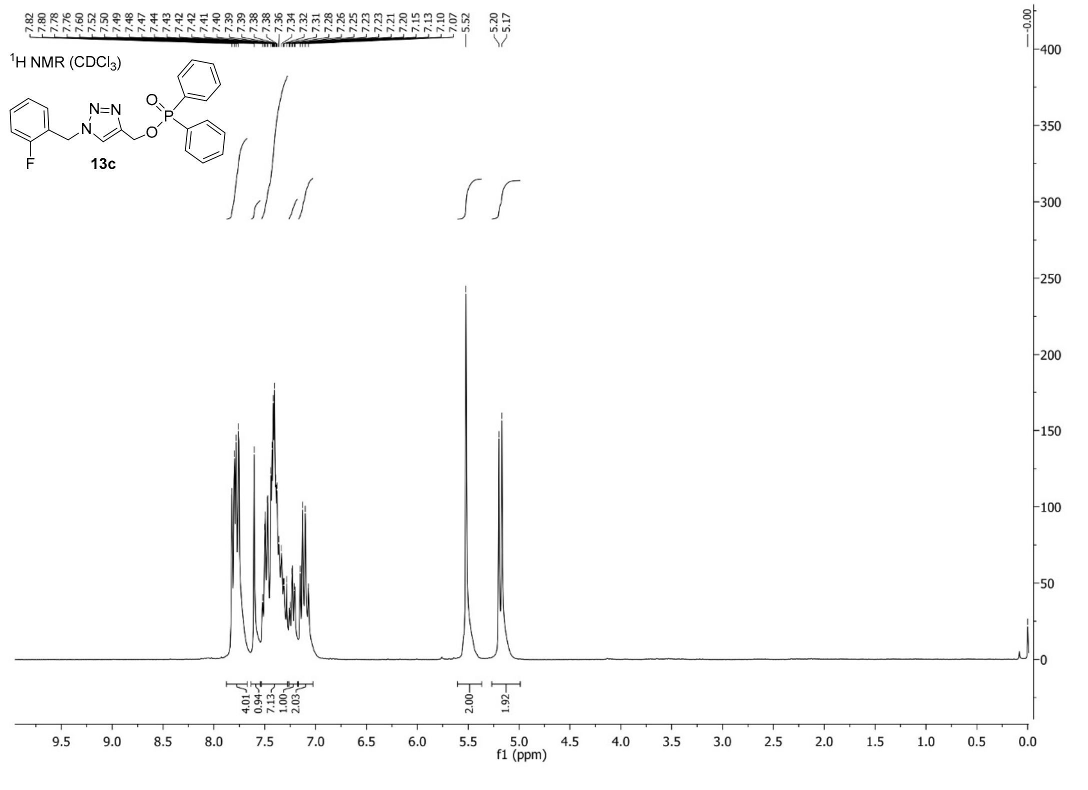
7.82 7.80 7.78 7.76 7.60 7.52 7.50 7.49 7.48 7.47 7.44 7.43 7.42 7.42 7.41 7.40 7.39 7.39 7.38 7.38 7.36 7.34 7.32 7.31 7.28 7.26 7.25 7.23 7.23 7.21 7.20 7.15 7.13 7.10 7.07
5.52
5.20 5.17
0.00
1H NMR (CDCl3)
13c
4.01 0.94 7.13 1.00 2.03
2.00
1.92
f1 (ppm)

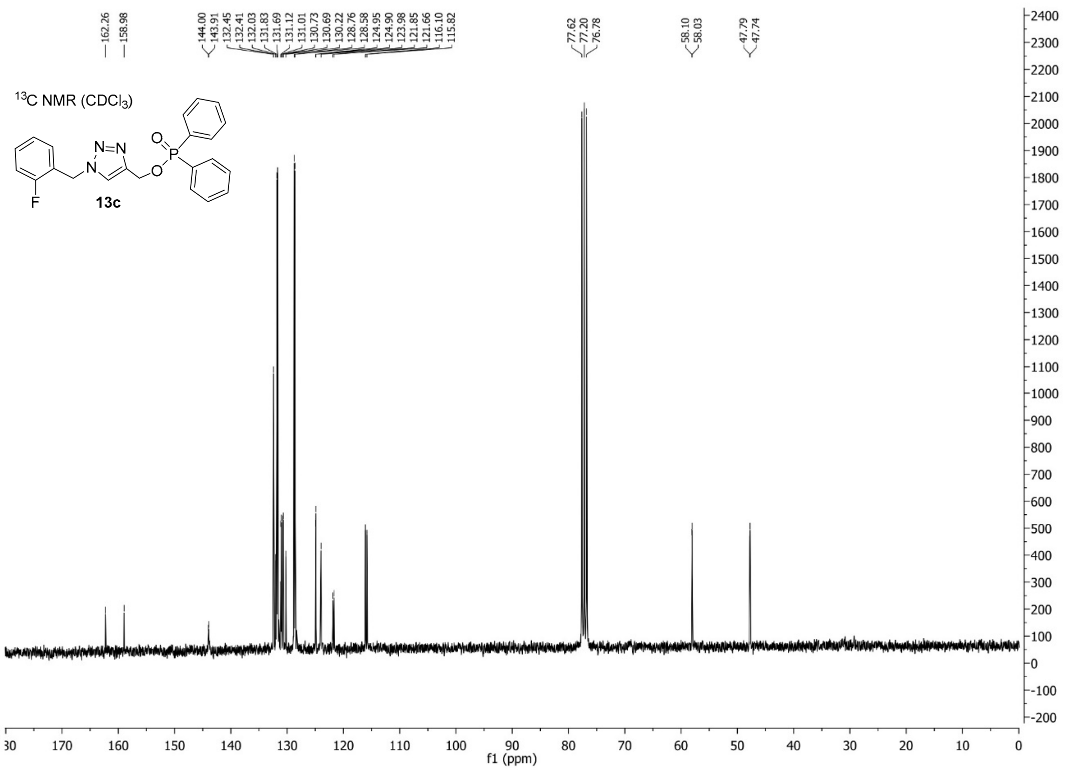

162.26
158.98
144.00
143.91
132.45
132.41
132.03
131.83
131.69
131.12
131.01
130.73
130.69
130.22
128.76
128.58
124.95
124.90
123.98
121.85
121.66
116.10
115.82
77.62
77.20
76.78
58.10
58.03
47.79
47.74
13C NMR (CDCl3)
13c
f1 (ppm)

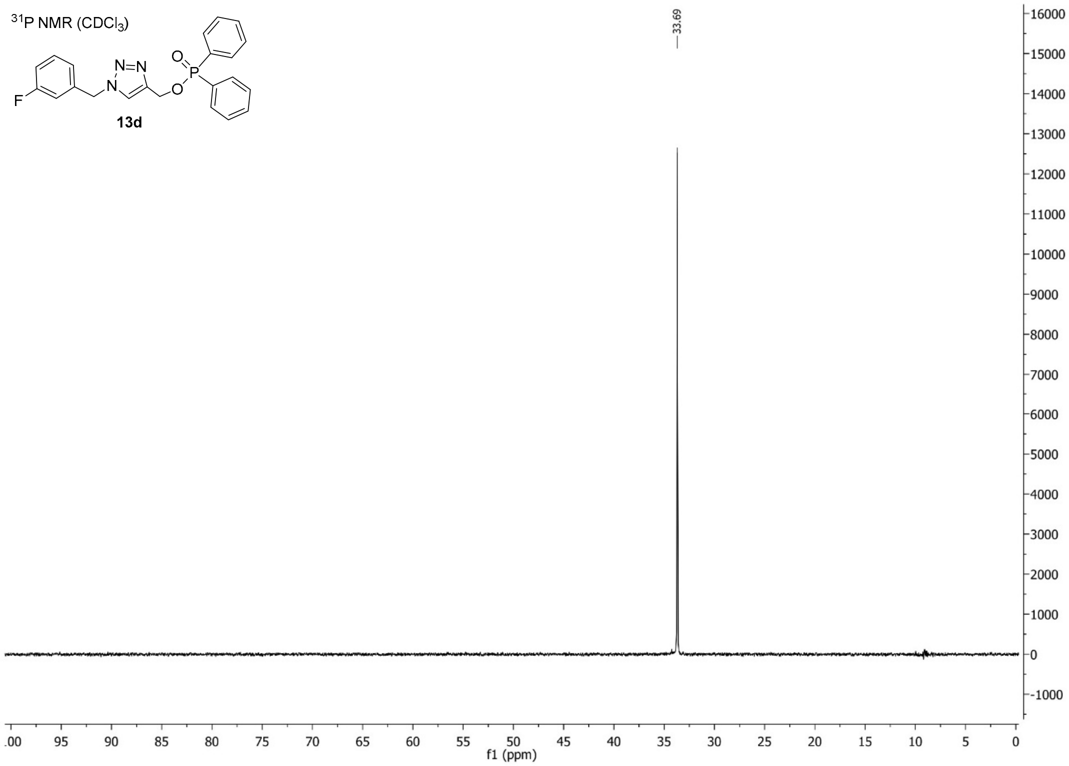

31P NMR (CDCl3)
13d
33.69
f1 (ppm)

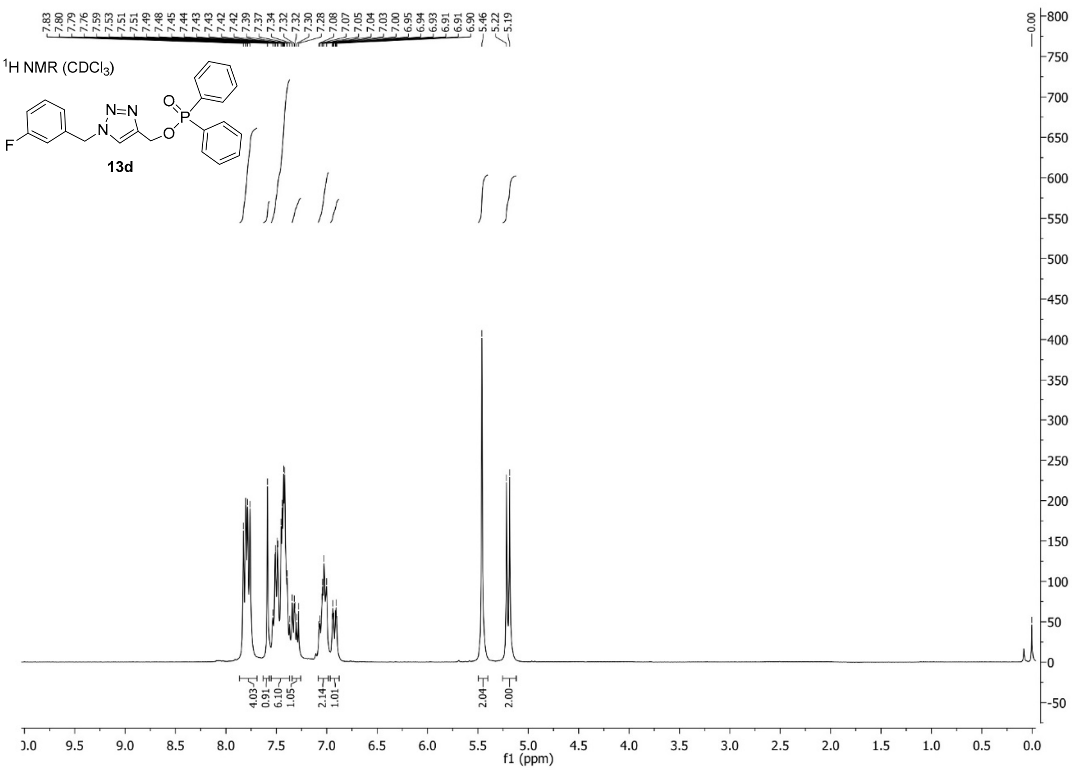
1H NMR (CDCl3)
13d
F
N
N=N
O
P
O
7.83
7.80
7.79
7.76
7.59
7.53
7.51
7.51
7.49
7.48
7.45
7.44
7.43
7.43
7.42
7.42
7.39
7.37
7.34
7.32
7.32
7.30
7.28
7.08
7.07
7.05
7.04
7.03
7.00
6.95
6.94
6.93
6.91
6.91
6.90
5.46
5.22
5.19
0.00
4.03
0.91
6.10
1.05
2.14
1.01
2.04
2.00
f1 (ppm)

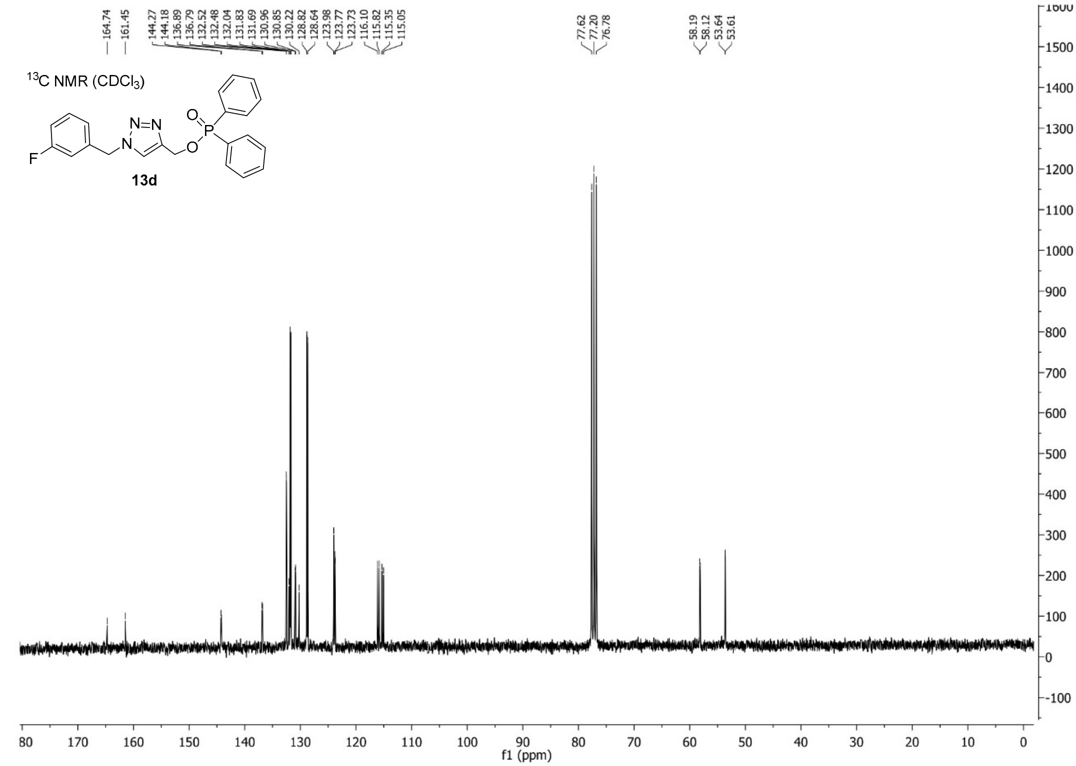
13C NMR (CDCl3)
13d
F
N
N=N
O
P
O
164.74
161.45
144.27
144.18
136.89
136.79
132.52
132.48
132.04
131.83
131.69
130.96
130.85
130.22
128.82
128.64
123.98
123.77
123.73
116.10
115.82
115.35
115.05
77.62
77.20
76.78
58.19
58.12
53.64
53.61
f1 (ppm)

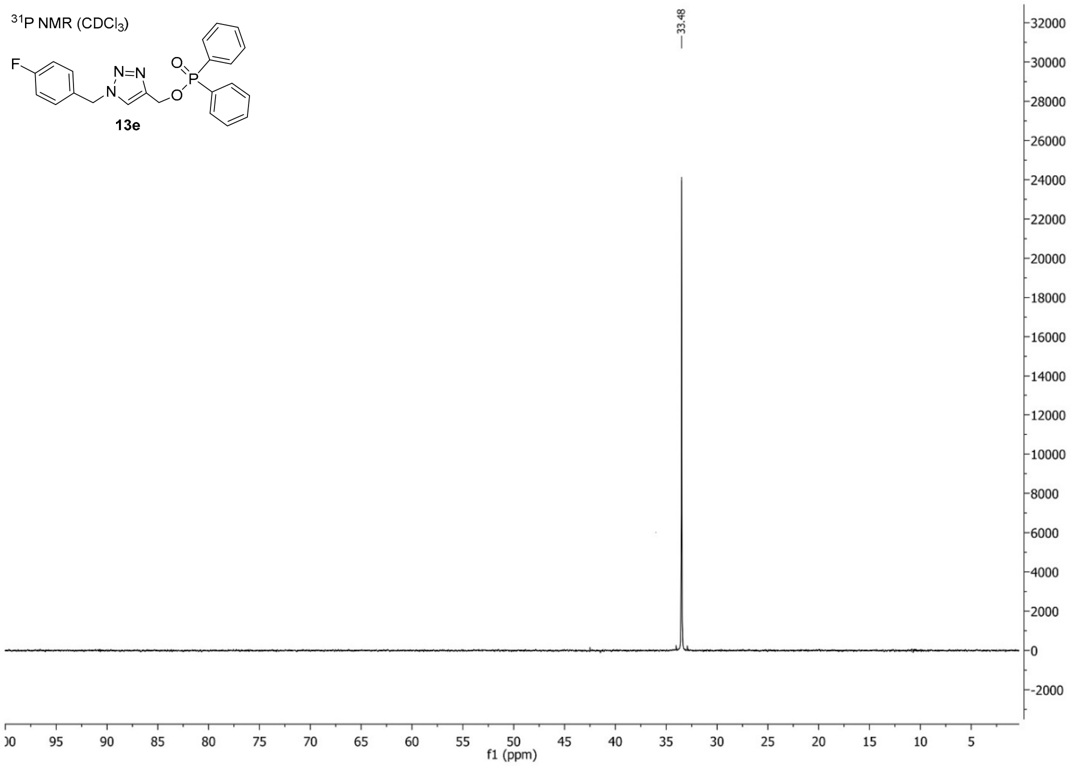
31P NMR (CDCl3)
13e
33.48
f1 (ppm)

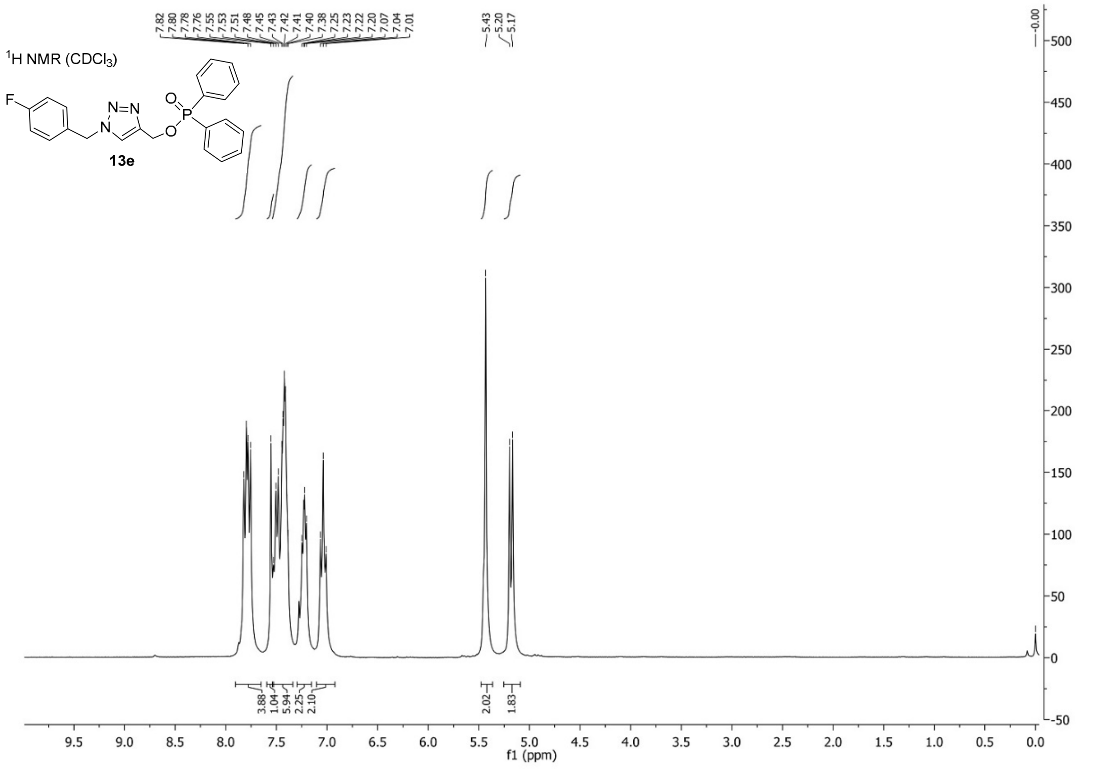
1H NMR (CDCl3)
13e
7.82 7.80 7.78 7.76 7.55 7.53 7.51 7.48 7.45 7.43 7.42 7.41 7.40 7.38 7.25 7.23 7.22 7.20 7.07 7.04 7.01
5.43 5.20 5.17
0.00
3.88 1.04 5.94 2.25 2.10
2.02 1.83
f1 (ppm)

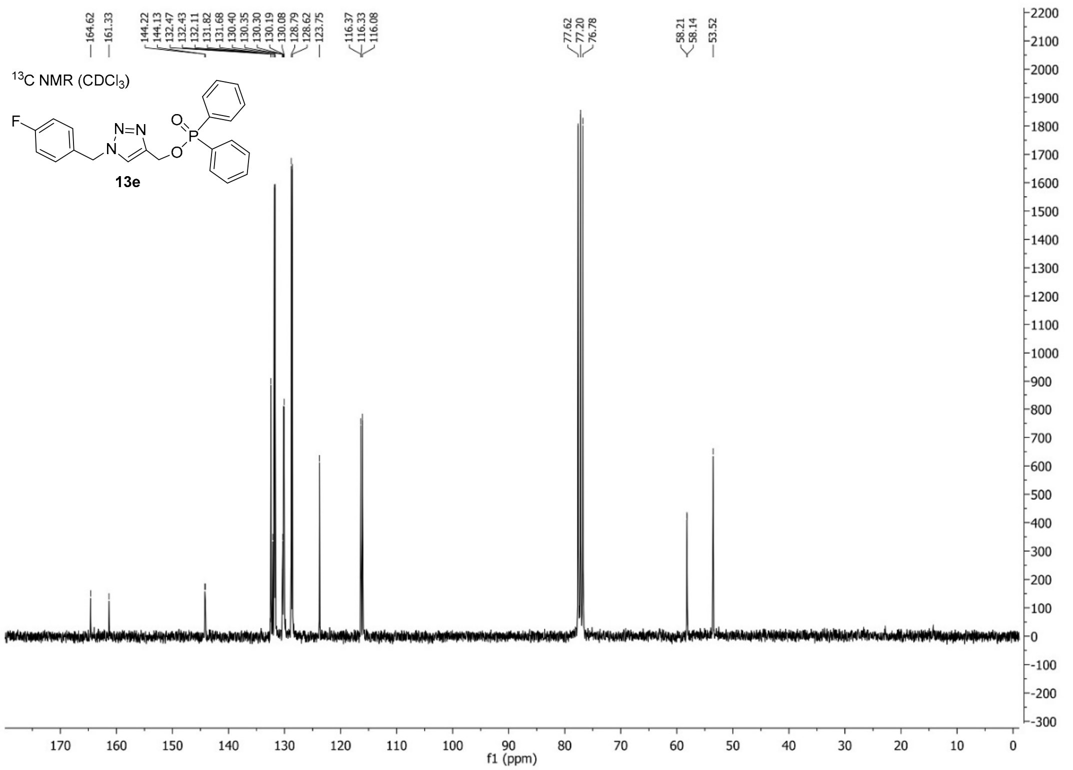

164.62
161.33
144.22
144.13
132.47
132.43
132.11
131.82
131.68
130.40
130.35
130.30
130.19
130.08
128.79
128.62
123.75
116.37
116.33
116.08
77.62
77.20
76.78
58.21
58.14
53.52
13C NMR (CDCl3)
13e
f1 (ppm)

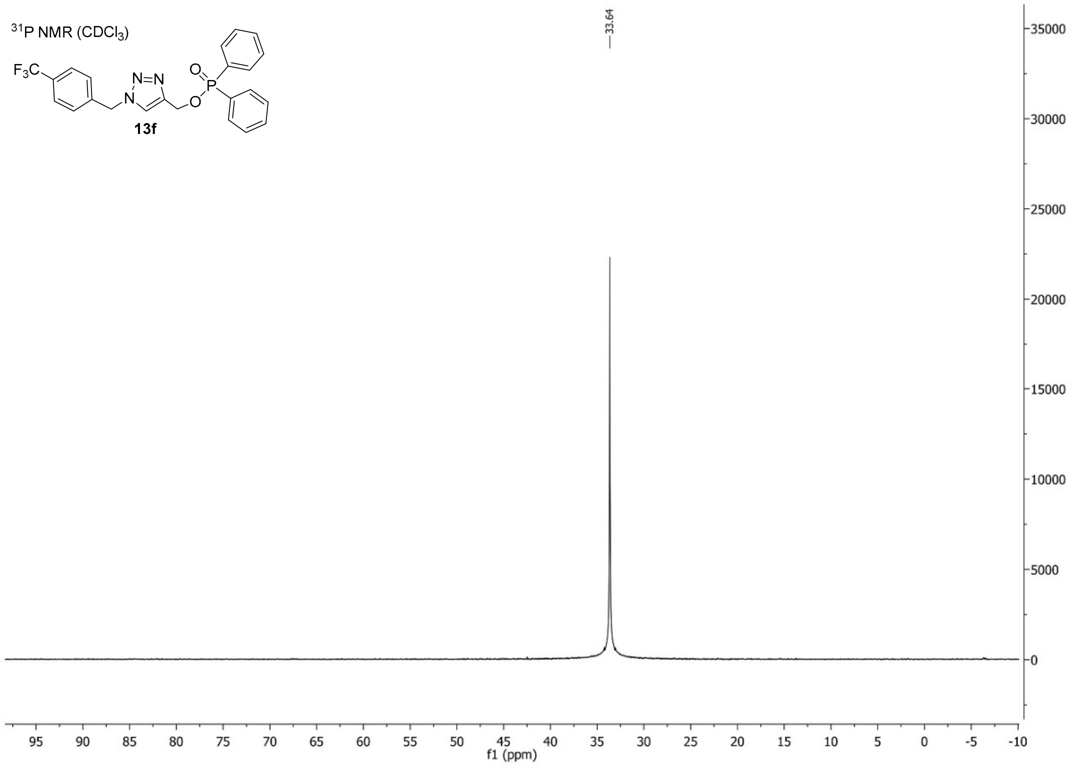

33.64
31P NMR (CDCl3)
13f
f1 (ppm)

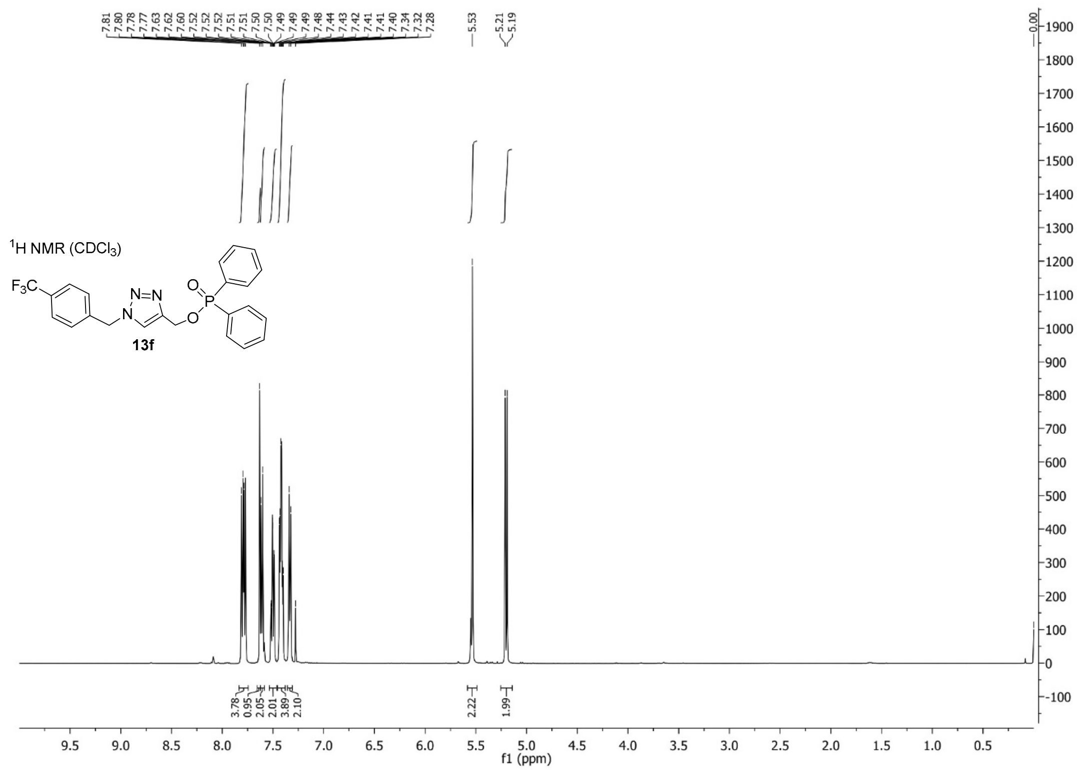
7.81
7.80
7.78
7.77
7.63
7.62
7.60
7.52
7.52
7.52
7.51
7.51
7.50
7.49
7.49
7.49
7.48
7.44
7.43
7.42
7.41
7.41
7.40
7.34
7.32
7.28
5.53
5.21
5.19
0.00
^{1}H NMR (CDCl$_3$)
F$_3$C
N
N=N
O
P
O
13f
3.78
0.95
2.05
2.01
3.89
2.10
2.22
1.99
f1 (ppm)

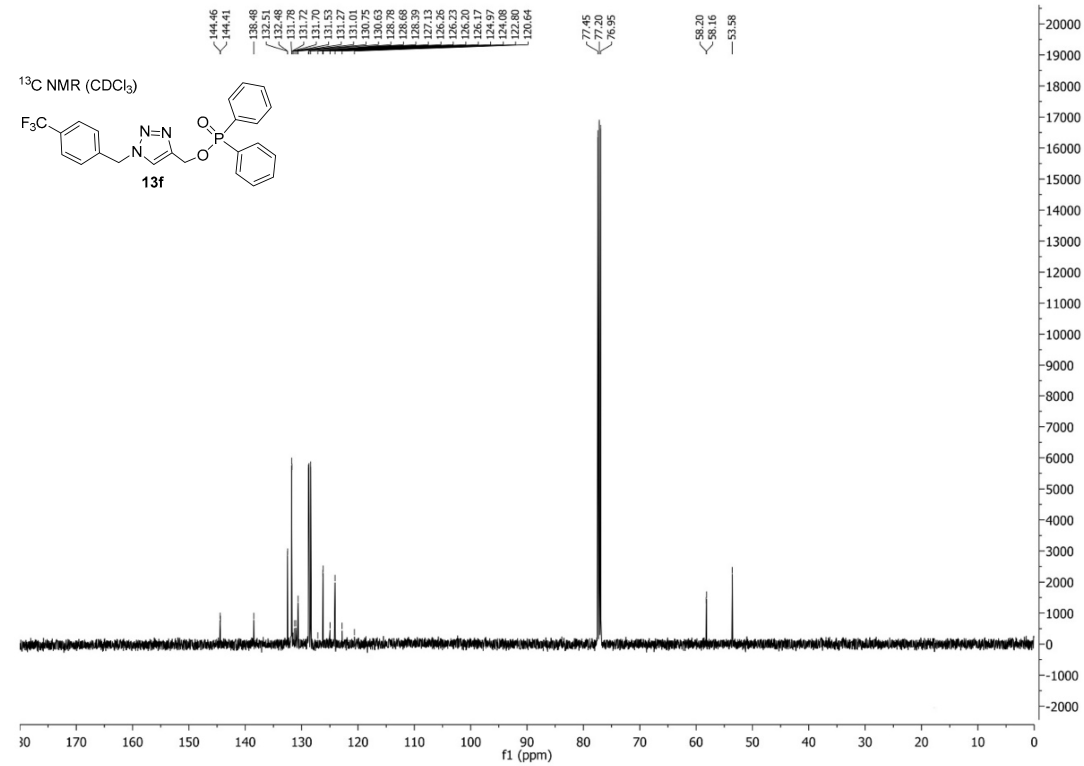
144.46
144.41
138.48
132.51
132.48
131.78
131.72
131.70
131.53
131.27
131.01
130.75
130.63
128.78
128.68
128.39
127.13
126.26
126.23
126.20
126.17
124.97
124.08
122.80
120.64
77.45
77.20
76.95
58.20
58.16
53.58
^{13}C NMR (CDCl$_3$)
F$_3$C
N
N=N
O
P
O
13f
f1 (ppm)

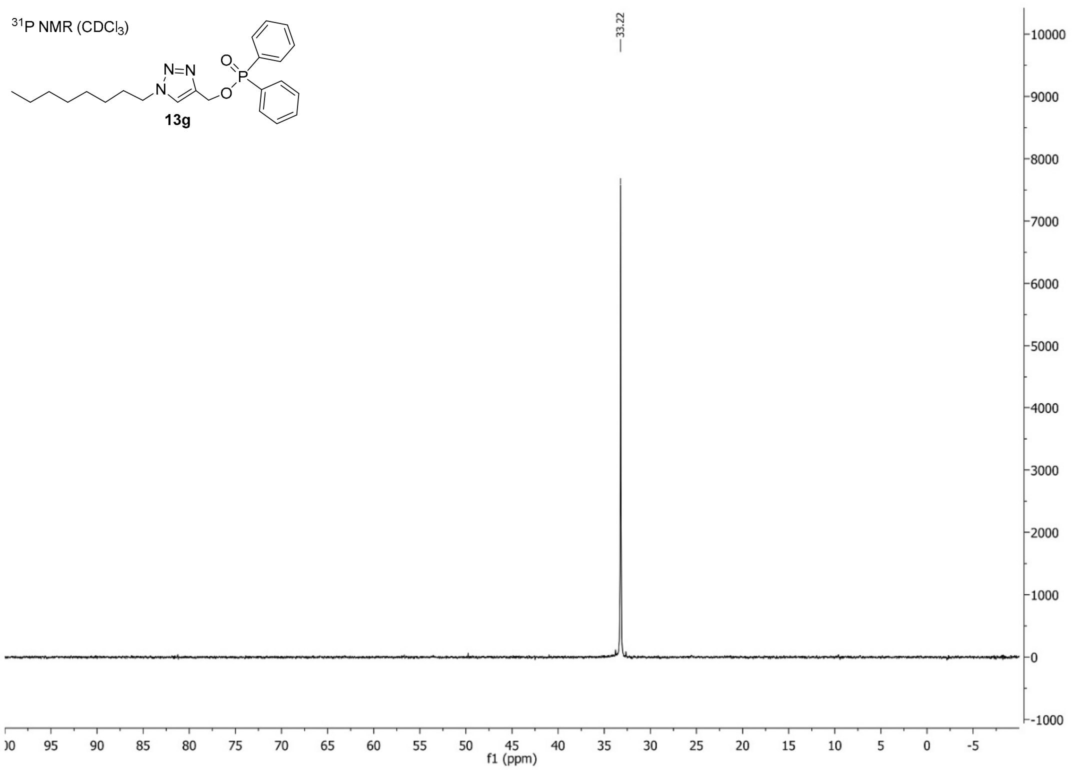

31P NMR (CDCl3)
13g
33.22
f1 (ppm)

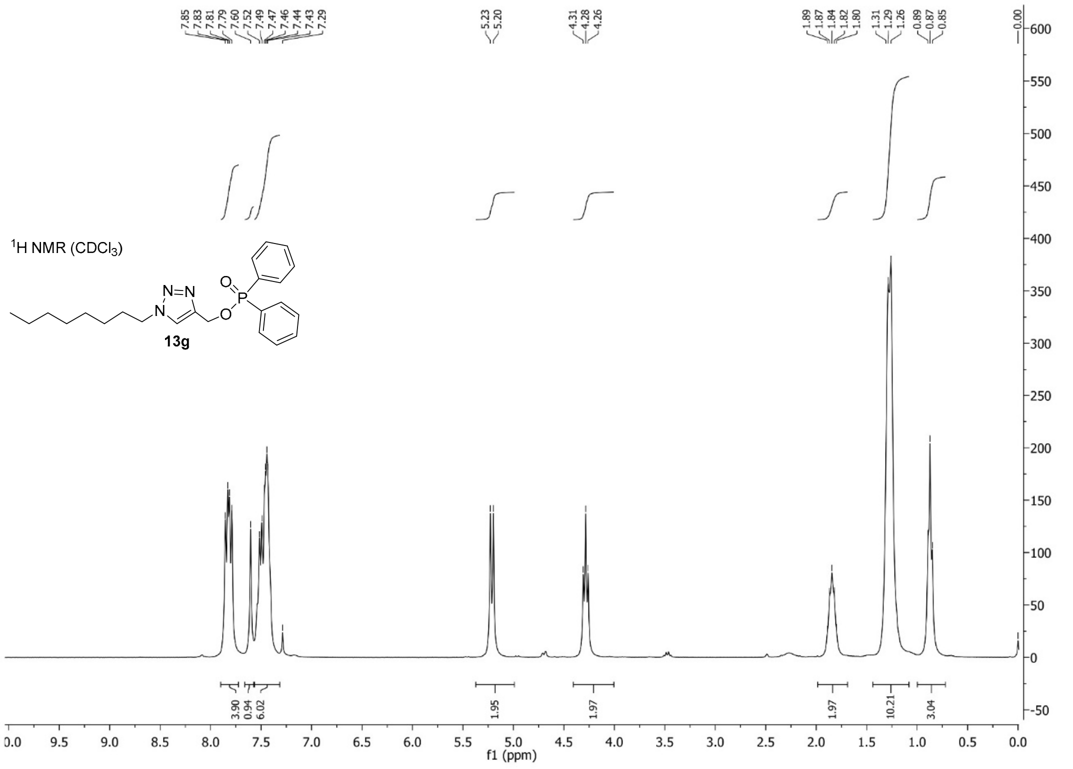

1H NMR (CDCl3)
13g
7.85 7.83 7.81 7.79 7.60 7.52 7.49 7.47 7.46 7.44 7.43 7.29
5.23 5.20
4.31 4.28 4.26
1.89 1.87 1.84 1.82 1.80 1.31 1.29 1.26 0.89 0.87 0.85
0.00
3.90 0.94 6.02 1.95 1.97 1.97 10.21 3.04
f1 (ppm)

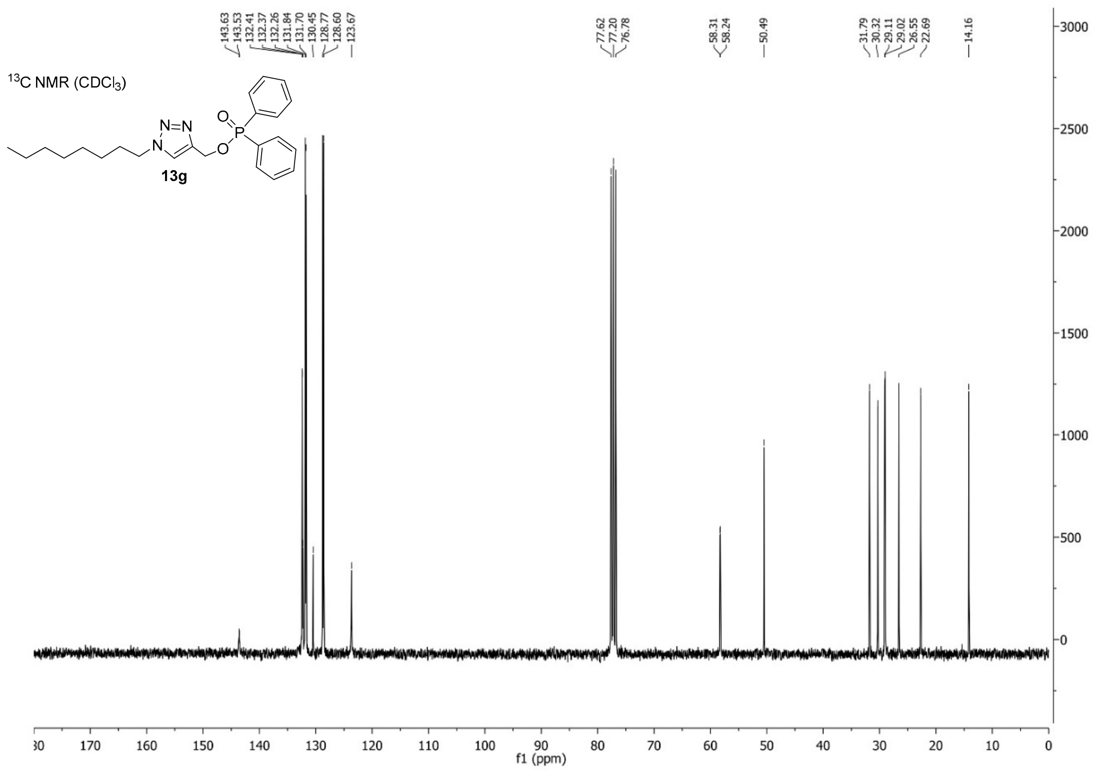

13C NMR (CDCl3)
13g
143.63
143.53
132.41
132.37
132.26
131.84
131.70
130.45
128.77
128.60
123.67
77.62
77.20
76.78
58.31
58.24
50.49
31.79
30.32
29.11
29.02
26.55
22.69
14.16
f1 (ppm)

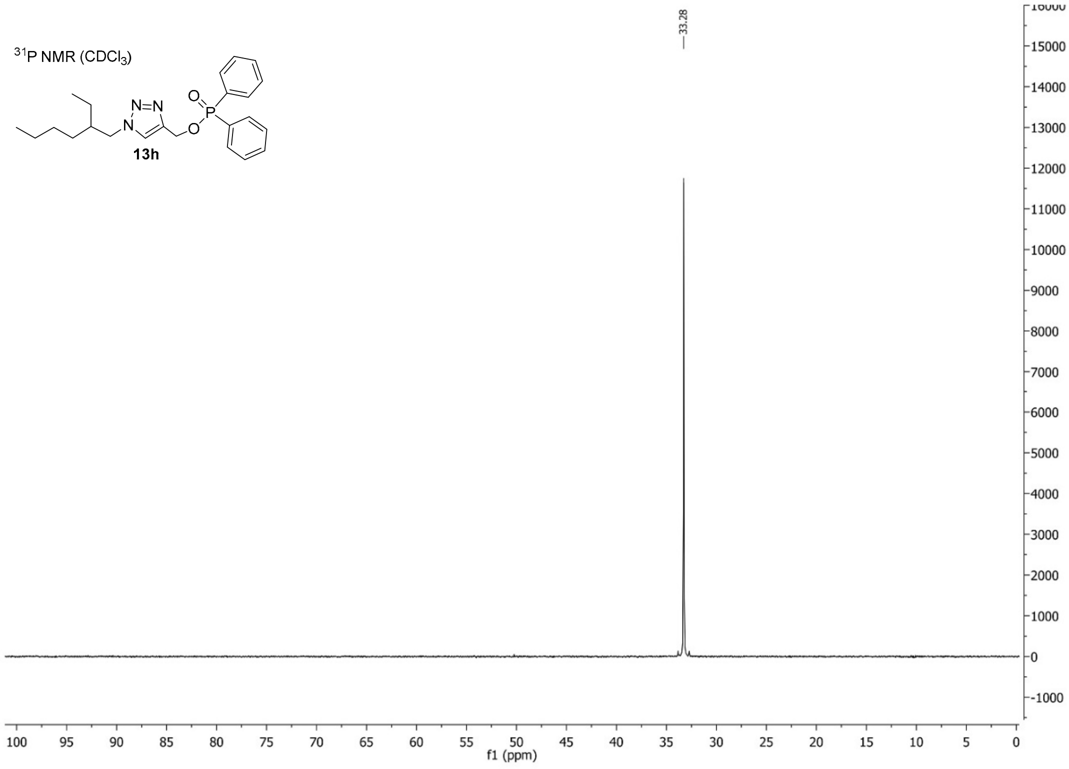

31P NMR (CDCl3)
13h
33.28
f1 (ppm)

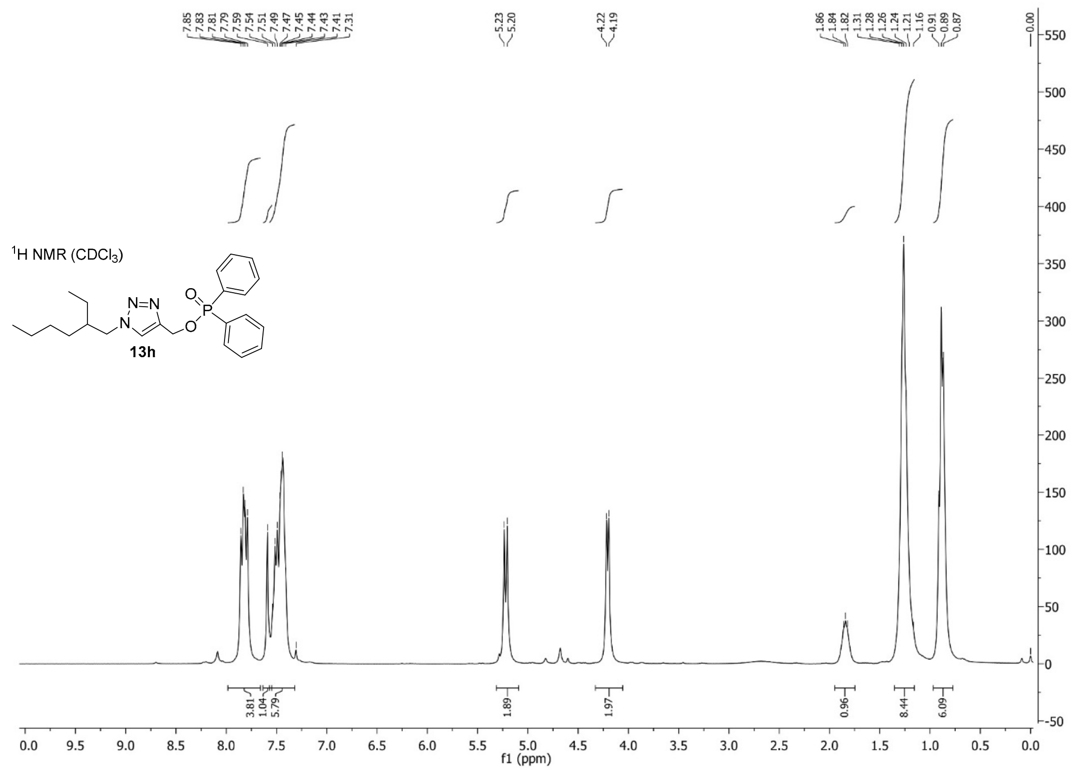
1H NMR (CDCl3)
13h
7.85
7.83
7.81
7.79
7.59
7.54
7.51
7.49
7.47
7.45
7.44
7.43
7.41
7.31
5.23
5.20
4.22
4.19
1.86
1.84
1.82
1.31
1.28
1.26
1.24
1.21
1.16
0.91
0.89
0.87
0.00
3.81
1.04
5.79
1.89
1.97
0.96
8.44
6.09
f1 (ppm)

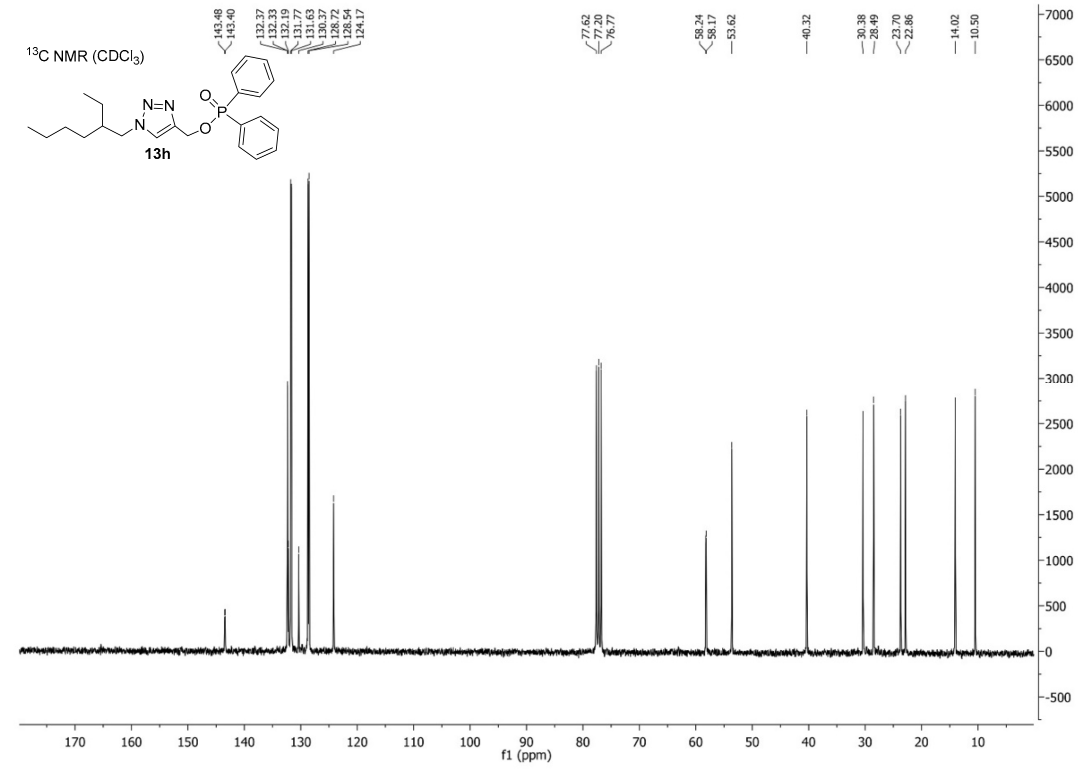
13C NMR (CDCl3)
13h
143.48
143.40
132.37
132.33
132.19
131.77
131.63
130.37
128.72
128.54
124.17
77.62
77.20
76.77
58.24
58.17
53.62
40.32
30.38
28.49
23.70
22.86
14.02
10.50
f1 (ppm)

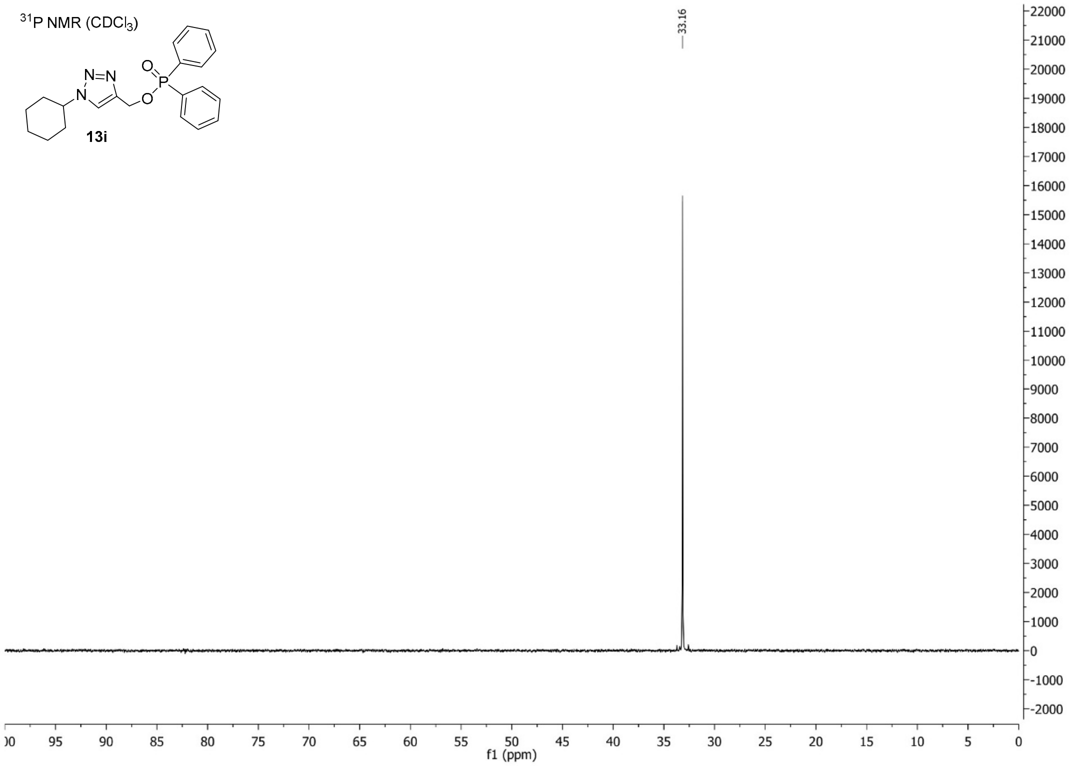

31P NMR (CDCl3)
13i
33.16
f1 (ppm)

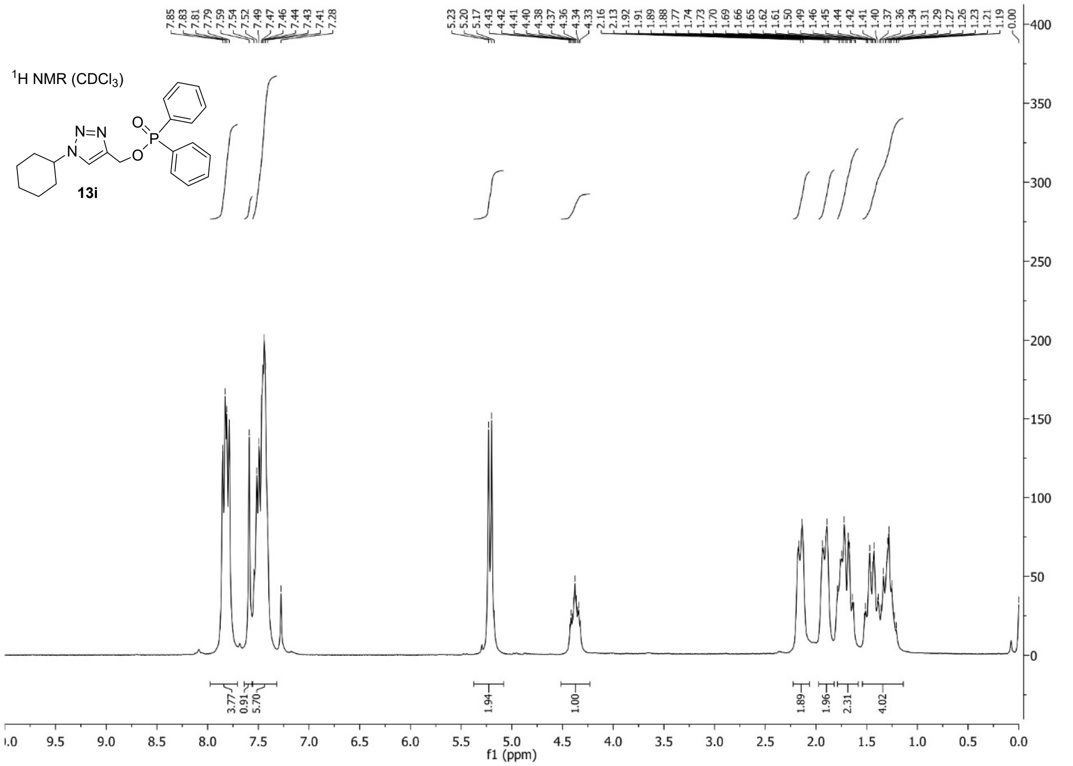

1H NMR (CDCl3)
13i
f1 (ppm)

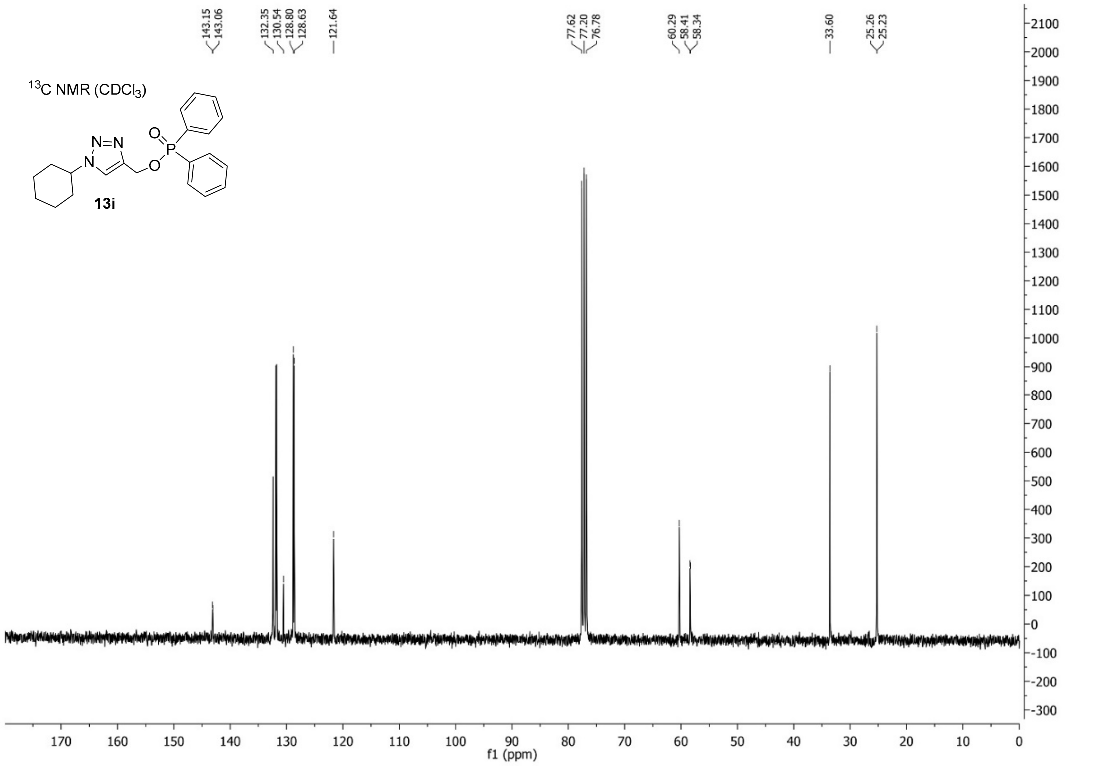

143.15
143.06
132.35
130.54
128.80
128.63
121.64
77.62
77.20
76.78
60.29
58.41
58.34
33.60
25.26
25.23
^{13}C NMR (CDCl$_3$)
13i
f1 (ppm)

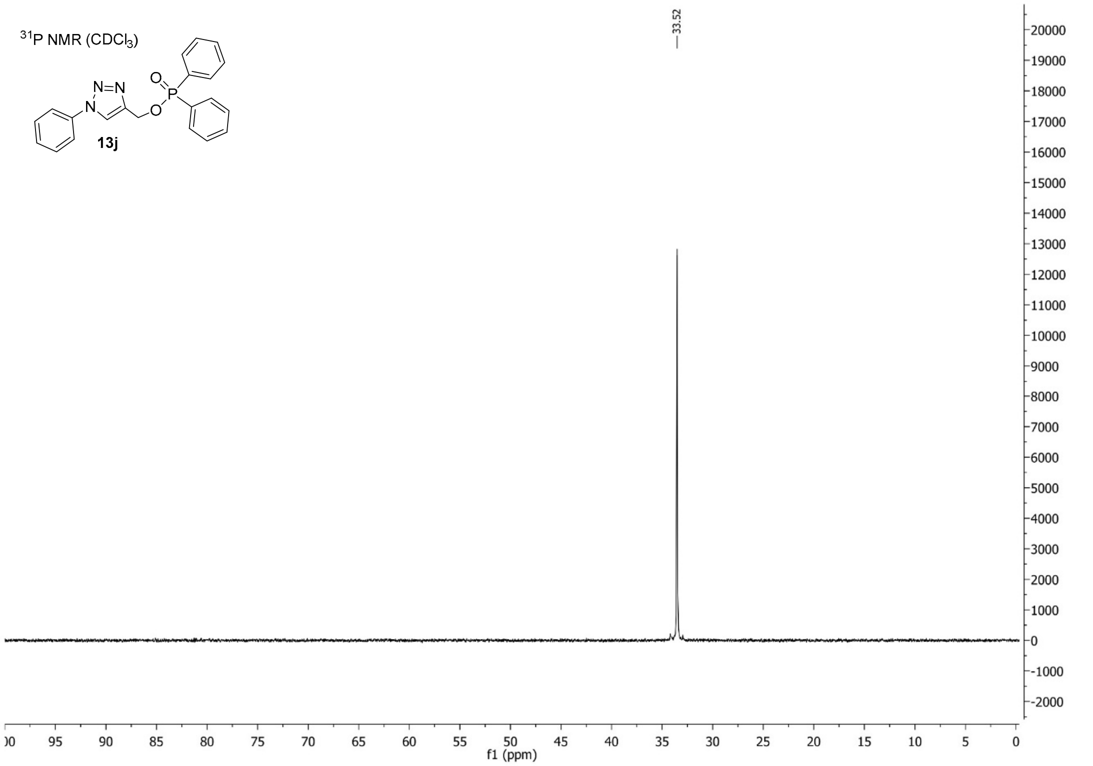

33.52
^{31}P NMR (CDCl$_3$)
13j
f1 (ppm)

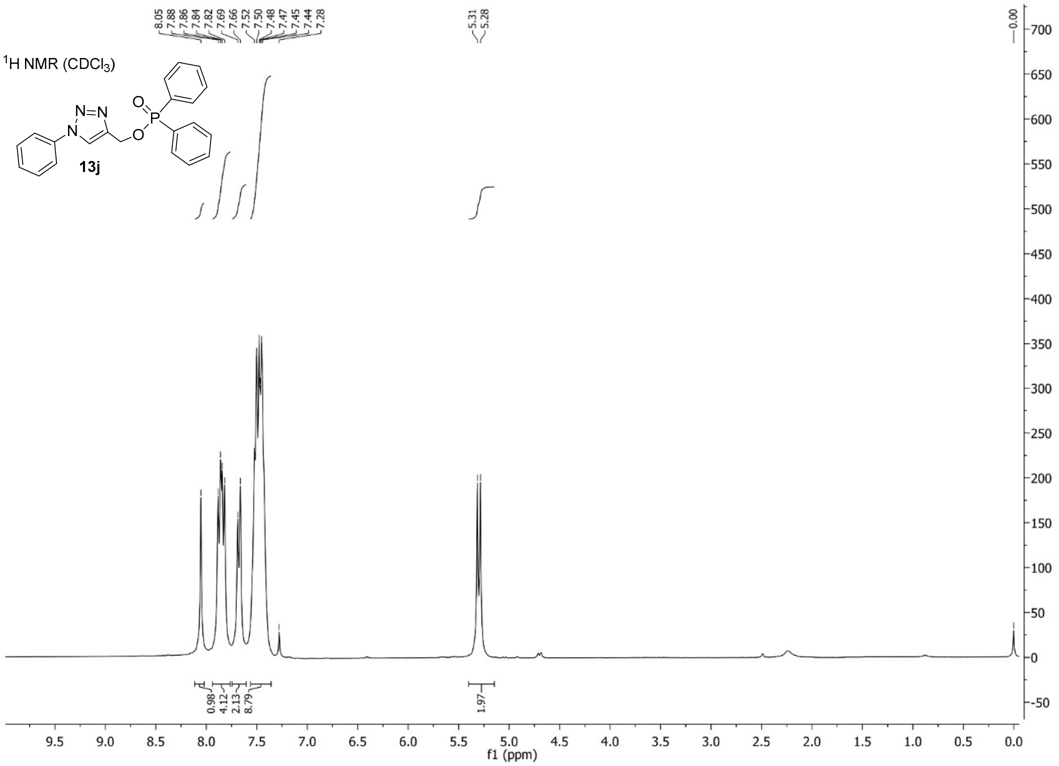

8.05
7.88
7.86
7.84
7.82
7.69
7.66
7.52
7.50
7.48
7.47
7.45
7.44
7.28
5.31
5.28
0.00
^{1}H NMR ($CDCl_3$)
13j
0.98
4.12
2.13
8.79
1.97
f1 (ppm)

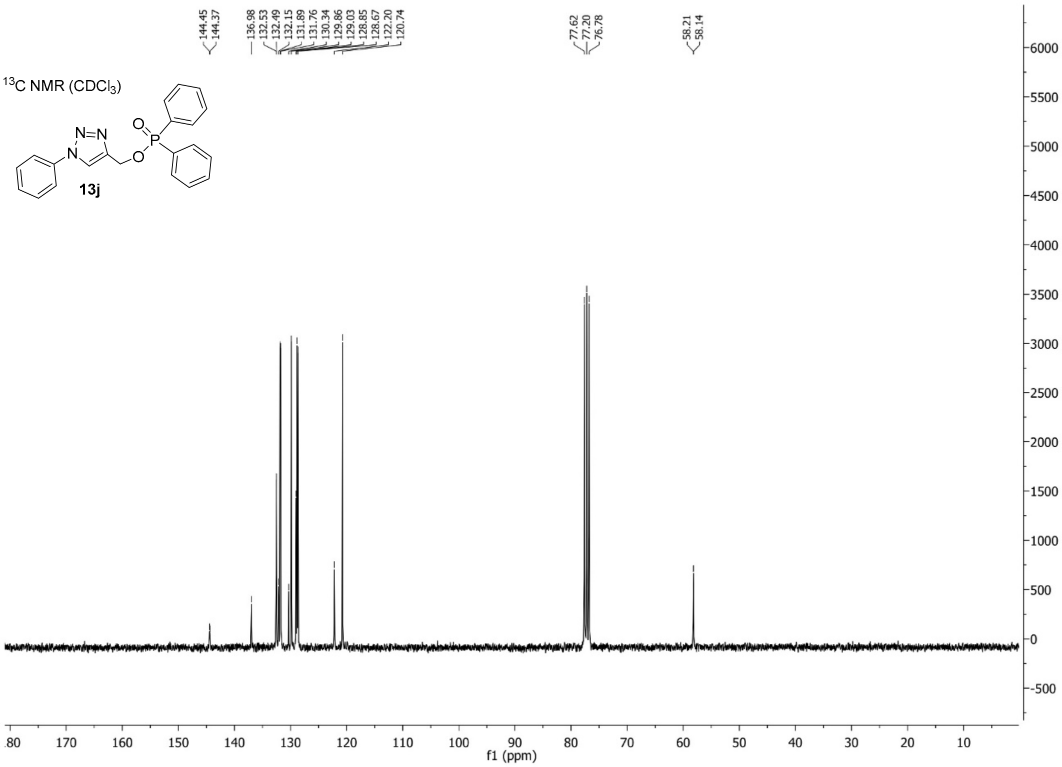

144.45
144.37
136.98
132.53
132.49
132.15
131.89
131.76
130.34
129.86
129.03
128.85
128.67
122.20
120.74
77.62
77.20
76.78
58.21
58.14
^{13}C NMR ($CDCl_3$)
13j
f1 (ppm)

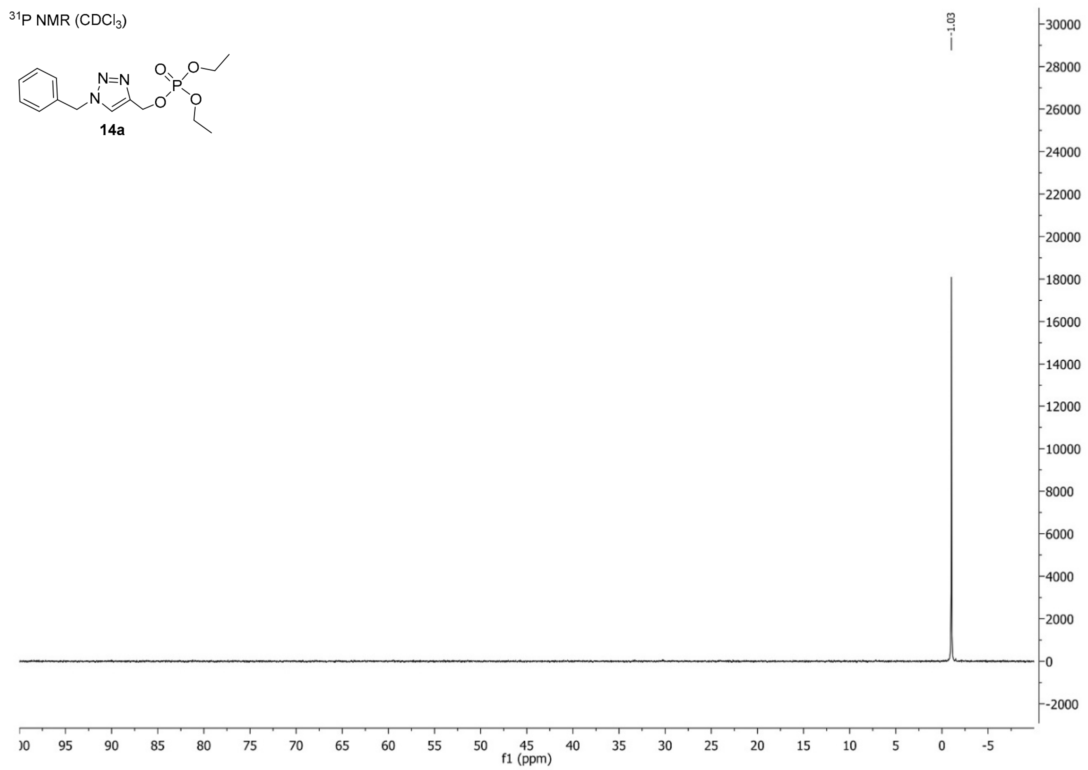
^{31}P NMR (CDCl$_3$)
14a
-1.03
f1 (ppm)

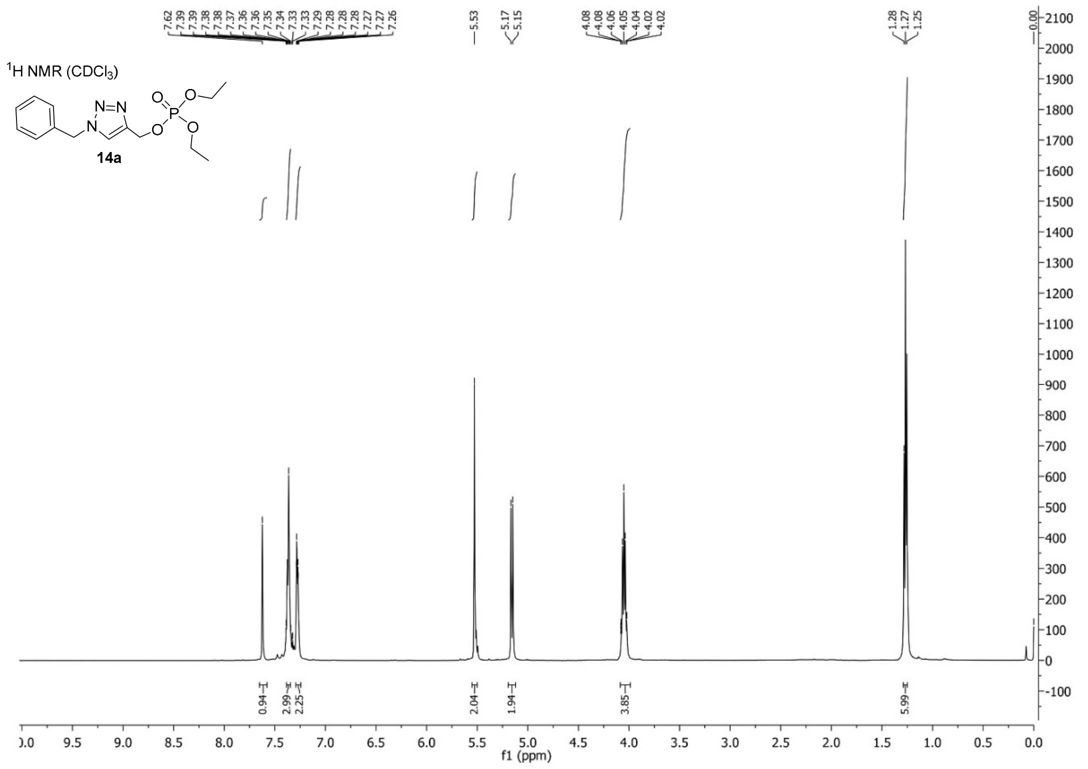
^{1}H NMR (CDCl$_3$)
14a
7.62 7.39 7.39 7.38 7.38 7.37 7.36 7.36 7.35 7.34 7.33 7.33 7.29 7.28 7.28 7.28 7.27 7.27 7.26
5.53
5.17 5.15
4.08 4.08 4.06 4.05 4.04 4.02 4.02
1.28 1.27 1.25
0.00
0.94 2.99 2.25 2.04 1.94 3.85 5.99
f1 (ppm)

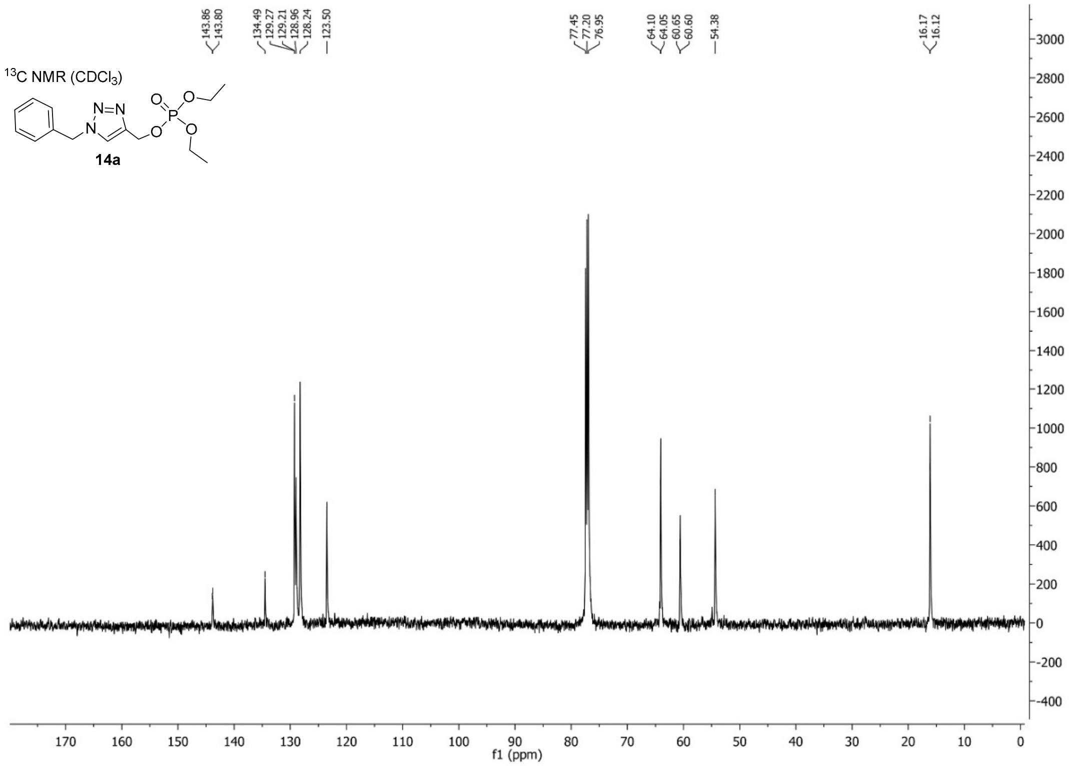
143.86
143.80
134.49
129.27
129.21
128.96
128.24
123.50
77.45
77.20
76.95
64.10
64.05
60.65
60.60
54.38
16.17
16.12
^{13}C NMR (CDCl$_3$)
14a
f1 (ppm)

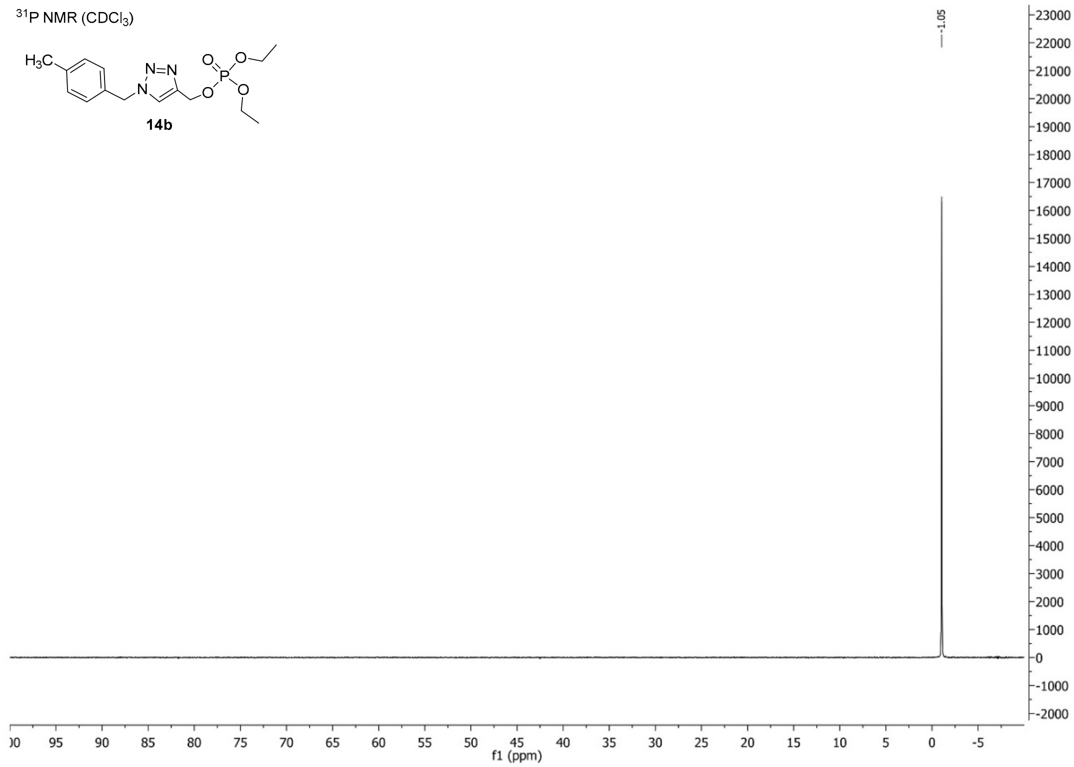
^{31}P NMR (CDCl$_3$)
14b
-1.05
f1 (ppm)

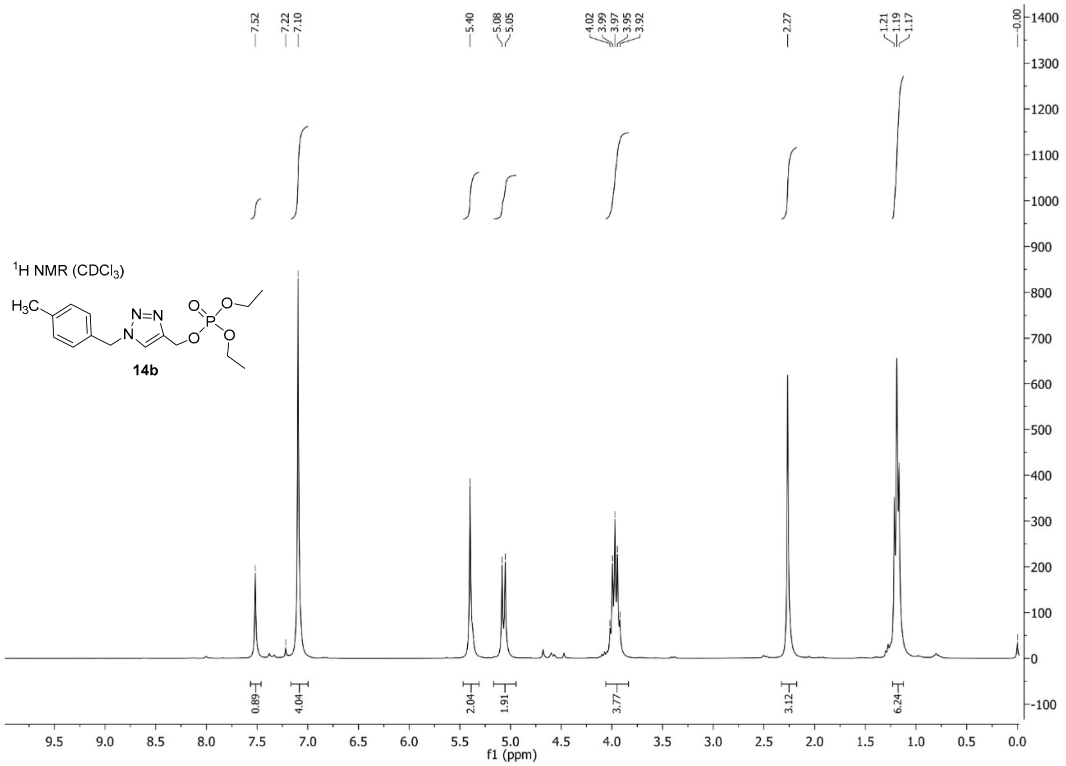
1H NMR (CDCl3)
14b
f1 (ppm)

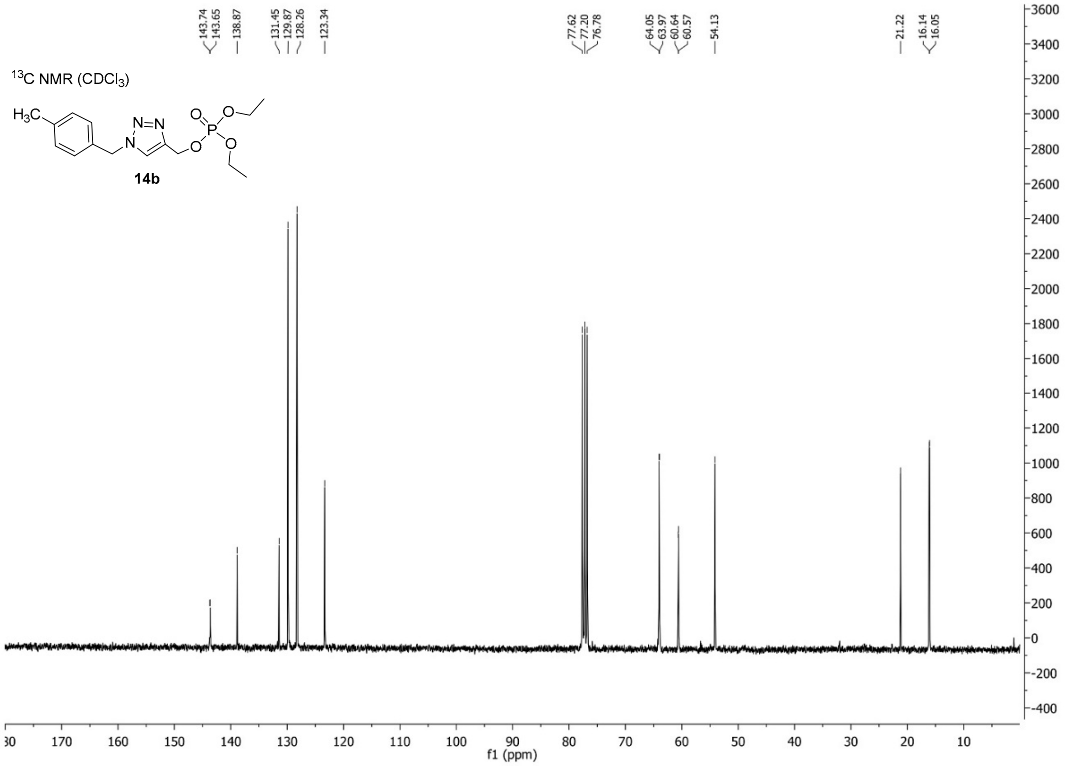
13C NMR (CDCl3)
14b
f1 (ppm)

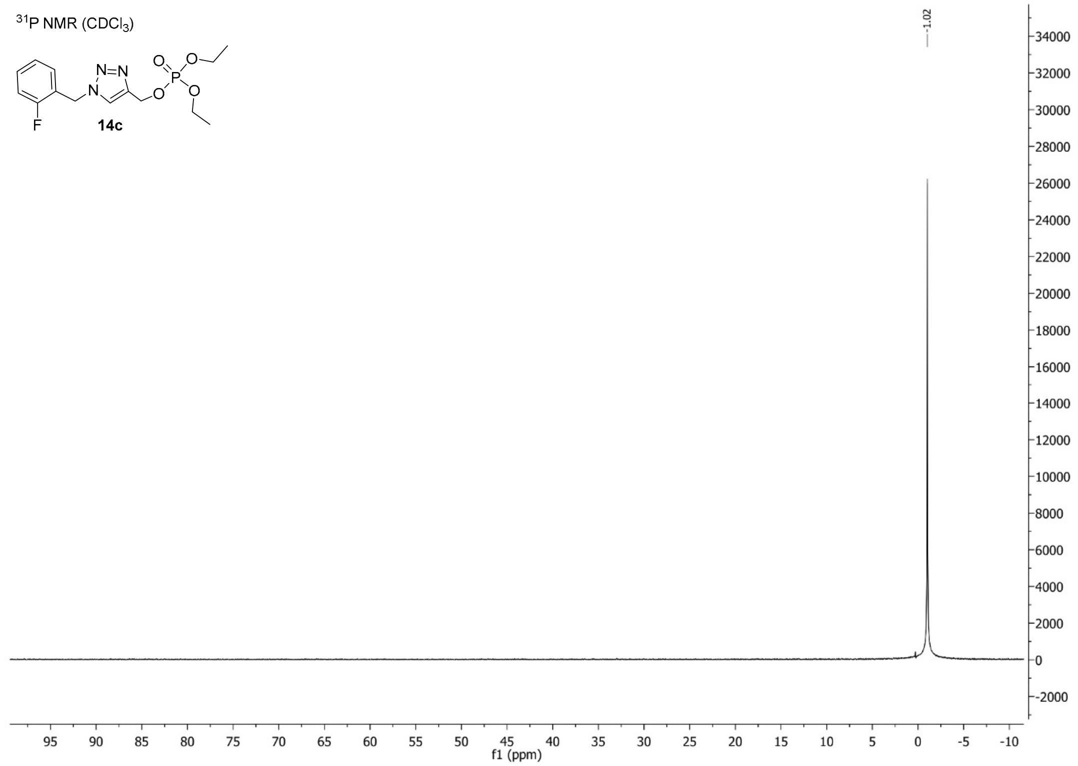

^{31}P NMR ($CDCl_3$)
14c
F
-1.02
f1 (ppm)

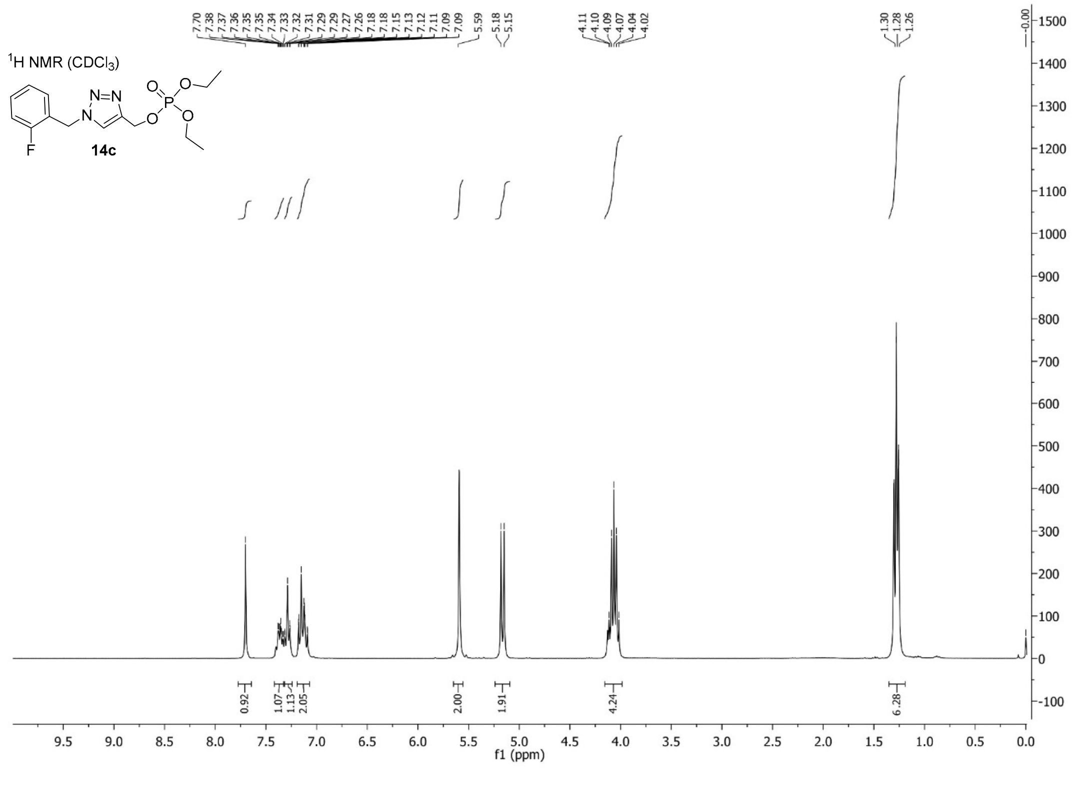

^{1}H NMR ($CDCl_3$)
14c
F
f1 (ppm)
0.92
1.07
1.13
2.05
2.00
1.91
4.24
6.28

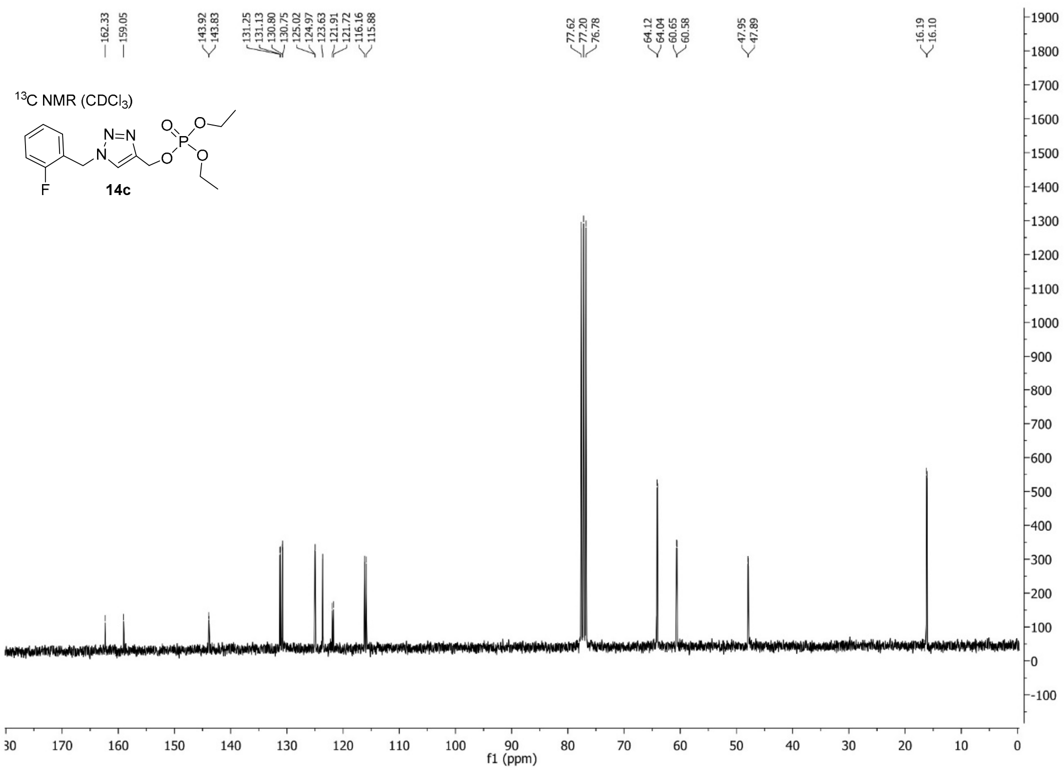
162.33
159.05
143.92
143.83
131.25
131.13
130.80
130.75
125.02
124.97
123.63
121.91
121.72
116.16
115.88
77.62
77.20
76.78
64.12
64.04
60.65
60.58
47.95
47.89
16.19
16.10
13C NMR (CDCl3)
F
14c
f1 (ppm)

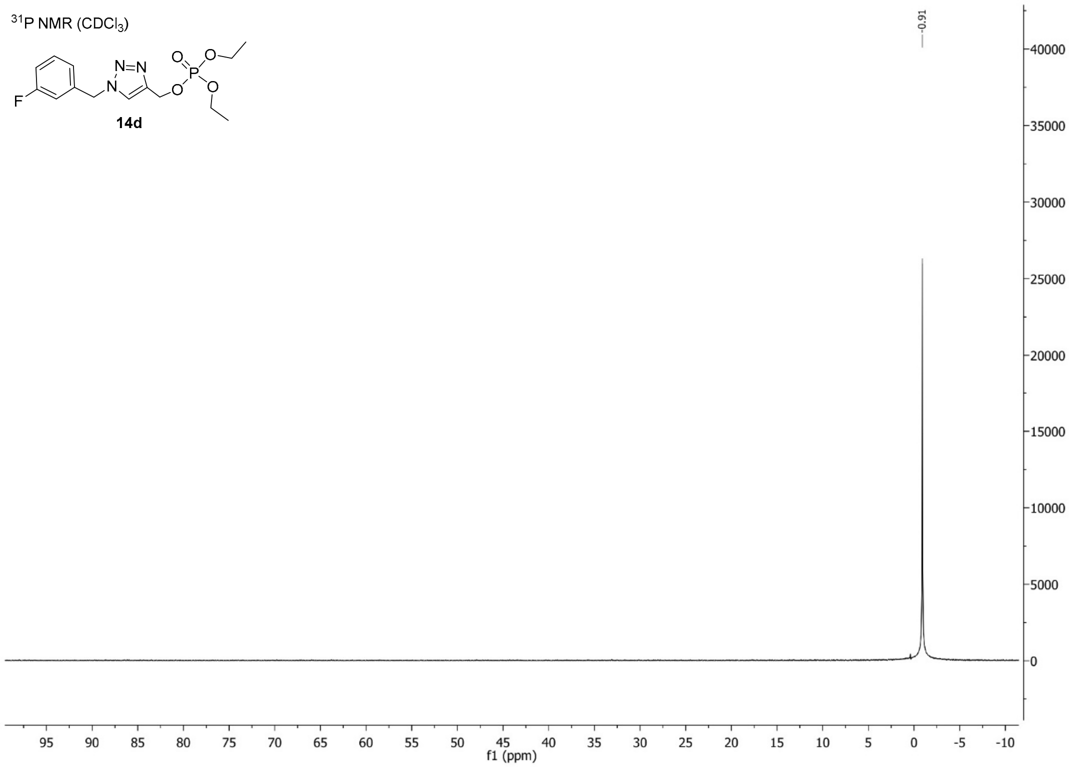
-0.91
31P NMR (CDCl3)
F
14d
f1 (ppm)

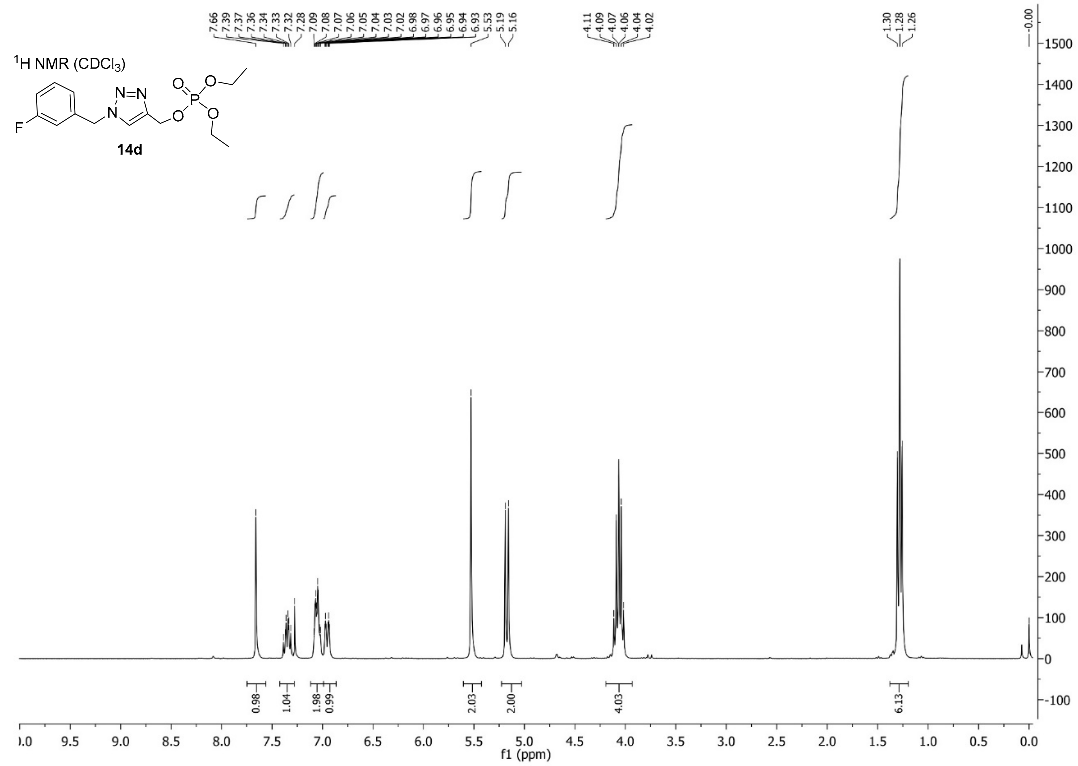
7.66 7.39 7.37 7.36 7.34 7.33 7.32 7.28 7.09 7.08 7.07 7.06 7.05 7.04 7.03 7.02 6.98 6.97 6.96 6.95 6.94 6.93 5.53 5.19 5.16
4.11 4.09 4.07 4.06 4.04 4.02
1.30 1.28 1.26
0.00
1H NMR (CDCl3)
F
N
N
N
O
P
O
O
O
14d
0.98 1.04 1.98 0.99 2.03 2.00 4.03 6.13
f1 (ppm)

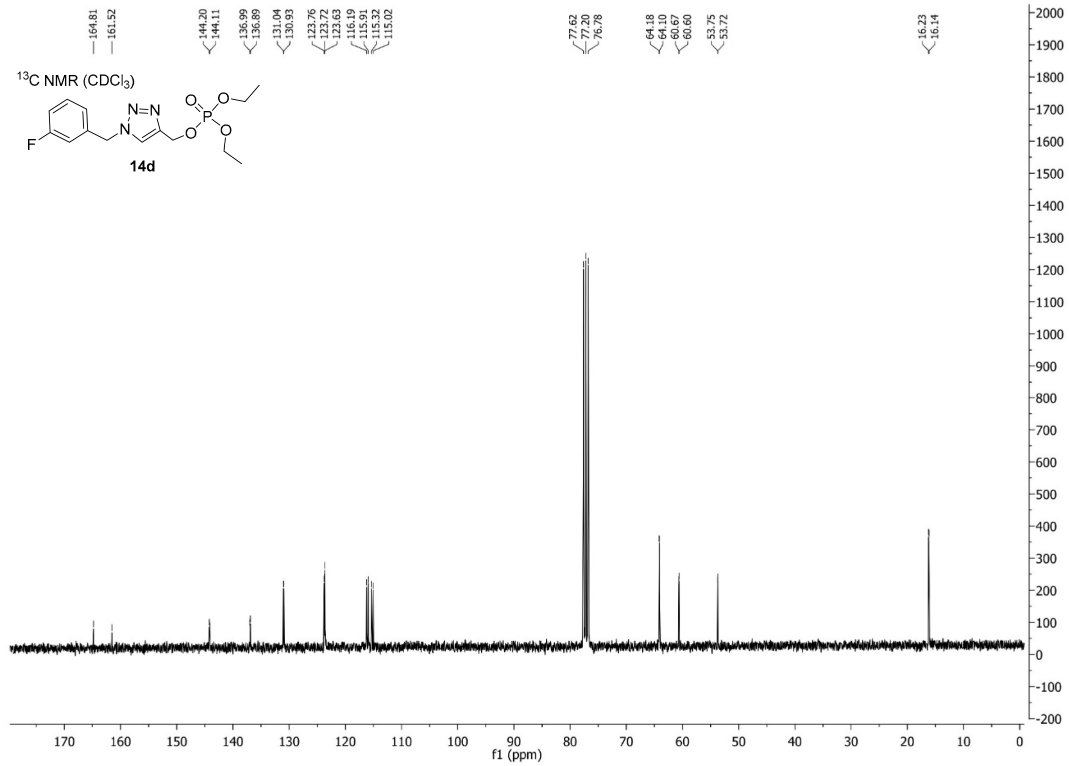
164.81 161.52 144.20 144.11 136.99 136.89 131.04 130.93 123.76 123.72 123.63 116.19 115.91 115.32 115.02
77.62 77.20 76.78 64.18 64.10 60.67 60.60 53.75 53.72
16.23 16.14
13C NMR (CDCl3)
F
N
N
N
O
P
O
O
O
14d
f1 (ppm)

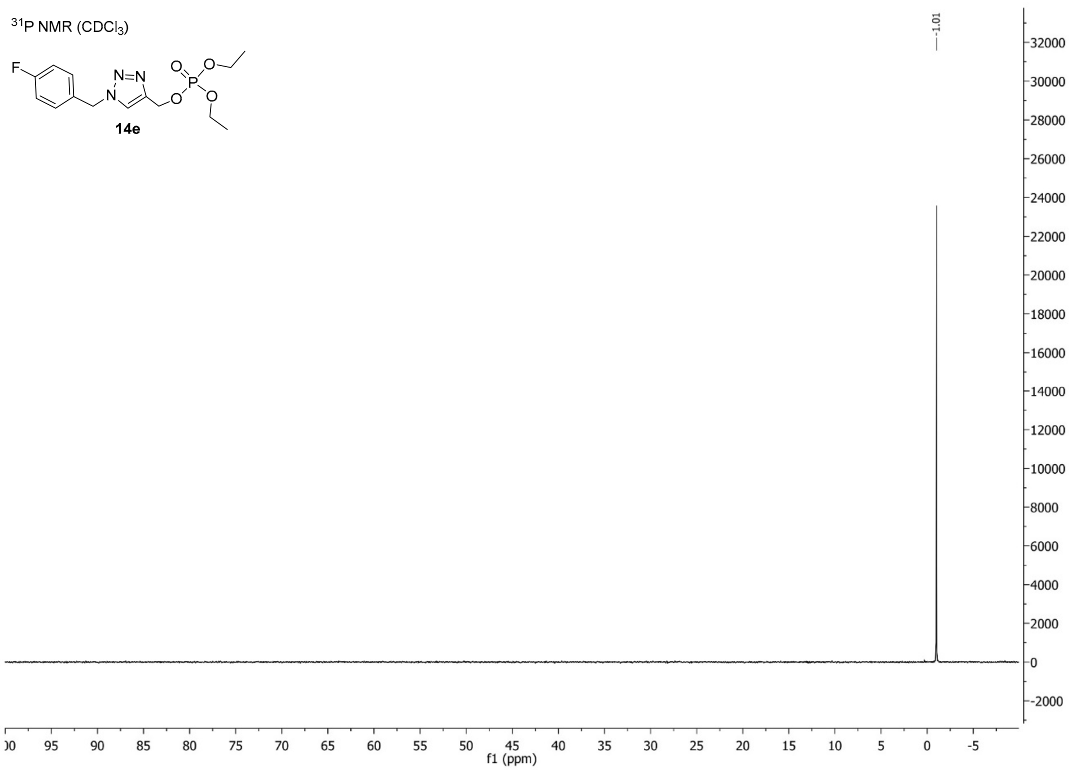

31P NMR (CDCl3)
14e
-1.01
f1 (ppm)

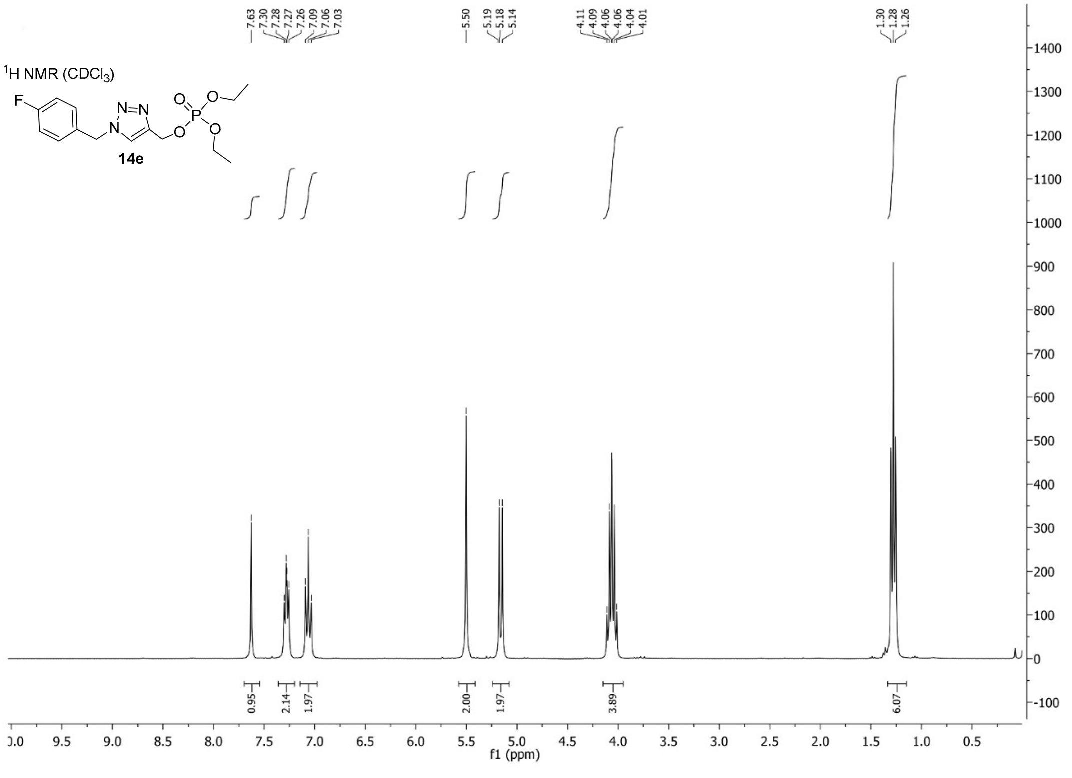

1H NMR (CDCl3)
14e
f1 (ppm)

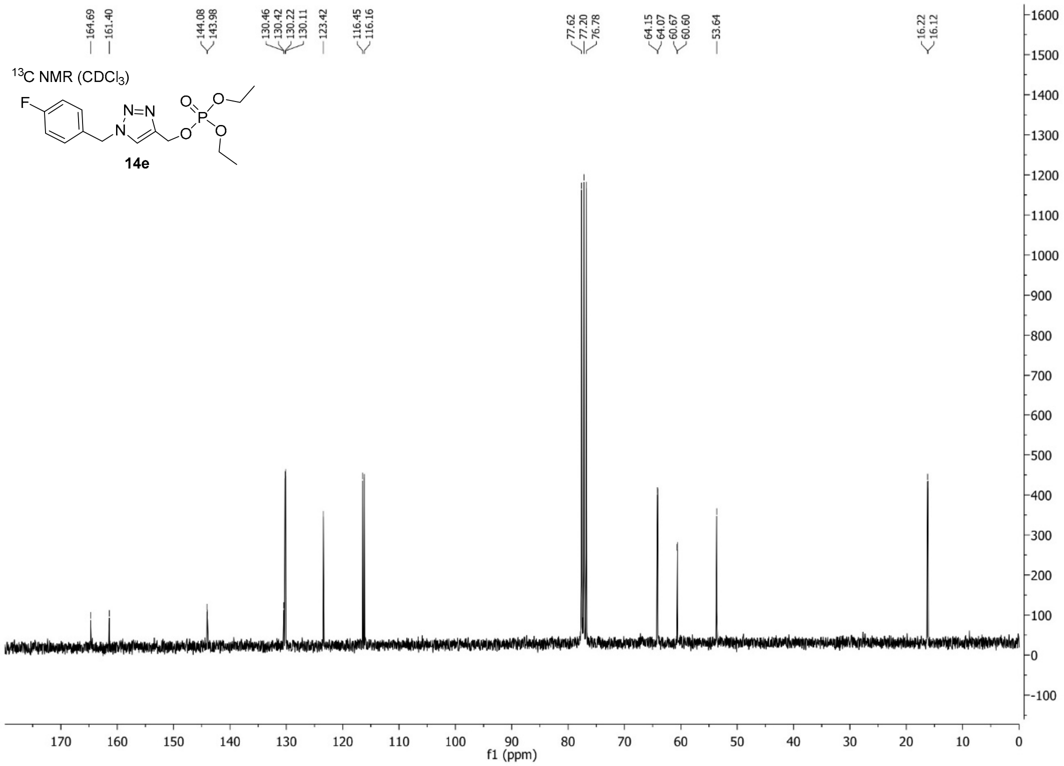

13C NMR (CDCl3)
14e

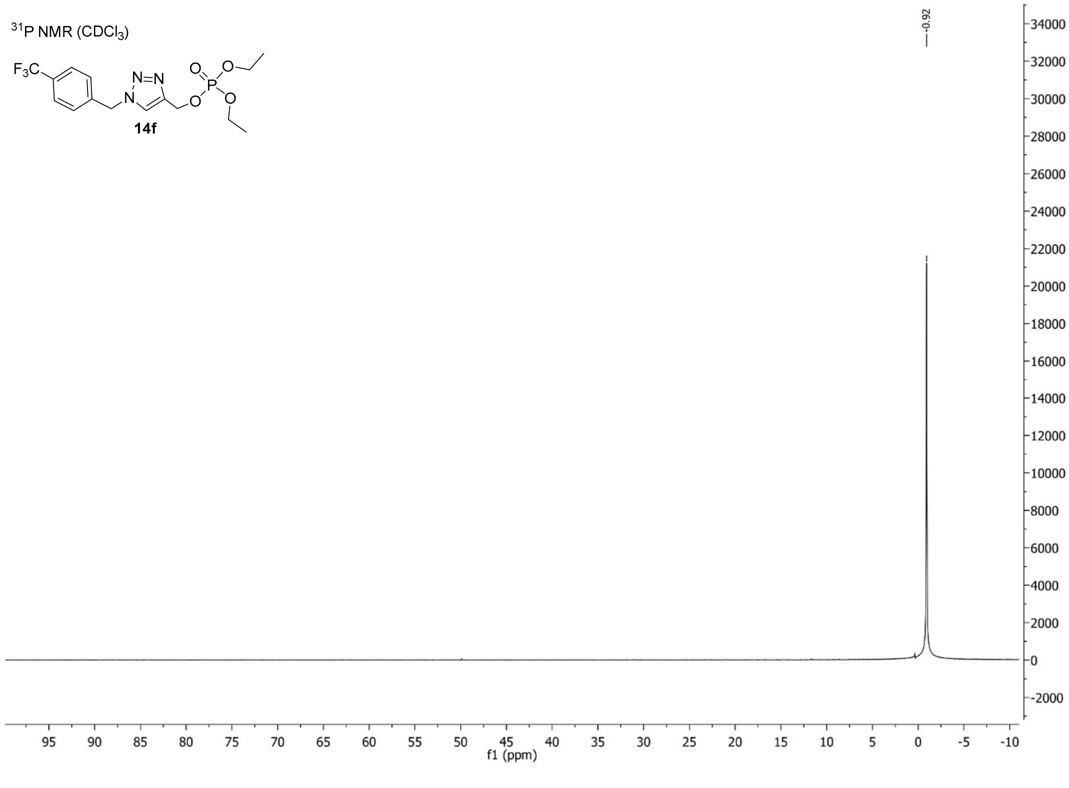

31P NMR (CDCl3)
14f

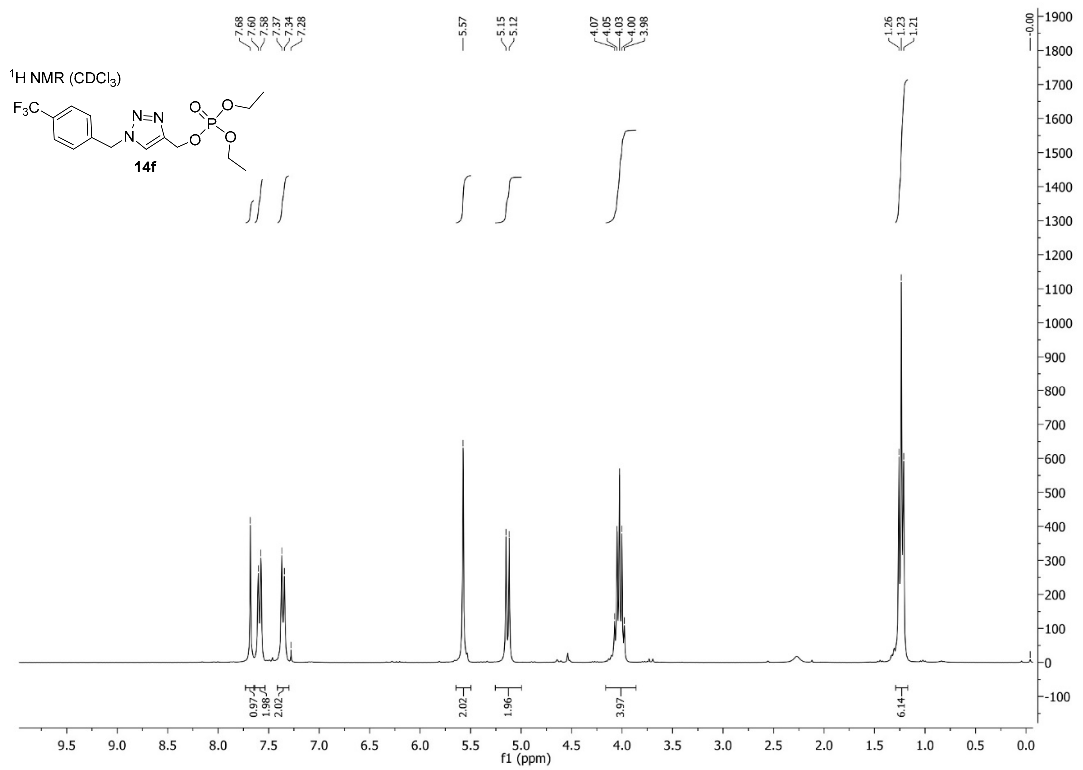

^{1}H NMR (CDCl$_3$)
14f

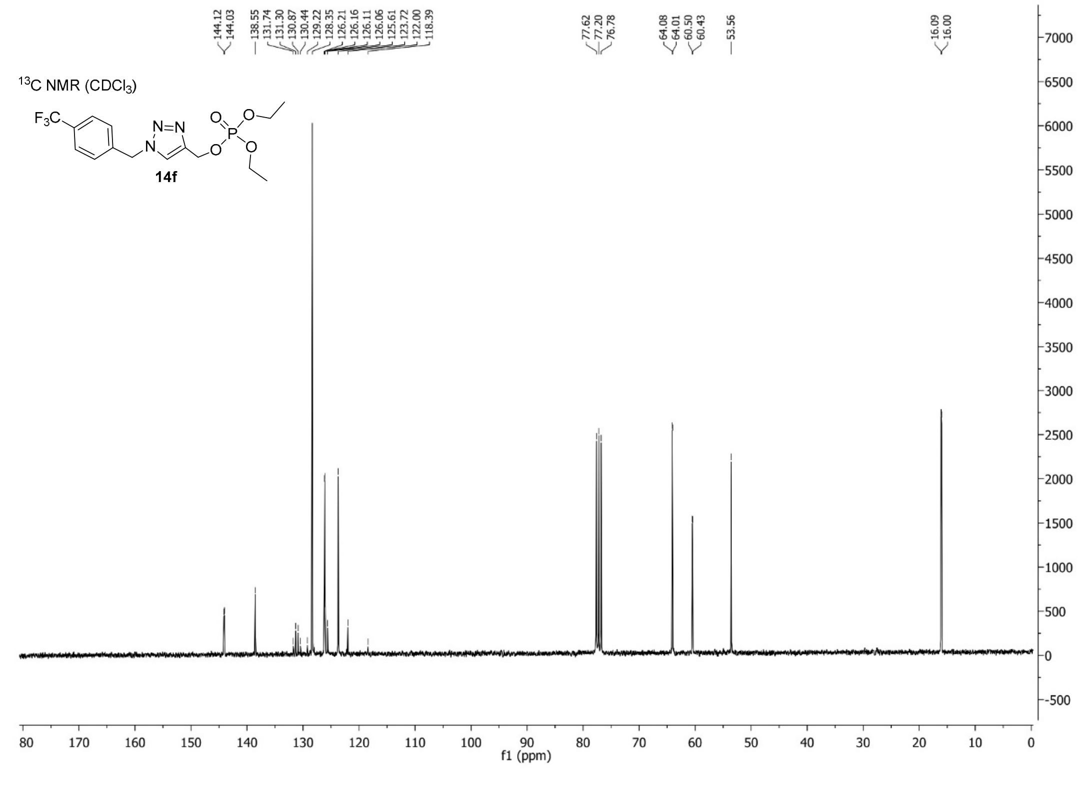

^{13}C NMR (CDCl$_3$)
14f

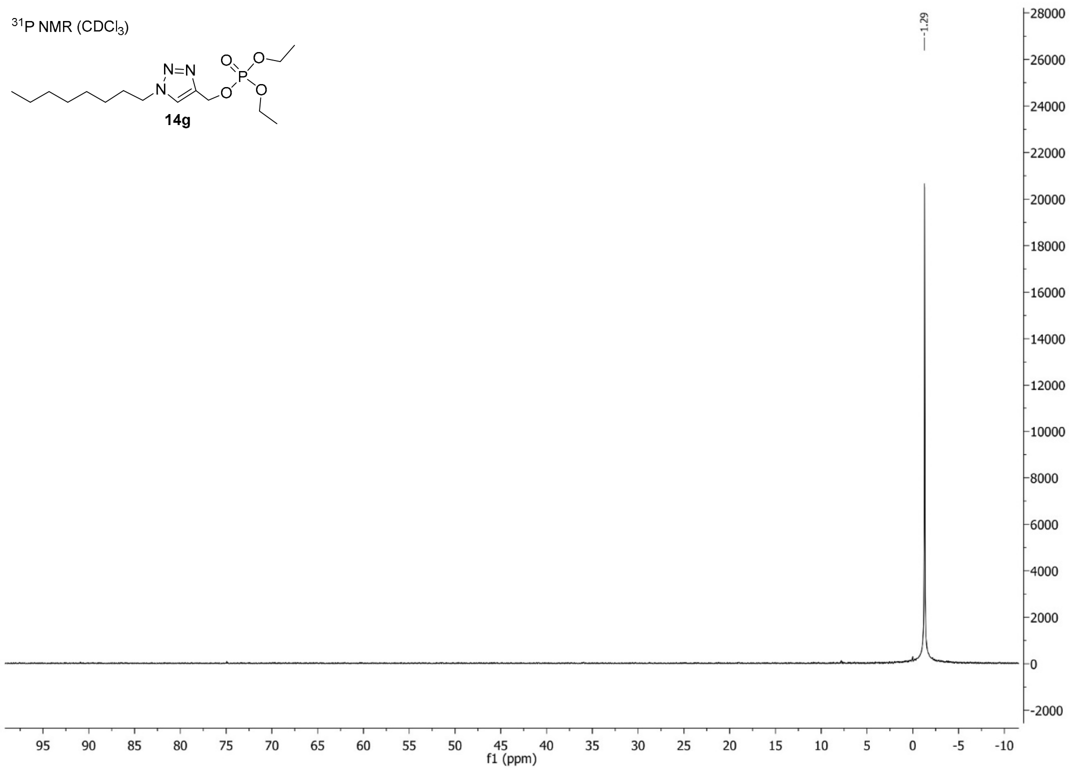

31P NMR (CDCl3)
14g
-1.29
f1 (ppm)

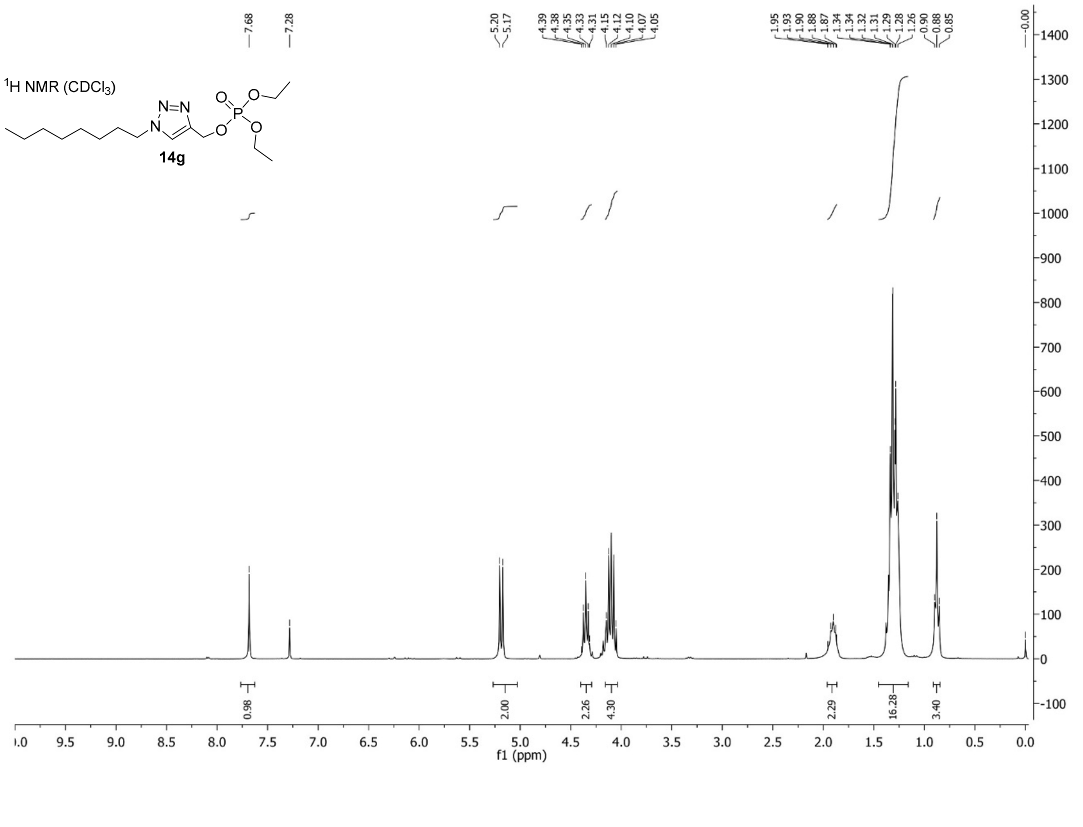

1H NMR (CDCl3)
14g
7.68
7.28
5.20
5.17
4.39
4.38
4.35
4.33
4.31
4.15
4.12
4.10
4.07
4.05
1.95
1.93
1.90
1.88
1.87
1.34
1.34
1.32
1.31
1.29
1.28
1.26
0.90
0.88
0.85
0.00
0.98
2.00
2.26
4.30
2.29
16.28
3.40
f1 (ppm)

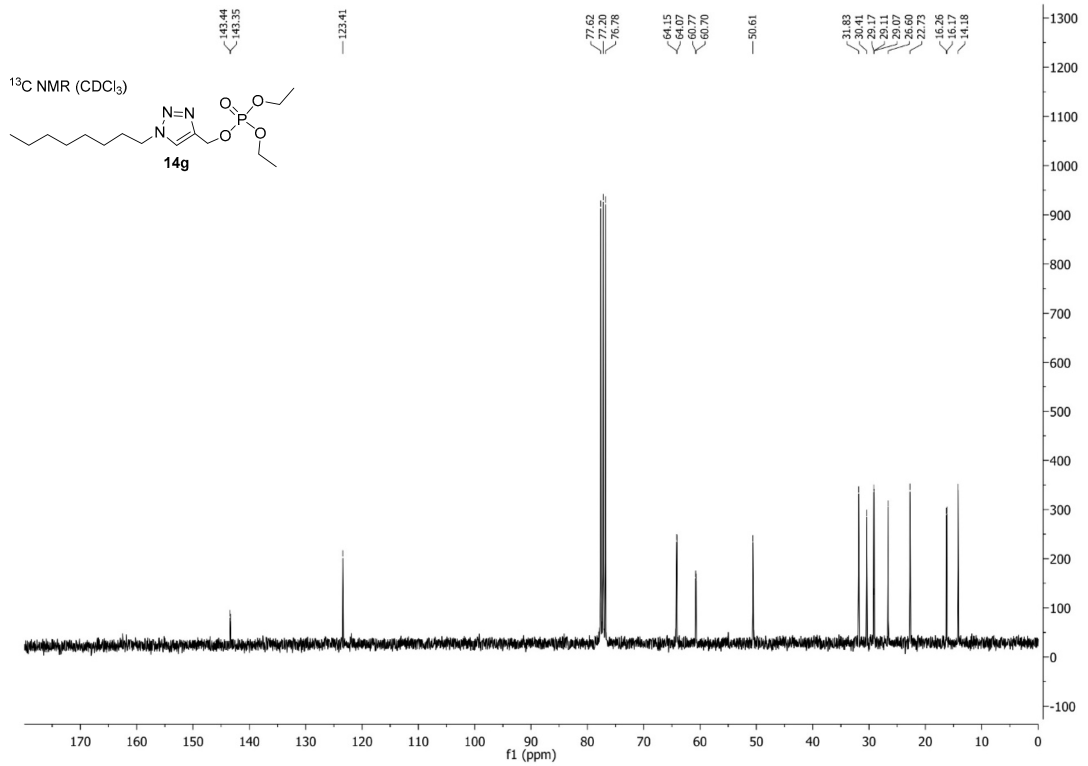

13C NMR (CDCl3)
N=N
N
O
P
O
O
O
14g
143.44
143.35
123.41
77.62
77.20
76.78
64.15
64.07
60.77
60.70
50.61
31.83
30.41
29.17
29.11
29.07
26.60
22.73
16.26
16.17
14.18
f1 (ppm)

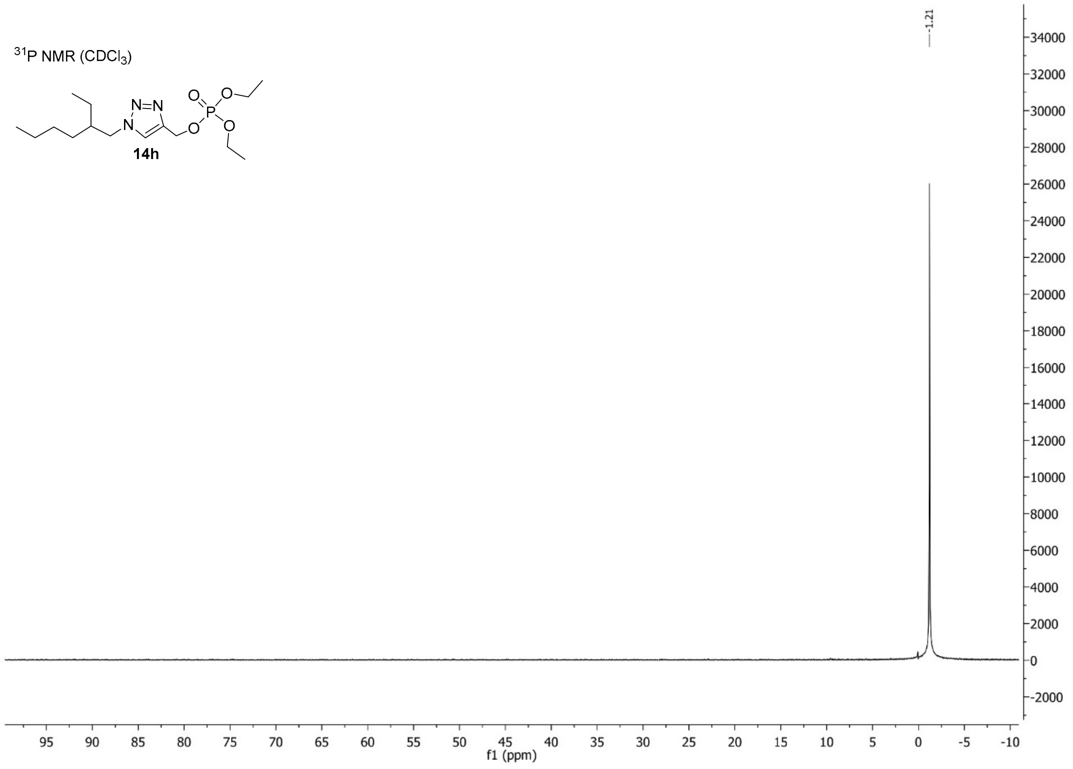

31P NMR (CDCl3)
N=N
N
O
P
O
O
O
14h
-1.21
f1 (ppm)

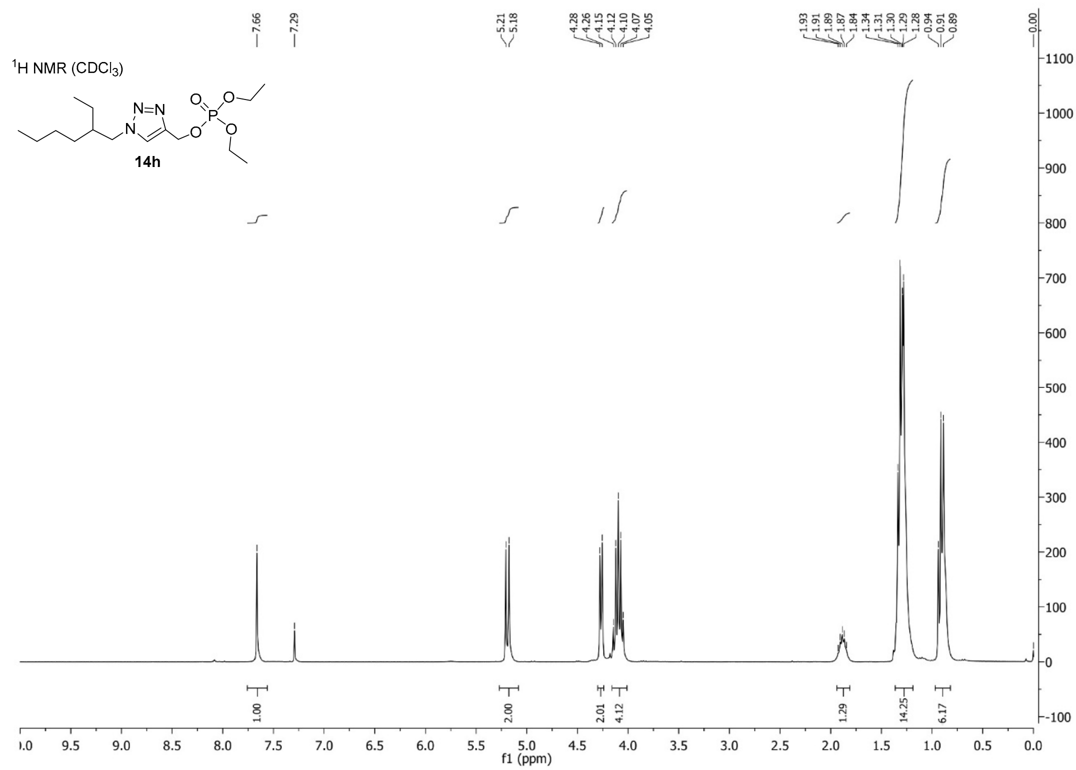
1H NMR (CDCl3)
14h
f1 (ppm)

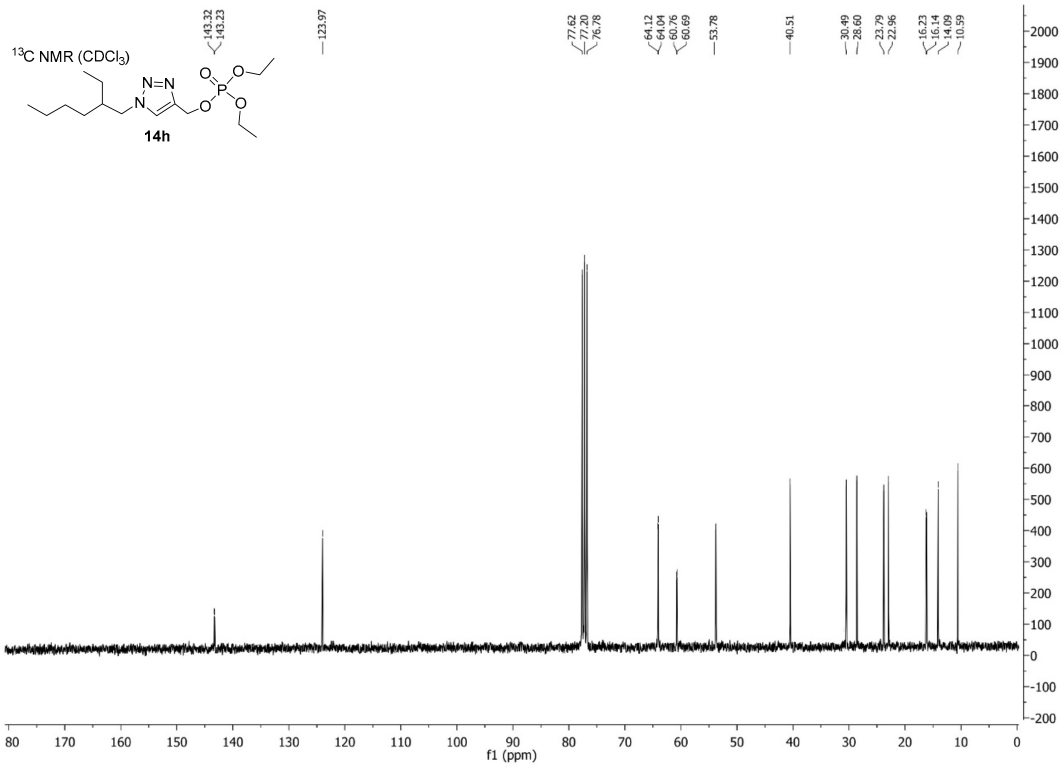
13C NMR (CDCl3)
14h
f1 (ppm)

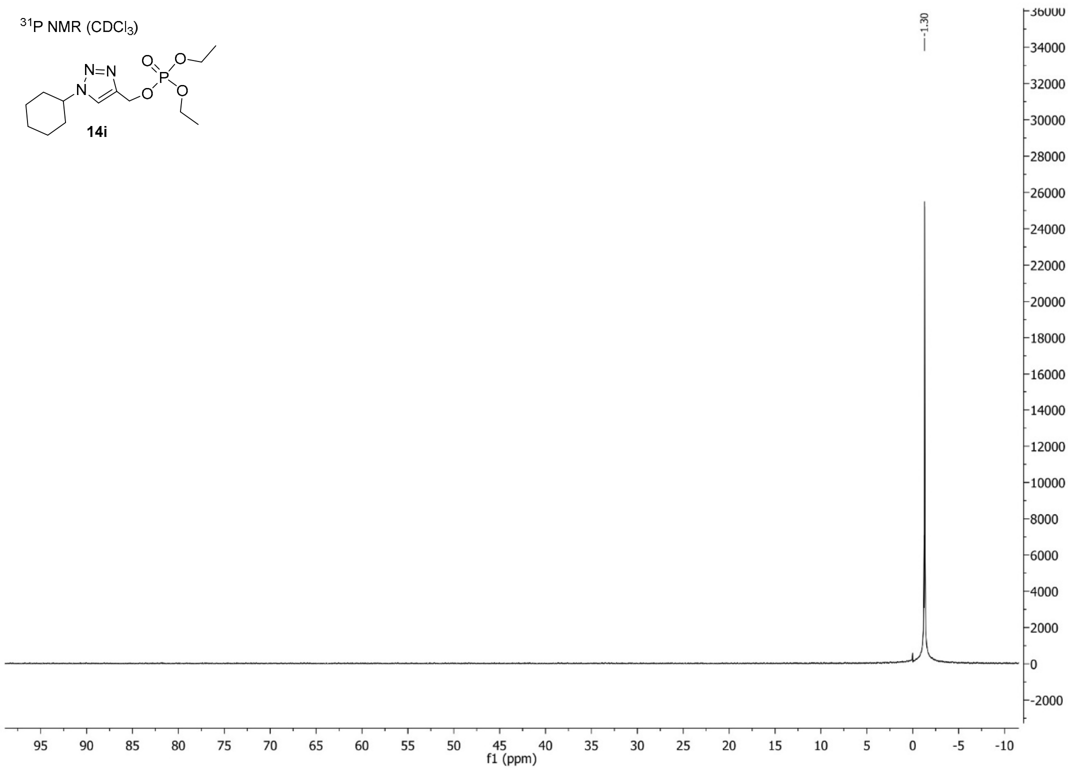

^{31}P NMR ($CDCl_3$)
14i
-1.30
f1 (ppm)

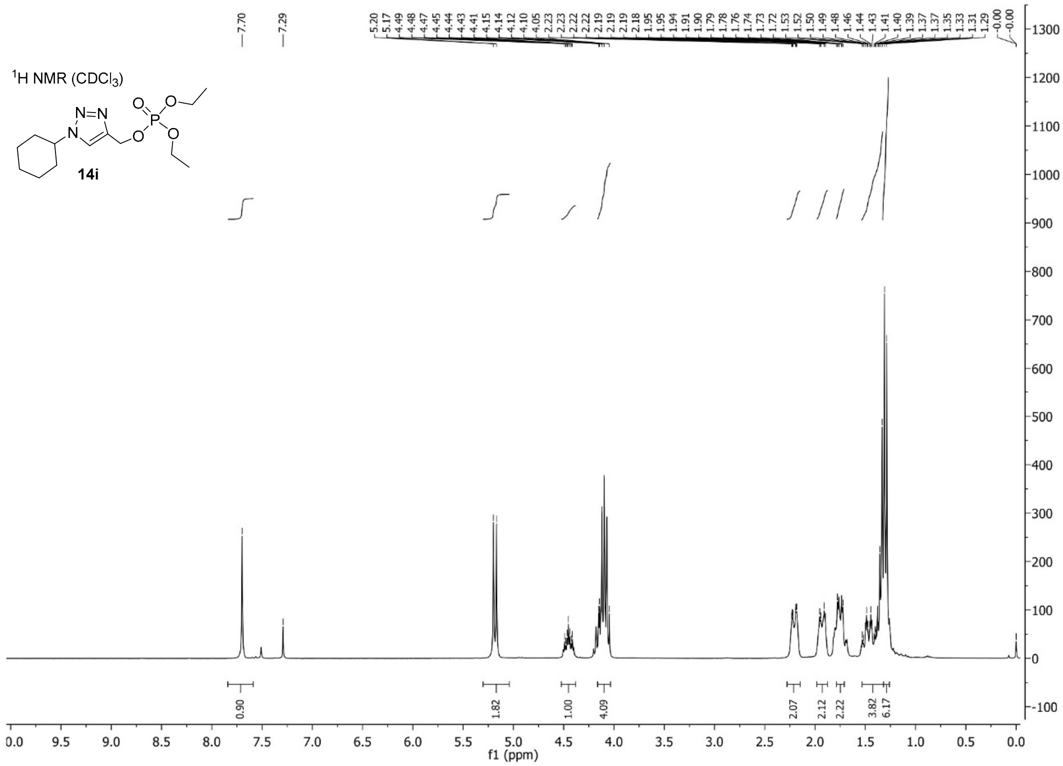

^{1}H NMR ($CDCl_3$)
14i
7.70
7.29
5.20 5.17 4.49 4.48 4.47 4.45 4.44 4.43 4.41 4.15 4.14 4.12 4.10 4.05 2.23 2.23 2.22 2.22 2.19 2.19 2.19 2.18 1.95 1.95 1.94 1.91 1.90 1.79 1.78 1.76 1.74 1.73 1.72 1.53 1.52 1.50 1.49 1.48 1.46 1.44 1.43 1.41 1.40 1.39 1.37 1.37 1.35 1.33 1.31 1.29 -0.00 -0.00
0.90
1.82
1.00
4.09
2.07
2.12
2.22
3.82
6.17
f1 (ppm)

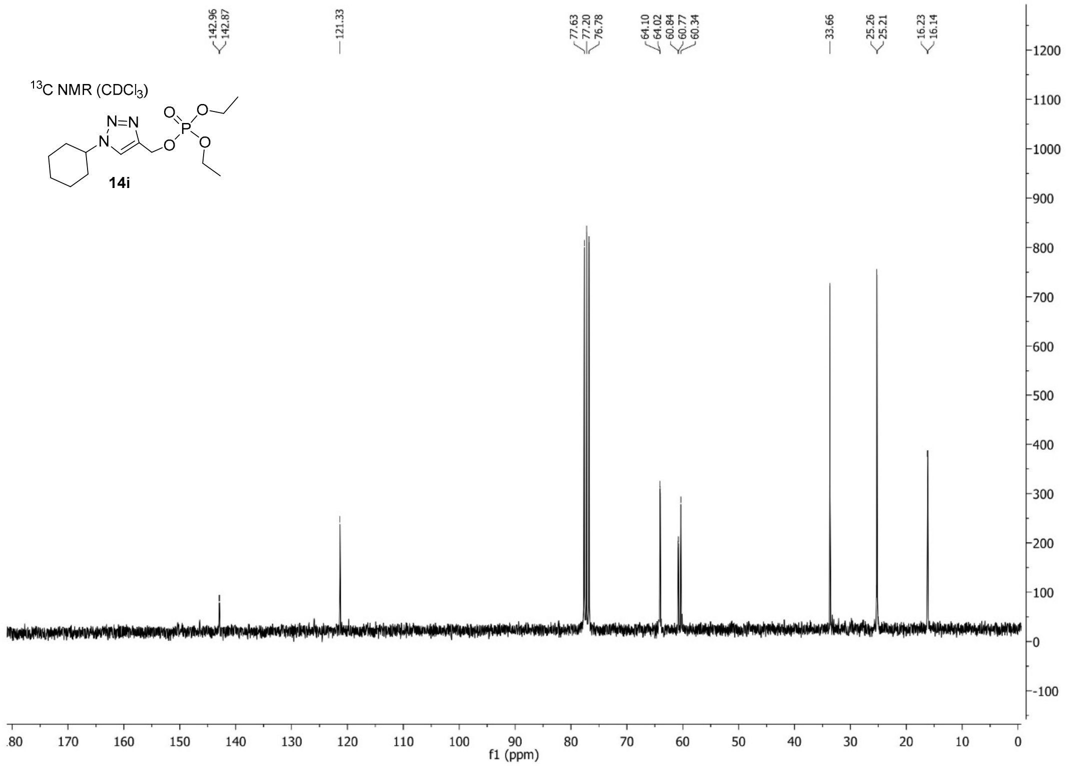

142.96
142.87
121.33
77.63
77.20
76.78
64.10
64.02
60.84
60.77
60.34
33.66
25.26
25.21
16.23
16.14
^{13}C NMR (CDCl$_3$)
14i
f1 (ppm)

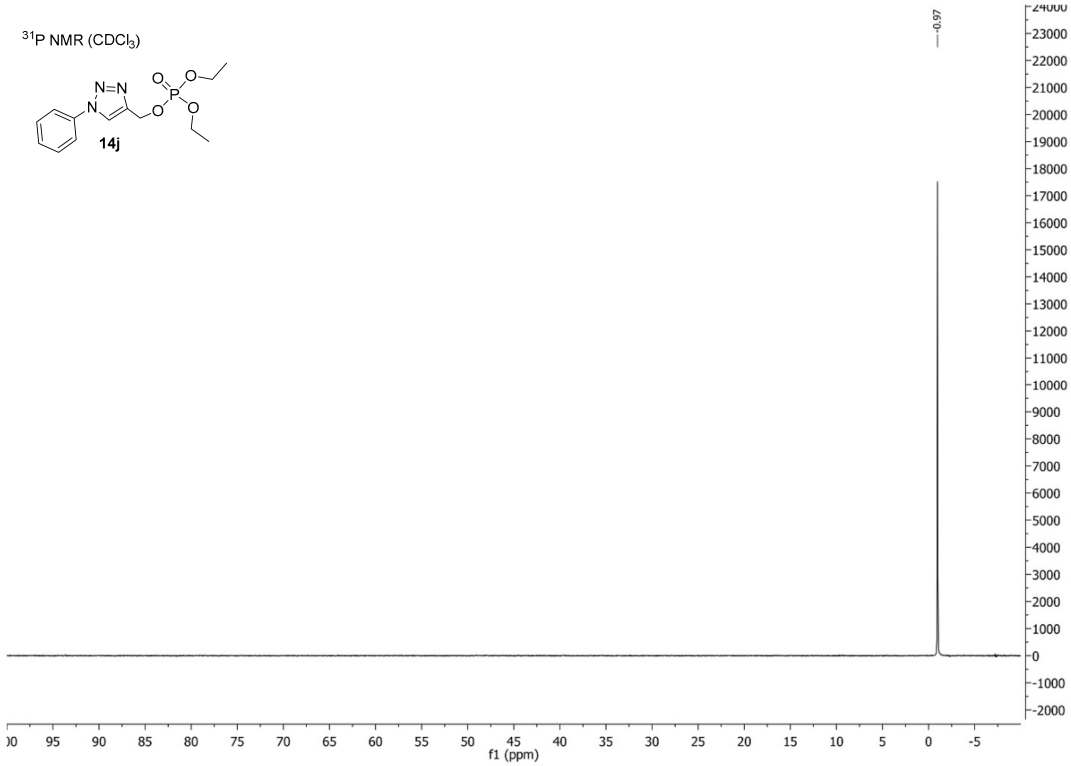

-0.97
^{31}P NMR (CDCl$_3$)
14j
f1 (ppm)

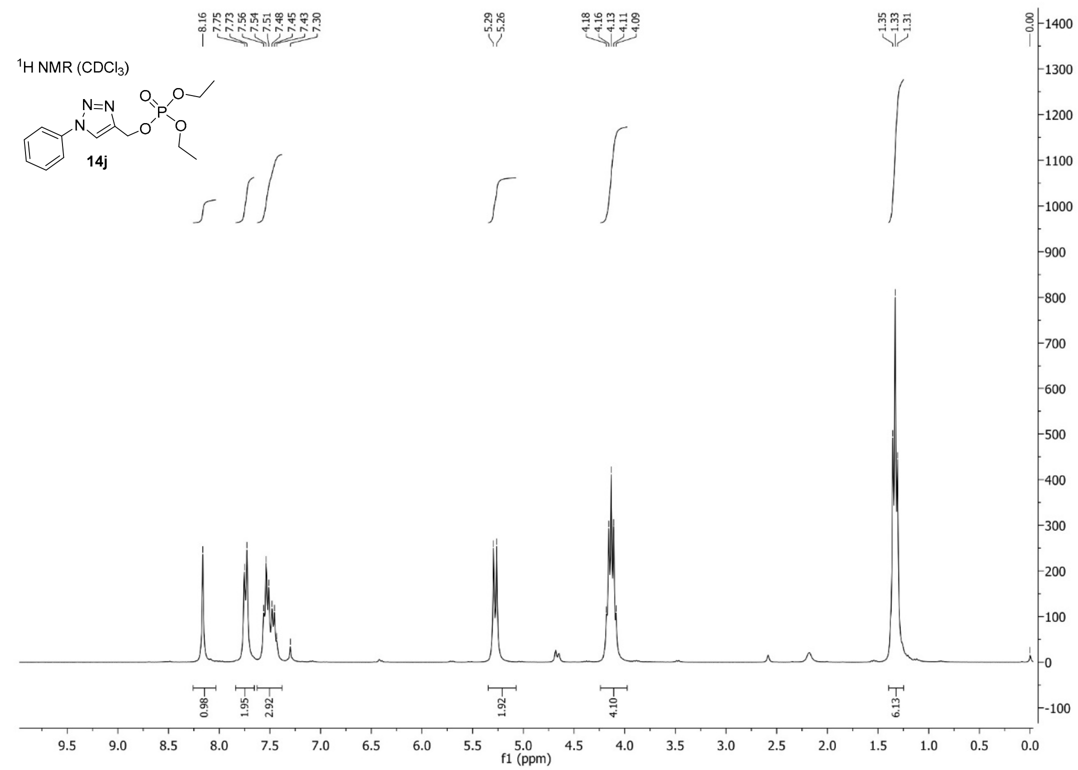

^{1}H NMR (CDCl$_3$)
14j
f1 (ppm)

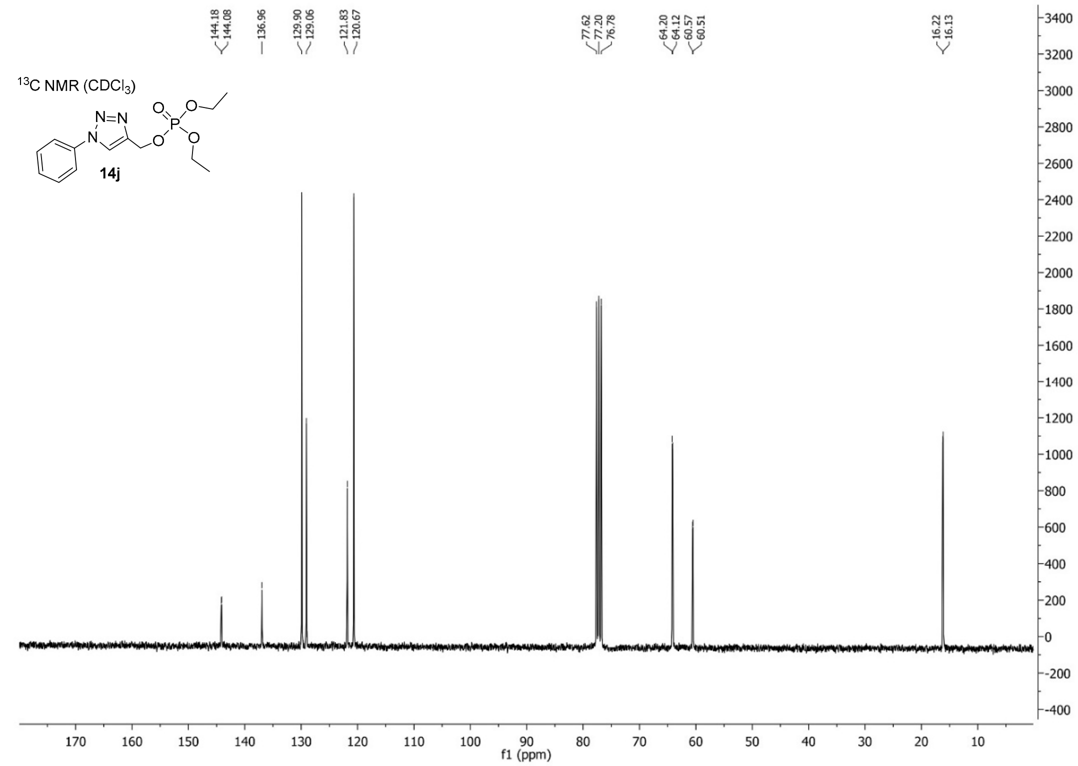

^{13}C NMR (CDCl$_3$)
14j
f1 (ppm)
